# Characterization of human oxidoreductases involved in aldehyde odorant metabolism

**DOI:** 10.1038/s41598-023-31769-4

**Published:** 2023-03-25

**Authors:** Valentin Boichot, Franck Menetrier, Jean-Michel Saliou, Frederic Lirussi, Francis Canon, Mireille Folia, Jean-Marie Heydel, Thomas Hummel, Susanne Menzel, Maria Steinke, Stephan Hackenberg, Mathieu Schwartz, Fabrice Neiers

**Affiliations:** 1 Flavour Perception: Molecular Mechanisms (Flavours), INRAE, CNRS, Institut Agro, Université de Bourgogne Franche-Comté, Dijon, France; 2 CNRS, Inserm, CHU Lille, Institut Pasteur de Lille, UAR CNRS 2014-US Inserm 41-PLBS, University of Lille, Lille, France; 3 UMR 1231, Lipides Nutrition Cancer, INSERM, 21000 Dijon, France; 4 UFR des Sciences de Santé, Université Bourgogne Franche-Comté, 25000 Besançon, France; 5 Plateforme PACE, Laboratoire de Pharmacologie-Toxicologie, Centre Hospitalo-Universitaire Besançon, 25000 Besançon, France; 6 Department of Otolaryngology-Head and Neck Surgery, Dijon University Hospital, 21000 Dijon, France; 7 Smell and Taste Clinic, Department of Otorhinolaryngology, TU Dresden, Dresden, Germany; 8 Chair of Tissue Engineering and Regenerative Medicine, University Hospital Wuerzburg, Roentgenring 11, 97070 Wuerzburg, Germany; 9 Fraunhofer Institute for Silicate Research ISC, Roentgenring 11, 97070 Wuerzburg, Germany; 10 Department of Otorhinolaryngology-Head and Neck Surgery, RWTH Aachen University Hospital, Aachen, Germany

**Keywords:** Oxidoreductases, X-ray crystallography, Oral anatomy

## Abstract

Oxidoreductases are major enzymes of xenobiotic metabolism. Consequently, they are essential in the chemoprotection of the human body. Many xenobiotic metabolism enzymes have been shown to be involved in chemosensory tissue protection. Among them, some were additionally shown to be involved in chemosensory perception, acting in signal termination as well as in the generation of metabolites that change the activation pattern of chemosensory receptors. Oxidoreductases, especially aldehyde dehydrogenases and aldo–keto reductases, are the first barrier against aldehyde compounds, which include numerous odorants. Using a mass spectrometry approach, we characterized the most highly expressed members of these families in the human nasal mucus sampled in the olfactory vicinity. Their expression was also demonstrated using immunohistochemistry in human epitheliums sampled in the olfactory vicinity. Recombinant enzymes corresponding to three highly expressed human oxidoreductases (ALDH1A1, ALDH3A1, AKR1B10) were used to demonstrate the high enzymatic activity of these enzymes toward aldehyde odorants. The structure‒function relationship set based on the enzymatic parameters characterization of a series of aldehyde odorant compounds was supported by the X-ray structure resolution of human ALDH3A1 in complex with octanal.

## Introduction

Olfaction is the major sense that determines flavor perception when eating; it consequently constitutes a key determinant in food intake. In accordance with this, olfactory dysfunction leads to a decrease of food enjoyment and ingestion^1^ or/and reduction in well-being and quality of life sometimes leading to depression^2^. Olfactory sensations are based on binding of odorant molecules on olfactory receptors within the olfactory cleft^3^. Odorant molecules are released in the mouth during chewing and are transported by air to the olfactory receptor via the retronasal route. These receptors are located on the surface of the olfactory cilia, which themselves are bathed in the olfactory mucus^4–8^. Odorant molecules must therefore pass through this mucus, which contains mostly water (95%), mucopolysaccharides (2%), enzymes, glycoproteins, antibodies and salts. Among proteins, odorant binding proteins (OBP) belong to the lipocalin family^9^ and are potential odorant transporters. The nasal mucus contains many other proteins^10^, and recent studies have shown that among these proteins are enzymes metabolizing odorants^11–15^, which participate in olfactory peri-receptor events. These nasal proteins are involved in the protection of cells, including olfactory neurons, against reactive molecules (aldehyde, ester, sulfur compounds, etc.) as a first barrier. As a consequence of this metabolic activity, these enzymes can be involved in olfactory signal termination by facilitating odorant elimination. This elimination constitutes a clearance mechanism that stops the receptor signal and prevents olfactory receptors from saturation. Additionally, it was proposed that the newly created metabolites could modify the olfactory response due to their affinity for olfactory receptors, which can differ from the original molecules^11^. It was demonstrated that metabolization of some odorants in human nasal mucus/saliva resulted in the creation of new aroma compounds affecting the activation pattern of odorant receptors^12,16,17^. The involved proteins are xenobiotic metabolizing enzymes (XMEs) also called odorant metabolizing enzymes (OMEs). Evidence that some of these XMEs also act on odorants has been reported in recent years^13^. The first group of XMEs is phase I enzymes that functionalize odorants with chemical reactions such as oxidation, reduction, and hydrolysis (e.g., cytochrome P450 monooxygenases, alcohol dehydrogenases, aldehyde dehydrogenases, etc.). Their function is to biotransform xenobiotics into more polar metabolites and provide sites for conjugation reactions. The second group is phase II enzymes; these enzymes (UDP-glucuronosyl transferases, glutathione transferases, etc.) can directly act on xenobiotics but commonly conjugate functionalized metabolites with a polar compound to increase odorant hydrophilicity and decrease their reactivity to eliminate them more easily. Phase III proteins include membrane transports in charge of removing hydrophilic xenobiotics from the cells when the process occurs within cells. Proteomic studies have shown the presence of phase I and phase II XMEs in human olfactory mucus and sensory cilia^10,18^. Other studies reported metabolizing activity of phase II glutathione transferases and UDP glucuronosyl transferases on odorant molecules at the olfactory level^19–21^. Additionally, oxidoreduction reactions of odorant molecules after incubation in nasal mucus were reported without identifying these enzymes^12,22^. Other studies have demonstrated that these metabolic reactions are enhanced by the cofactor NAD(P)H in olfactory mucus^23,24^. Oxidoreductases are major phase I enzymes that are NAD(P)H-dependent and are found in many parts of the body due to their detoxification role. For instance, their activity has been demonstrated in the buccal cavity at the salivary^25–27^ and epithelial levels^27,28^. At the olfactory level, they were also shown to be expressed in mouse sensory cilia^18^, rodent olfactory mucus and epithelium^16,19^ and human nasal mucus^10,18,29^.

The present study aims to identify enzymes potentially involved in this odorant metabolizing activity and localize them in nasal mucus and the nasal cavity as well as demonstrate their ability to metabolize odorants. We used mass spectrometry and immunohistochemistry to identify enzymes potentially involved in odorant metabolism. Among them, three candidates, aldehyde dehydrogenase family 1 member A1 (ALDH1A1), aldehyde dehydrogenase family 3 member A1 (ALDH3A1), and aldo–keto reductase family 1 member B10 (AKR1B10), which are already known to detoxicate toxic substrates that we may encounter in daily life, were selected for in vitro validation of the capacity to metabolize odorants. To do this, a panel of odorants was tested on recombinant enzymes, and the tridimensional structure of ALDH3A1 in complex with one of the best-identified odorants was solved.

## Results

### Identification of the oxidoreductases involved in human nasal mucus odorant metabolism

**Figure 1:**
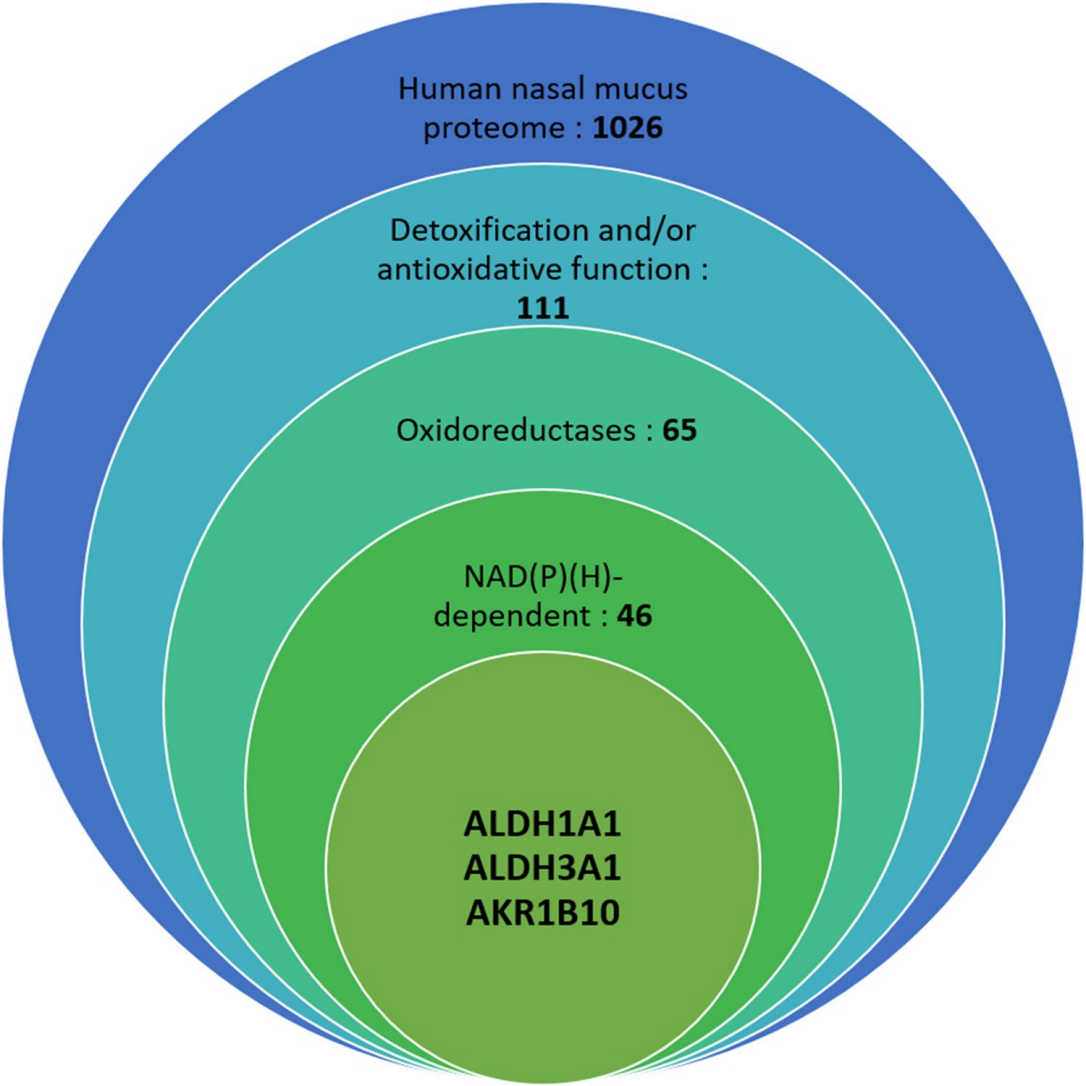
Classification of human nasal mucus proteins. The 1026 proteins identified were first classified using UniProt depending on their detoxification or antioxidative function, then if they are part of the oxidoreductase family, and lastly if they need NAD(P)(H) cofactor. For each category, the number of proteins is indicated.

To identify the most abundant NADH-dependent enzymes in human nasal mucus, we investigated the nasal mucus proteome from three donors by mass spectrometry analysis, leading to the identification of 1026 different proteins. The number of identified proteins was not the same in the three donors, mainly due to the differences in the quality of the three samples. This number is much higher than that in the proteomic analysis of Debat et al.^10^ in 2007, who reported 83 proteins in nasal mucus, and equivalent to that in the analysis of Yoshikawa in 2018^30^, in which 1236 ± 230 proteins were identified in young subjects’ mucus and 1227 ± 274 in elderly subjects’ mucus. In this study, the mucus was picked up in the olfactory cleft using 30° rigid endoscopy. Among the most abundant proteins identified in these proteomes, we identified 111 proteins involved in detoxification or antioxidative mechanisms or both (Fig. 1). Groups were made with UniProt classification according to previous publications, which demonstrated either a role in the detoxification of toxic compounds such as the aldo–keto reductase family 1 member A1 (AKR1A1)^31,32^, a role in the antioxidant capacity by trapping or destroying free radicals such as superoxide dismutase^33^, or both, such as glutathione transferase Mu 2^34–36^. Additionally, enzymes and proteins already shown to be involved in human olfaction were identified in this proteome, including glutathione transferases, GSTP1^20,37,38^ and l-xylulose reductase (DCXR)^16^, and lipocalins, LCN1^39^, LCN2^39^, and OBPIIa^14,40^, also known as odorant binding proteins, as they can bind odorants (Supplemental Table 6).

Among the 1026 proteins detected, 46 NAD(P)(H)-dependent enzymes were identified and classed, as shown in Table 1. The spectra numbers indicate the total number of counts for all peptides included and detected for the same protein. The spectra numbers are dependent on the protein size as well as the peptide stability; they are also highly driven by the protein abundance. The two most represented NAD(P)(H)-dependent oxidoreductases in the mass spectrometry analysis based on spectral number are aldehyde dehydrogenase 1A1, also named retinal dehydrogenase 1 (ALDH1A1), and aldehyde dehydrogenase 3A1, also named dimeric NADP-preferring (ALDH3A1). They also appear at the second and third positions of the most represented proteins in terms of spectra number among the 1026 identified proteins. Interestingly, they appear at higher level (considering the spectra number) compared to well-known mucus proteins as OBPs. In total, 11 aldehyde dehydrogenases were identified in the three human olfactory mucus samples. Aldo–keto reductases are another family of enzymes that have been identified, especially aldo–keto reductase 1B10 (AKR1B10). It is a highly represented aldo–keto reductase among the three tested people, and this human enzyme was reported to efficiently catalyze the oxidation of toxic aldehydes^41^. These three enzymes were selected to determine their role in odorant reduction and oxidation and for further immunolocalization studies on olfactory epithelium samples.

**Table 1:** NAD(P)(H)-dependent oxidoreductases identified in human nasal mucus.

Protein	UniProt accession	Number of identified peptides (number of spectra for donors 1, 2, and 3, respectively)
**Retinal dehydrogenase 1 (ALDH1A1)**	P00352	59 (299, 212, 73)
**Aldehyde dehydrogenase, dimeric NADP-preferring (ALDH3A1)**	P30838	52 (248, 207, 30)
Alcohol dehydrogenase class 4 mu/sigma chain (ADH7)	P40394	35 (100, 77, 14)
Alcohol dehydrogenase 1C (ADH1C or ADH3)	P00326	29 (99, 74, 12)
Peroxiredoxin-5, mitochondrial	P30044	16 (55, 53, 14)
Alcohol dehydrogenase [NADP( +)] (AKR1A1)	P14550	21 (44, 48, 4)
Carbonyl reductase [NADPH] 1	P16152	18 (50, 41, 4)
Protein/nucleic acid deglycase DJ-1	Q99497	23 (49, 36, 6)
4-Trimethylaminobutyraldehyde dehydrogenase (ALDH9A1)	P49189	22 (42, 36, 6)
Thioredoxin reductase 1, cytoplasmic	Q16881	21 (33, 28, 2)
Prostaglandin reductase 1	Q14914	14 (35, 23, 4)
Alcohol dehydrogenase class-3 (ADH5)	P11766	16 (26, 20, 2)
**Aldo–keto reductase family 1 member B10 (AKR1B10)**	O60218	13 (26, 22, 0)
Glutathione reductase, mitochondrial	P00390	18 (15, 29, 4)
Alpha-aminoadipic semialdehyde dehydrogenase (ALDH7A1)	P49419	15 (24, 20, 0)
Aldo–keto reductase family 1 member C2 (AKR1C2)	P52895	12 (23, 19, 0)
Sorbitol dehydrogenase	Q00796	10 (15, 21, 1)
Aldo–keto reductase family 1 member C3(AKR1C3)	P42330	9 (17, 16, 0)
Biliverdin reductase A	P53004	11 (13, 15, 1)
Alcohol dehydrogenase 1B (ADH1B)	P00325	16 (17, 10, 2)
Thioredoxin domain-containing protein 17	Q9BRA2	5 (14, 8, 5)
Aldehyde dehydrogenase, mitochondrial (ALDH2)	P05091	16 (11, 15, 0)
Aldose reductase (AKR1B1)	P15121	8 (15, 10, 1)
Sepiapterin reductase	P35270	11 (15, 11, 0)
Cytosolic 10-formyltetrahydrofolate dehydrogenase (ALDH1L1)	O75891	3 (13, 11, 1)
Quinone oxidoreductase	Q08257	8 (10, 10, 0)
Isocitrate dehydrogenase [NADP], mitochondrial	P48735	10 (5, 14, 0)
Alcohol dehydrogenase 1A (ADH1A)	P07327	16 (9, 7, 1)
Dihydropteridine reductase	P09417	7 (8, 7, 0)
Aldehyde dehydrogenase family 1 member A3 (ALDH1A3)	P47895	10 (5, 9, 0)
Carbonyl reductase [NADPH] 3	O75828	8 (5, 5, 0)
25-Hydroxycholesterol 7-alpha-hydroxylase	O75881	4 (3, 7, 0)
Aflatoxin B1 aldehyde reductase member 2 (AKR7A2)	O43488	6 (3, 7, 0)
Flavin reductase (NADPH)	P30043	3 (4, 5, 0)
Alcohol dehydrogenase 6 (ADH6)	P28332	4 (4, 4, 0)
NADH-cytochrome b5 reductase 2	Q6BCY4	5 (5, 3, 0)
Protein AMBP	P02760	3 (1, 4, 0)
Apoptosis-inducing Factor 2	Q9BRQ8	1 (2, 2, 0)
Aldehyde dehydrogenase family 3 member B1 (ALDH3B1)	P43353	3 (0, 3, 0)
Glutaredoxin-1	P35754	2 (2, 1, 0)
3-Oxo-5-beta-steroid 4-dehydrogenase (AKR1D1)	P51857	1 (2, 1, 0)
Aldehyde dehydrogenase X, mitochondrial (ALDH1B1)	P30837	3 (0, 2, 0)
Aldehyde dehydrogenase family 8 member A1 (ALDH8A1)	Q9H2A2	1 (1, 1, 0)
Retinal dehydrogenase 2 (ALDH1A2)	O94788	3 (1, 0, 0)
3-Hydroxyacyl-CoA dehydrogenase type-2	Q99714	1 (0, 1, 0)
Aldo–keto reductase family 1 member C1 (AKR1C1)	Q04828	13 (0, 0, 0)

The UniProt code access, number of peptides, and number of spectra identified for each donor for each protein. The enzymes selected for further study are indicated in bold.

### Detection of aldehyde dehydrogenase 1A1 (ALDH1A1), aldehyde dehydrogenase 3A1 (ALDH3A1), and aldo–keto reductase 1B10 (AKR1B10) in human olfactory epithelium and turbinate

**Figure 2:**
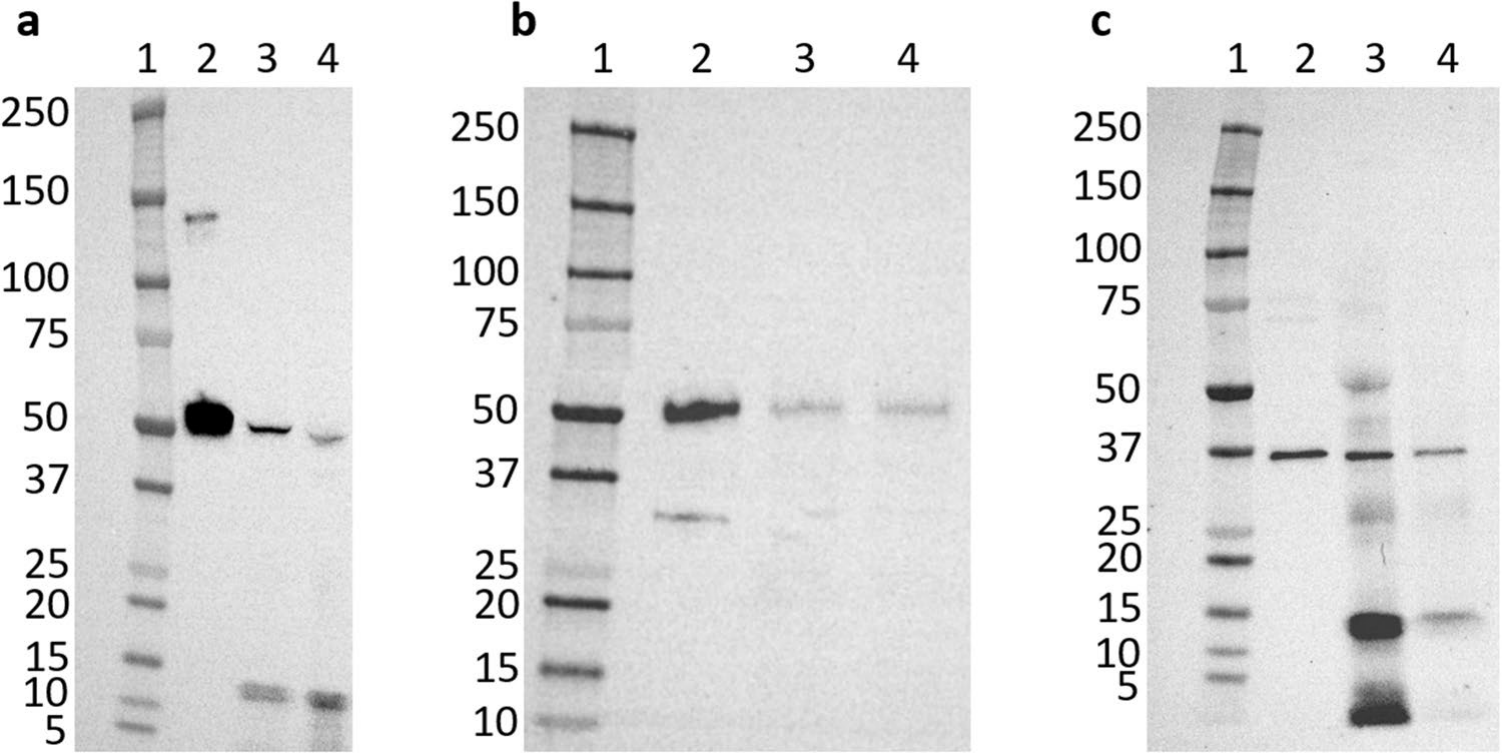
Western blot analysis of oxidoreductases. (**a**) ALDH1A1, (**b**) ALDH3A1 and (**c**) AKR1B10. For the three western blots, the first left column of each gel corresponds to the molecular weight ladder, with markers indicated in kDa (1), the second column corresponds to the human recombinant protein (2), the third column corresponds to tissue from human inferior turbinate (3) and the fourth corresponds to epithelium from human olfactory vicinity (4).

During aging, the respiratory epithelium progressively takes the place of the olfactory epithelium, including turbinates^42^, resulting in a mix between the olfactory and respiratory epithelium at the top of the nasal cavity^43^. Consequently, it is very difficult to obtain only olfactory epithelium due to its small size (between 1 and 2 cm^2^^44^) and restricted access. We selected a human epithelium close to the cribriform plate and a sample from the inferior turbinate. In the majority of cases, the human nose includes three turbinates per side: the superior, middle and inferior turbinates. Western blot analysis supported the expression of these three oxidoreductases (ALDH1A1, ALDH3A1, and AKR1B10) close to the olfactory epithelium, in addition to their expression within the human nasal mucus observed by proteomic analysis. Western blots were performed using sample tissue (human olfactory vicinity epithelium and human inferior turbinate) for each oxidoreductase and are represented in Fig. 2. The three tested oxidoreductases appear to be expressed in the cytosol of the human inferior turbinate and the human olfactory vicinity epithelium. As a positive control, we confirmed that each antibody binds to the corresponding recombinant human enzyme (Lane 2 of Fig. 2A–C). For the three antibodies, the main bands corresponding to the recombinant proteins ALDH1A1, ALDH3A1 and AKR1B10 appeared at the expected theoretical molecular mass of the monomer, 54.9 kDa, 52 kDa, and 36.8 kDa, respectively.

The bands corresponding to the three enzymes were observed at the expected sizes corresponding to the same sizes observed for the corresponding recombinant proteins. In some cases, additional lower bands are observed. These bands could correspond to the degradation of the corresponding enzyme observed for ALDH3A1 in Panel B, probably due to freeze‒thaw cycles of the samples or proteolysis activity prior to conservation. The upper band observed for recombinant ALDH1A1 could correspond to a higher oligomeric state of this enzyme preserved during SDS PAGE.

### Oxidoreductases localization in human olfactory/respiratory epithelium

**Figure 3:**
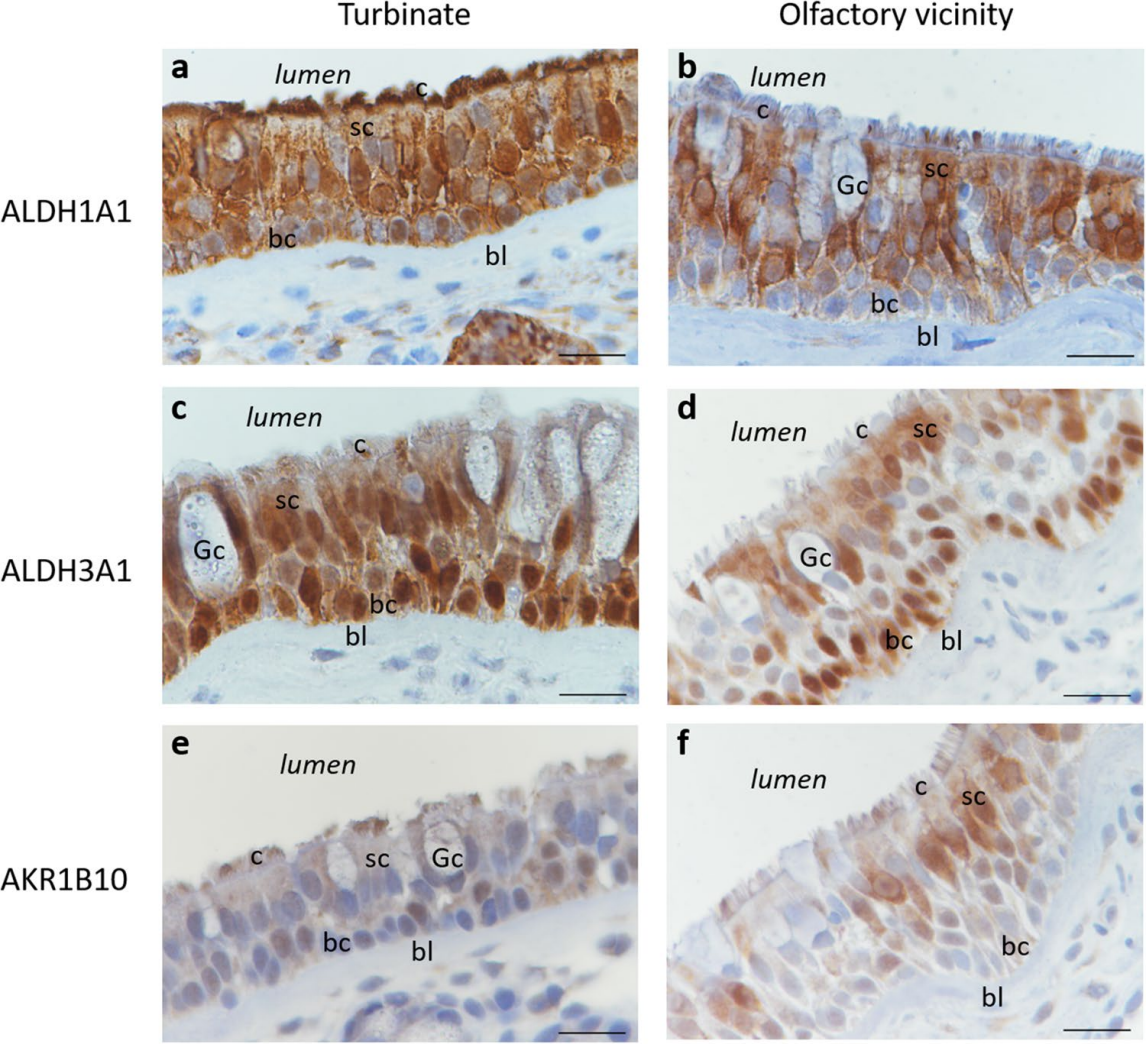
Immunohistochemistry analysis of oxidoreductase expression in the human olfactory cleft. (**a,b**) Primary antibody against ALDH1A1 diluted 5000 times. (**c,d**) Primary antibody against ALDH3A1 diluted 4000 times. (**e,f**) Primary antibody against AKR1B10 diluted 4000 times. *Gc* Goblet cells, *c* ciliated cells, *sc* sustentacular cells, *bl* basal lamina, *bc* basal cells. The scale bar is 20 µm.

To investigate the localization of the three oxidoreductases within the tested tissues of the olfactory cleft, immunohistochemistry was performed to stain ALDH1A1, ALDH3A1, and AKR1B10 in the human olfactory vicinity and human inferior turbinate epithelium from two different human donors. Both the turbinate and olfactory vicinity contain the three oxidoreductases, as supported by the Western blot analysis (Fig. 3). Three oxidoreductases were found in both tissues. Whereas ALDH1A1 and ALDH3A1 showed a relatively high signal intensity, the immunohistochemical signal was much lower for AKR1B10. It appears that the three oxidoreductases are synthesized in the major epithelial cell types, since DAB signals were verified in almost every cell in our samples. Additionally, we identified DAB staining of the three oxidoreductases on the apical, ciliated surface of the samples. This allows for interaction with the molecules, including odorants, which come in contact with and can penetrate these cells. No staining was observed in the goblet cells (Gc) for the three tested enzymes. The absence of the three enzymes in the goblet cells involved in mucus secretion suggests secretion by nasal glands to explain their high expression in the human nasal mucus. In comparison to the oral cavity, ALDH3A1, which is also found in human saliva, is secreted by the salivary glands^45^, as is the case for other aldehyde dehydrogenases^46^. AKR1B10 as ALDH1A1 and ALDH3A1 also lack the conventional signal peptide at the N terminus. However, a molecular chaperone, Hsp90α associates with AKR1B10 (toward a α-helix), then transports it to lysosomes, and is secreted jointly with Hsp90 out of the cell^47^. In this context AKR1B10 can potentially be directly secreted in the mucus by the ciliated cells. Interestingly, Hsp90α is also significantly found (numerous unique peptide and numerous count) in the mucus of the three tested donors (Supplemental Table 6).

### Oxidoreductases metabolize odorant molecules

To test the capacity of the three selected oxidoreductases to oxidize or reduce odorant molecules, the three enzymes were recombinantly produced in *Escherichia coli.* Then, they were purified using chromatography columns to perform enzymatic assays. The three enzymes were obtained at a high level of purity > 98% (Supplemental Fig. 1). The three enzymes used either nicotinamide adenine dinucleotide (NAD^+^, for ALDH1A1 and ALDH3A1) or nicotinamide adenine dinucleotide phosphate (NADPH, for AKR1B10) as cofactors. Consequently, the reaction can be monitored by the reduction of NAD^+^ to NADH or the oxidization of NADPH to NADP, as the reduced forms absorb the light at 340 nm in contrast to the oxidized form.

A panel of twenty odorants all belonging to the aldehyde class were selected. The twenty selected odorant aldehydes are aliphatic or aromatic aldehydes (Table 2). In the presence of NAD^+^, the two ALDHs can catalyze the oxidation of aldehydes into their corresponding carboxylic acids, while AKR catalyzes the reduction of aldehydes into their corresponding alcohols in the presence of NADPH. For each enzyme presented here, a Michaelis response was observed in accordance with the few substrates previously tested in the literature^48,49^ and allowed for calculation of the kinetic parameters (K_M_, k_cat_).

**Table 2:** Metabolization efficiencies of ALDH1A1, ALDH3A1, and AKR1B10 for a panel of 20 aldehydes.

Odorant (scent)	Structure	k_cat_/K_M_ (min^−1^ µM^−1^)			Odorant (scent)	Structure	k_cat_/K_M_ (min^−1^ µM^−1^)		
		ALDH1A1	ALDH3A1	AKR1B10			ALDH1A1	ALDH3A1	AKR1B10
Propanal (winey)		1.7 ± 0.4	nm	nm	Tridecanal (citrus)		14 ± 6	64 ± 28	13 ± 3
Butanal (chocolate)		10 ± 3	1.3 ± 0.2	0.4 ± 0.3	Trans-2-hexenal (green banana)		nm	20 ± 4	5 ± 2
Pentanal (fermented fruity)		30 ± 14	12 ± 2	2.0 ± 0.5	Cis-4-heptenal (creamy)		9 ± 2	74 ± 16	4 ± 1
Hexanal (green)		15 ± 10	102 ± 35	25 ± 6	Trans-2-nonenal (fatty)		7 ± 5	145 ± 51	4 ± 2
Heptanal (green)		12 ± 4	129 ± 51	13 ± 2	Benzaldehyde (almond)		nm	15 ± 1	2 ± 1
Octanal (lemon)		16 ± 4	209 ± 87	15 ± 4	Cinnamaldehyde (cinnamon)		8 ± 6	92 ± 28	5 ± 1
Nonanal (rose)		12 ± 7	164 ± 101	11 ± 3	Hydrocinnamaldehyde (melon)		16 ± 6	169 ± 35	25 ± 7
Decanal (orange)		4 ± 2	33 ± 17	2 ± 1	Isovanillin (phenolic)		nm	5 ± 1	1.1 ± 0.3
Undecanal (citrus)		16 ± 14	24 ± 13	2.6 ± 0.5	Phenylacetaldehyde (honey)		nm	nm	1.5 ± 0.4
Dodecanal (citrus)		11 ± 5	96 ± 79	9 ± 3	Vanillin (vanilla)		nm	nm	45 ± 15

Enzymatic activity was measured spectrophotometrically at 25 °C based on the absorbance of NAD(P)H at 340 nm. Initial rates were calculated using an extinction coefficient of 6220 M^−1^ cm^−1^ for NAD(P)H and fitted to the Michaelis‒Menten equation using SigmaPlot software. Metabolization efficiencies were calculated by dividing the catalytic parameters k_cat_ by K_M_. Data in this table are the average of the two replicates with standard deviations. nm stands for not measurable and means that the calculation of K_M_ value was impossible because the enzymatic activity increased linearly with the substrate concentration.

Both tested ALDHs can metabolize aliphatic or aromatic aldehydes but not with the same efficiency. ALDH3A1 has very high efficiencies in metabolizing medium-chain aliphatic aldehydes such as heptanal, octanal, and nonanal (129, 209, and 164 min^−1^ µM^−1^, respectively) as well as aromatic aldehydes such as hydrocinnamaldehyde (169 min^−1^ µM^−1^) (Supplemental Fig. 2). These high efficiencies are mainly driven by high catalytic constants toward these compounds (between 4000 and 7000 min^−1^), whereas the Michaelis constants are higher than those of ALDH1A1 (Supplemental Table 1). ALDH3A1 metabolizes all the aliphatic aldehydes tested to their carboxylic acid relatives, except propanal. The results also show a progressive increase in the efficiency of ALDH3A1 from butanal up to octanal, where it reaches its maximum, before decreasing for carbon chain lengths above 8 (Table 2). This evolution in efficiency seems to be driven by the Michaelis constant K_M,_ which reaches its minimum for octanal and increases depending on the carbon chain length. The efficiency of ALDH1A1 varies little according to the length of the carbon chain or the presence of an aromatic ring (Table 2), and its maximum efficiency is obtained with pentanal (30 min^−1^ µM^−1^). The k_cat_ of this enzyme is low (approximately 50 min^−1^), which strongly contributes to lower efficiencies toward the tested odorants compared to ALDH3A1. However, the measured K_M_ was the best for ALDH1A1 compared with the two other enzymes (Supplemental Table 1).

AKR1B10 catalyzes the reduction of aldehydes to the corresponding alcohols via the oxidation of NADPH to NADP^+^^41^. AKR1B10 catalyzes the reduction of all the aldehydes tested (Table 2) except propanal. The best efficiencies were measured for hexanal, hydrocinnamaldehyde, and vanillin (25, 25, and 45 min^−1^ µM^−1^, respectively). AKR1B10 metabolizes aliphatic and aromatic aldehydes without large differences in efficiency between the two types.

To better understand the involved molecular interactions between enzymes and odorous compounds during metabolization in the nasal cavity, ALDH3A1, which presents the best efficiency toward odorant molecules as well as good expression within the olfactory mucus and the different epithelium tested within the olfactory vicinity, was studied by crystallography in complex with its better substrates.

### Tridimensional structure of the ALDH3A1/octanal complex

**Figure 4:**
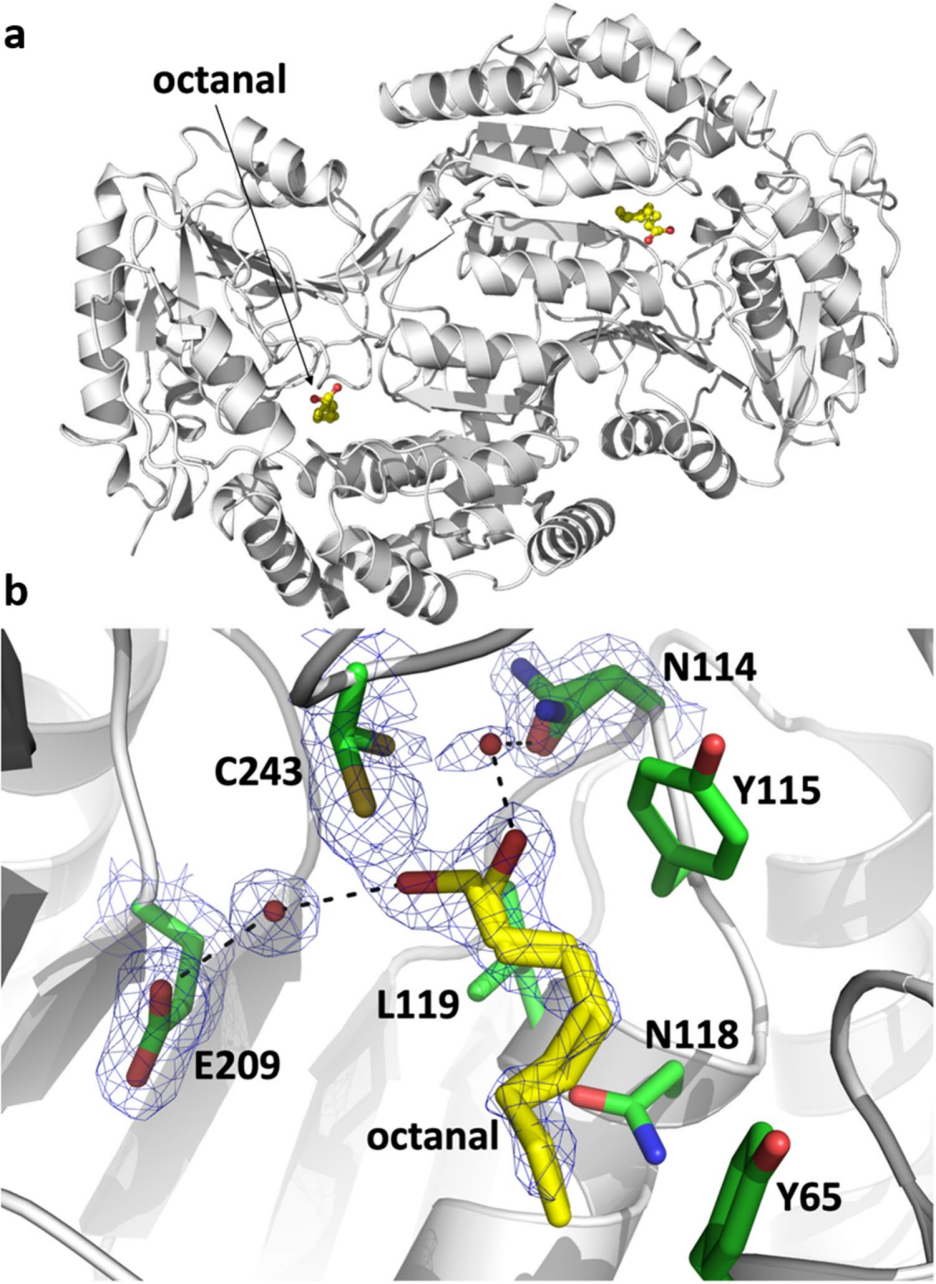
Complex crystal structure of ALDH3A1 bound to octanal. (**a**) ALDH3A1 dimer represented with octanal (yellow). (**b**) ALDH3A1 active site bound to octanal near catalytic cysteine 243. Octanal, Asn 114, and Cys 243 are all present as double conformations, observed from the corresponding *2mFo-DFc* electron density map contoured at 1.2 σ. Side chain residues are shown as green sticks.

To determine the X-ray structure of ALDH3A1 in complex with an odorant, different aldehydes presenting good catalytic efficiency were tested: trans-2-nonenal, hydrocinnamaldehyde, and octanal. Among the different tests, the structure of ALDH3A1-octanal was successfully solved (Fig. 4A). The ALDH3A1 crystal was soaked in its mother liquor containing 10 mM octanal. This resulted in a homodimeric structure at 1.80 angström resolution, where each active site of ALDH3A1 is occupied by one octanal molecule (Fig. 4B). Interpretation of the electron density maps in the active site region near the catalytic Cys 243^50^ led to the conclusion that octanal is present as two alternative conformations. Considering the carbon atoms’ positions, these two conformations are very close, with hydrophobic interactions stabilizing the aliphatic moiety of octanal by the surrounding residues’ side chains (Tyr 65, Tyr 115, Asn 118, Leu 119, Ile 394). This elongated hydrophobic pocket seems well suited for the binding of long-chain aliphatic as well as aromatic aldehydes such as those catalyzed by ALDH3A1, in accordance with our enzymatic analysis. The two conformations of octanal only differ in the positions of the oxygen atom of the aldehyde group. The first conformation of octanal, is hydrogen bonded with Glu 209 via a water molecule. In this case, the side chain of Cys 243 is oriented toward Asn 114 in close vicinity within the active site. The second conformation of octanal is such that its oxygen atom is hydrogen-bonded with Asn 114 via a water molecule. This residue (Asn 114) could be involved in the stabilization of the oxyanionic form of the hemithioacetal state during catalysis. Our structure likely corresponds to the step just before, obtained because of the absence of NAD cofactor needed to complete the catalytic turnover. Thus, the side chain of Cys 243 is oriented toward the octanal molecule, ready for nucleophilic attack. Our results support the role of Cys 243 as a catalytic residue and Asn 114 as a probable catalytically important residue during catalysis.

Taken together, our results show at the molecular level how an odorant aldehyde is metabolized in the ALDH3A1 active site, which is adapted for both aliphatic and aromatic aldehydes.

## Discussion

Aldehyde molecules are found in numerous natural odors; additionally, they are used to enhance a range of fragrance notes. For example, octanal, nonanal, and decanal are commonly used in the perfume industry for their green-floral fragrance^51^. Aldehydes are also frequently encountered in food; indeed, more than 300 food products contain aldehydes as natural constituents or flavoring additives and aromas. Vanillin (vanilla), cinnamaldehyde (cinnamon) and octanal (grape, lemon, peel oil) are the most commonly used compounds^52^. Aldehyde can also have an endogenous origin, synthesized by cells during lipid peroxidation, such as 4-hydroxynonenal^52^, which increases oxidative stress and was already shown to be well metabolized by ALDH3A1^53,54^. 4-hydroxynonenal as well as acetaldehyde, are suspected in the pathogeny of different diseases. In addition, aldehydes in a general manner can be toxic depending on their concentration, which supports the importance of an efficient detoxification system in the most exposed area of the body. In this study, we observed high expression of xenobiotic metabolism enzymes in the human nasal mucus sampled in the olfactory cleft of three different people. Some enzymes identified in these proteomes as GSTs or DCXR were previously shown to be involved in human odor perception^16,20,37,38^. Additionally, new enzymes, potentially involved in odorant metabolization appear as interesting targets for further studies as the sulfotransferase (SULT1A1). Moreover, numerous proteins allowing to maintain the enzyme function as heat shock protein or thioredoxin were also identified. For each person, enzymes involved in aldehyde metabolism were found within the ten most represented proteins in terms of spectra numbers among the 1026 identified proteins. From a larger perspective, 65 oxidoreductase enzymes were identified in the three proteomes, including enzymes involved in aldehyde metabolism as well as enzymes involved in reactive oxygen species reduction. Bathing of the neuron's cilia in the mucus allows for the first step of odorant perception due to the interaction of odorant molecules with receptors located on the membrane of these neurons. Aldehydes are highly toxic to neurons^55^ and need to be particularly protected and continuously renewed for less than one month for rodent olfactory neurons^56,57^. In this context, to safeguard an acute sense of smell, metabolization of aldehyde compounds, including aldehyde odorants, appears essential within the mucus. In this study, glutathione transferase P1, already shown to be able to metabolize aldehyde odorants such as cinnamaldehyde^20^ or to participate in the antioxidant system^58^, appears to be the most expressed glutathione transferase within the mucus. Previous proteomic analysis of the human nasal mucus showed the presence of glutathione transferases; here, we highlight for the first time their abundance, revealing their important expression. Two aldehyde dehydrogenases (ALDH1A1 and ALDH3A1) known to metabolize aldehyde molecules are among the ten highly expressed proteins in the three proteomes. Aldo–keto reductases, including AKR1B10, can also metabolize aldehyde compounds and appear to be well expressed. These three enzymes are also well expressed in the different epithelia tested within the olfactory cleft, as supported by western blot analysis and immunohistochemistry. These last experiments showed good expression of these three enzymes in the different epithelia, including the ciliated cells located at the surface of the epithelia. Consequently, ALDH1A1, ALDH3A1, and AKR1B10 appear to be the first barrier against the toxicity of aldehyde compounds due to their location. It is not excluded that the concentration of these enzymes can be lower in the olfactory epithelium or the mucus secreted by the Bowman glands due to a potential absence of their expression. However, it is most likely that the olfactory epithelium consists of many spots within the respiratory epithelium, consequently the mucus composition will not be dramatically different compared to the one presented in this study (probably a secreted mucus mixture from both tissue types). These three enzymes metabolize various aldehyde odorants with different profiles of efficiency for each enzyme. The twenty tested aldehyde odorants were metabolized. Some were specifically metabolized by a specific enzyme, such as vanillin, which was only metabolized by AKR1B10 or propanal by ALDH1A1, while others were metabolized by the three different enzymes. Their metabolization leads to the formation of the corresponding carboxylic acids (ALDHs) or the corresponding alcohols (AKR), both of which are generally less reactive than their aldehyde precursors. Additionally, 9 other ALDHs and 7 other AKRs were identified in the mucus sampled in the olfactory cleft. To date, 19 ALDHs^59^ and 13 AKRs^60^ have been identified in the human genome, and 11 ALDHs and 8 AKRs were identified in the nasal mucus of the three people tested in this study, showing that most of the oxidoreductase enzymes belonging to these two families are expressed in this mucus. This large representation of these two enzymatic families is not surprising, as olfactory tissues are continuously in contact with volatile organic compounds. This highlights the importance of detoxifying aldehyde odorants in this part of the body. The main evolutive driver to preserve functional enzymes metabolizing aldehyde odorant compounds in the olfactory cleft is probably the tissue and very likely the olfactory neurons preservation. Additionally, these oxidoreductases contribute to human olfaction. Octanal, the aldehyde molecule with the highest catalytic efficiency (among the 20 tested odorant molecules) for ALDH3A1, is more than 10 times more metabolized than the two other tested oxidoreductases. This observation and the high expression of ALDH3A1 in the mucus and in ciliated cells support the major role of this enzyme in octanal metabolization that was previously observed in vivo in human subject breath^61^. ALDH3A1 catalyzes the oxidation of octanal to octanoic acid; however, octanoic acid presents a strong goat cheese odor, which is consequently different from the typical lemon scent characteristic of octanal. The ALDH3A1 active site is well tuned to catalyze octanal oxidation, as revealed by the first structure in complex with a substrate described in this work. The active site configuration supports the enzymatic data obtained for the panel of tested odorant aldehydes. The ALDH3A1 active site includes a hydrophobic entry pocket adapted for the binding of both aromatic and medium-chain aliphatic aldehydes. Their binding, near the catalytic Cys 243 previously shown to be an essential catalytic residue^62^ (2.89 Å between the sulfur of the cysteine and the carbon of the aldehyde functional group), enables further catalysis to yield the corresponding carboxylic acids. In addition to the two ALDHs and the AKR in this study, other isoforms among the 11 ALDHs and the 8 AKRs identified can potentially efficiently metabolize aldehyde odorants, supporting a complex combinatory contribution in human olfaction. ALDH and AKR expression are regulated by dietary habits, supporting an adaptation of their activity toward aldehyde odorants conditioned by these habits^63,64^. Additionally, human oral bacteria are also subject to variations in diet habits, presenting aldehyde activity^65^ and adding potential players in human odorant perception, as already proposed for other oral bacterial activities^66,67^. Interestingly, ALDH2 found during this study in the nasal mucus shows a polymorphism associated with sweet preference^68^, indicating a link of ALDH with flavor perception in a more general manner. In conclusion, this study provides new results regarding the identification of key oxidoreductases involved in human perception in addition to a comprehensive enzymatic analysis of their aldehyde substrates. The structural information obtained in this study clearly supports the role of these enzymes in odorant aldehyde metabolism, which both preserves olfactory tissue and modulates human olfaction.

## Methods

### Chemicals

All odorant compounds were purchased from Sigma-Aldrich (St. Louis, MO, USA). The common name, CAS number, and catalog number are indicated in the supplemental Table 2.

### Preparation of human samples

Tissue from the vicinity of the olfactory region was obtained from 45- and 64-year-old male patients undergoing endoscopic routine sinus surgery. The specimens were taken from the mucosa close to the superior turbinate in direct vicinity to the olfactory area using a 30° rigid endoscope and atraumatic surgical forceps. The patients gave informed consent for participation, and the study was approved by the Ethics Board of the Medical Faculty of Wuerzburg University, Germany (No. 179/17XX). Immediately after harvest, the samples were stored in physiological serum for a few minutes before each sample was separated into two parts in the laboratory. One part was frozen in liquid nitrogen for western blot analysis, and the other was immersed in a buffered fixative solution for immunohistochemistry experiments.

The samples of human nasal mucus were taken from three healthy subjects (aged 31–63, 2 females, 1 male) from the region of the olfactory cleft of both sides with a cotton swab under endoscopic control. The study was performed according to the guidelines of the Declaration of Helsinki and has been formally approved by the Dresden Hospital Ethics Committee.

### Protein assay

Tissues for western blots were defrosted and solubilized in 200 µL of 50 mM Tris–HCl pH 7.5, 250 mM saccharose, and 1 mM EDTA by two tissue-lyser cycles of 60 s each. Then, they were centrifuged for 10 min at 10,000*g* at 4 °C. The supernatants were recovered and ultracentrifuged at 105,000*g* at 4 °C for 60 min to separate soluble cytosol from insoluble microsomes. The microsomes were resuspended in 100 µL of 150 mM Tris–HCl buffer pH 8, and the cytosol and microsome fractions were stored at −80 °C. The protein levels of all fractions were quantified by the Lowry method by using bovine serum albumin as a standard.

### Proteomic data analysis

Raw data collected during nano LC–MS/MS analyses were processed and converted into an *.mgf peak list format with Proteome Discoverer 1.4 (Thermo Fisher Scientific). MS/MS data were analyzed using the search engine Mascot (version 2.4.0, Matrix Science, London, UK) installed on a local server. Searches were performed with a tolerance on mass measurement of 0.2 Da for precursor and 0.2 Da for fragment ions against a composite target-decoy database (20,506 × 2 total entries) built with a human Swissprot database (taxonomy 9606, January 2019, 20,388 entries) fused with the sequences of recombinant trypsin and a list of classical contaminants (118 entries). Cysteine carbamidomethylation, methionine oxidation, protein N-terminal acetylation, and cysteine propionamidation were searched as variable modifications. Up to one missed trypsin cleavage was allowed. The identification results were imported into Proline software (http://proline.profiproteomics.fr) for validation^69^. Peptide spectrum matches taller than nine residues and ion scores > 10 were retained. The false discovery rate was then optimized to be below 1% at the protein level using the Mascot Modified Mudpit score. Spectral counting analyses were performed with Proline 2.0.

### Western blot analysis

Thirty micrograms of protein equivalent of soluble cytosol from the olfactory vicinity epithelium and soluble cytosol from the inferior turbinate and 0.05 µg of recombinant protein were loaded onto a 4–15% precast SDS‒PAGE gel using a Precision Plus Protein™ Dual Xtra Standard molecular weight ladder. Protein migration was performed in tris–glycine-SDS buffer at 200 V for 45 min. The results from the gel were transferred onto a nitrocellulose membrane with a Trans-Blot Turbo Transfer System (Bio-Rad, Hercules, USA). The membrane was then bathed in 0.02 M Tris, 0.15 M NaCl, 0.1% (v/v) Tween 20 at pH 7.6 (TBST) and 5% (w/v) dry milk for 1 h with agitation at room temperature. After five washes in TBST, the membrane was incubated with a dilution of primary antibodies mouse anti-ALDH1A1 (MA5-34924, Thermo Fisher Scientific, Waltham, USA) diluted 1:5000, mouse anti-ALDH3A1 (sc-376089, Santa Cruz Biotechnology, Dallas, USA) diluted 1:1000, and rabbit anti-AKR1B10 (PA5-22036, Thermo Fisher Scientific, Waltham, USA) diluted 1:3000 overnight at 4 °C with agitation. After five washes in TBST, the membrane was incubated for 1 h at room temperature with agitation in TBST with goat anti-mouse HRP secondary antibody (P0447, Agilent, Santa Clara, USA, 1:12,500) for both ALDH types and with the goat anti-rabbit HRP secondary antibody (P0448, Agilent, Santa Clara, USA, 1:12,500) for AKR1B10. After five washes, the membrane was revealed by soaking for one minute in a mixture of 1.5 mL Luminol/enhancer solution and 1.5 mL Peroxide Reagent solution from the ECL clarity western substrate Bio-RadTM pack. The membrane was then placed in a ChemiDocTM acquisition system, and images were acquired by luminescence every 6 s for 10 min and analyzed using Image LabTM 4.0.1 Software (Bio-Rad). A full image for each gel is shown in Supplemental Fig. 3.

### Immunohistochemistry

Turbinate tissues were fixed with formaldehyde solution 4% buffered pH 6.9 (1.00496, Merck, Darmstadt, Germany) for 48 h at room temperature. After decalcification with 10% ethylenediaminetetraacetic acid disodium salt in phosphate-buffered saline pH 7.4 for four weeks with regular changes, the specimens were dehydrated and embedded in paraffin. The olfactory vicinity tissues were fixed in Roti-Histofix (4%, pH 7, Carl Roth, Germany) and embedded in paraffin using the Microm STP 120 Spin Tissue Processor (Thermo, Waltham, USA). Five-micrometer-thick sections were deparaffinized, rehydrated, and stained immunohistochemically. An antigen pretreatment step was carried out using high-temperature antigen unmasking techniques with target retrieval in citrate buffer pH 6.0 (S2369, Agilent, Santa Clara, USA) for 45 min. Endogenous peroxidases were treated with blocking reagent (S2003, Agilent, Santa Clara, USA) for 10 min at room temperature prior to equilibration in 0.05 M Tris–HCl, 0.15 M NaCl, 0.05% Tween 20, pH 7.6. Tissue sections were saturated for 45 min with 10% normal goat serum (G9023, Merck, Darmstadt, Germany) in antibody diluent (S0809, Agilent, Santa Clara, USA) to reduce nonspecific binding.

Sections were then incubated with the same primary antibodies as for the western blots overnight at 4 °C in the antibody diluent; primary antibodies included AKR1B10 diluted 1:4000, ALDH3A1 diluted 1:4000 and ALDH1A1 diluted 1:5000 and at 1:200 for olfactory marker protein (OMP). This last antibody (sc-365818, Santa-Cruz Biotechnology, Dallas, USA) is proposed to be specific toward human olfactory chemosensory neurons.. Tissue sections were then incubated for 1 h at room temperature for ALDH1A1, ALDH3A1 and OMP experiments with the goat anti-mouse HRP secondary antibody (used for the western blots) at 1:200 and with the goat anti-rabbit HRP secondary antibody (used also for the western blot) for AKR1B10 at 1:200. Due to the lack of specificity of the anti-OMP antibody, we could not show any neuronal specific staining (Supplemental Fig. 5).

Negative controls were prepared by replacing the primary antibody with antibody diluent alone (Supplemental Fig. 4). Immunohistochemical staining was performed using a liquid DAB+ substrate chromogen system (K3468, Agilent, Santa Clara, USA). Sections were counterstained with Mayer’s hemalum solution (1.09249, Merck, Darmstadt, Germany). The slides were examined with an Eclipse E600 microscope. Images were acquired with a DS-Ri2 digital camera using the software NIS-Elements Basic Research (all from Nikon, Tokyo, Japan).

### Protein production and purification

The DNA sequences encoding *human* ALDH1A1 (UniProt code P00352), ALDH3A1 (UniProt code P30838), and AKR1B10 (UniProt code O60218) were optimized for expression in *E. coli,* and the sequence GC rate was modified to approximately 50%. They were subcloned into the pET24b, pET22b, and pET26b vectors between the *NdeI* and *SacI* restriction sites. A sequence encoding 6 histidines was added at the N-terminal extremity of ALDH3A1 and AKR1B10 for purification. The bacterial strains *E. coli* BL21(DE3) Star and BL21(DE3) pLysS with the following genotypes, F^–^*omp*T *hsd*S_B_ (r_B_^–^, m_B_^–^) *gal dcm rne*131 (DE3) and pLysS F^–^*omp*T *hsd*S_B_ (r_B_^–^, m_B_^–^) *gal dcm* (DE3) pLysE(Cam^R^), were used to express both ALDHs and AKR1B10. The transformed cells were grown at 37 °C in LB medium (containing 100 μg mL^−1^ ampicillin) and induced by the addition of isopropyl β-d-1-thiogalactopyranoside (IPTG) when the cell culture reached the selected OD measured at 600 nm. The IPTG concentration, time, and temperature of growth after induction changes between the three proteins are summarized in Supplemental Table 3. Bacterial growth was stopped by centrifugation (4000*g*, 15 min), and bacteria were suspended in Tris buffer containing 50 mM saccharose at 250 mM pH 8.0. Cells were sonicated at 4 °C and centrifuged at 24,000×*g* for 45 min at 4 °C. The recombinant proteins within the supernatant were purified in a first purification step consisting of two successive ammonium sulfate precipitations. Then, the salt was eliminated by two dialyzes in the appropriate buffer for each protein of interest. Two chromatography steps, indicated in Supplemental Table 4, were performed for each enzyme to obtain the pure protein (Supplemental Fig. 1). Proteins were stored at −20 °C.

### Enzymatic assays

Enzymatic activity was determined on a UV-1800 spectrophotometer (Shimadzu, Japan) by measuring the absorbance at 340 nm, which corresponds to the NAD(P)H absorbance wavelength. Enzymatic reactions were performed in a 1 mL quartz cuvette filled with 1 mL of a mixture containing 100 mM KPi buffer pH 7.0, a saturating concentration of cofactor, which was 1 mM NAD for both ALDH and 200 µM NADPH for AKR1B10, an odorous molecule diluted in methanol, 500 nM ALDH1A1 or 70 nM ALDH3A1 or 250 nM AKR1B10, and water to volume. Each experiment was repeated two times. The initial velocities for an increasing range of odorant concentrations were measured, and the Michaelis‒Menten curve was plotted using SigmaPlot software according to the equation vi = (Vmax × [S])/(K_M_ + [S]), where vi is the initial rate in µM min^−1^, Vmax is the maximum initial rate in µM min^−1^, [S] is the substrate concentration in mol L^−1^, and K_M_ is the Michaelis constant in µM. The catalytic constant k_cat_ was obtained by dividing Vmax by the enzyme concentration. The efficiency (k_cat_/K_M_) was obtained by dividing k_cat_ by their corresponding K_M_. Standard errors (Δ) of the efficiency were calculated using the equation: $\Delta efficiency = efficiency \times \sqrt{\left({\left(\frac{\Delta {k}_{cat}}{{k}_{cat}}\right)}^{2}+{\left(\frac{\Delta {K}_{M}}{{K}_{M}}\right)}^{2}\right)}.$ The averages of each kinetic parameter and standard error were calculated and are summarized in Supplemental Table 1. When the enzymatic activity did not follow a Michaelis-response (due to a high K_M_ value), the absorbance increased linearly with substrate concentrations, making the calculation of the K_M_ value impossible; this is represented by “nm” for “not measurable” in Table 2 and Supplemental Table 1.

### Crystallization and X-ray diffraction experiments

Crystallogenesis tests were undertaken with enzymes that had a purity level greater than 98% according to an estimate by SDS‒PAGE gel (Supplemental Fig. 1).

Before the crystallization assays, ALDH3A1 was dialyzed against 10 mM pH 7.8 HEPES buffer. Crystallization trials were performed manually at 20 °C by using the sitting drop vapor diffusion method. ALDH3A1 (2 mg ml^−1^) was crystallized by mixing 1 µL of protein with 1 µL of a solution containing 18% PEG 3350 in 0.1 M potassium acetate pH 7.5 buffer. To obtain complexes of ALDH3A1 with octanal, crystals were soaked into the mother liquor plus 10 mM octanal. Cryoprotection was achieved by adding 20% glycerol to the drops containing the crystals. The crystals were flash-frozen in liquid nitrogen before synchrotron data collection. Diffraction experiments were performed on the SOLEIL synchrotron beamline PROXIMA1. Crystals of ALDH3A1-octanal diffracted to 1.80 Å. The datasets were indexed and integrated with XDS^70^ and scaled with pointless^71^. The structure was solved by molecular replacement using the coordinates of the unbound form of ALDH3A1 (PDB code 3SZA). The 3D structure was manually adjusted with COOT^72^ and refined with PHENIX^73^. Inspection of the electron density maps around the active site region allowed for the identification and building of ligands. Restraint files for ligand refinement were generated with the GRADE webserver (http://grade.globalphasing.org). The structure was validated with MolProbity^74^. The figure was prepared using PyMOL (The PyMOL Molecular Graphics System, Version 2.0 Schrödinger, LLC). The coordinates, structure factors, and diffraction statistics (Supplemental Table 5) have been deposited in the Protein Data Bank under accession codes 8BB8 (ALDH3A1-octanal).

## Supplementary Information

Supplementary Information.

## Data Availability

All data generated or analysed during this study are included in this published article and its supplementary information files. The crystal structure of the ALDH3A1-octanal complex is accessible under the PDB code 8BB8 (https://www.rcsb.org/).
