# Supplementary material for: Characterization of human oxidoreductases involved in aldehyde odorant metabolism
Source: Sci Rep. 2023 Mar 25;13:4876. doi: 10.1038/s41598-023-31769-4 (PMC10039900; doi:10.1038/s41598-023-31769-4)
Supplement: Supplementary file 1 — Supplementary Information. [file 41598_2023_31769_MOESM1_ESM.docx]

Supplemental

Characterization of human oxidoreductases involved in aldehyde odorant metabolism

Valentin Boichot^1^, Franck Menetrier^1^, Jean-Michel Saliou^2^, Frederic Lirussi^3,4,5^, Francis Canon^1^, Mireille Folia^6^, Jean-Marie Heydel^1^, Thomas Hummel^7^, Susanne Menzel^7^, Maria Steinke^8,9^, Stephan Hackenberg^10^, Mathieu Schwartz^1,*^, Fabrice Neiers^1,*^


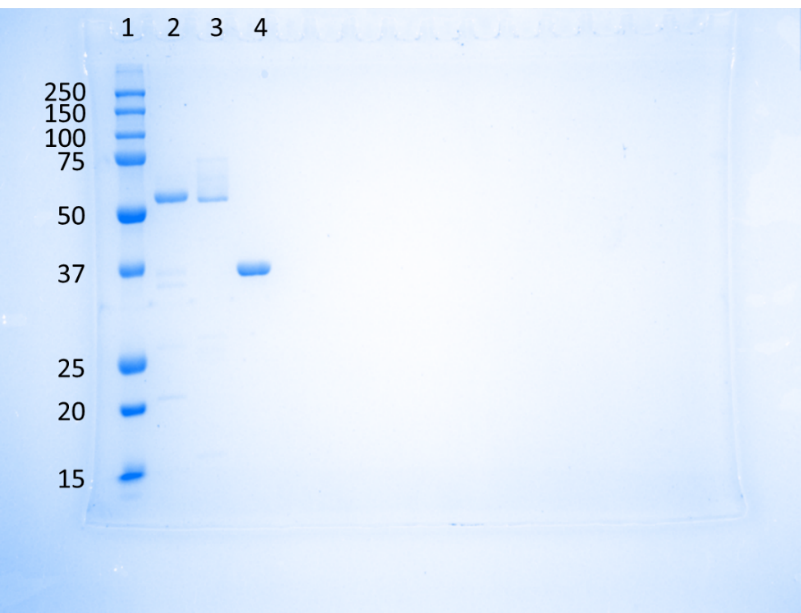
**Supplemental Figure 1.** **Migration of recombinant proteins on 12% SDS‒PAGE gel.** (1) Molecular weight ladder with markers indicated in kDa (2) ALDH1A1 54.8 kDa, (3) ALDH3A1 52 kDa, (4) AKR1B10 36.8 kDa. Each band corresponds to 1 µg of protein.


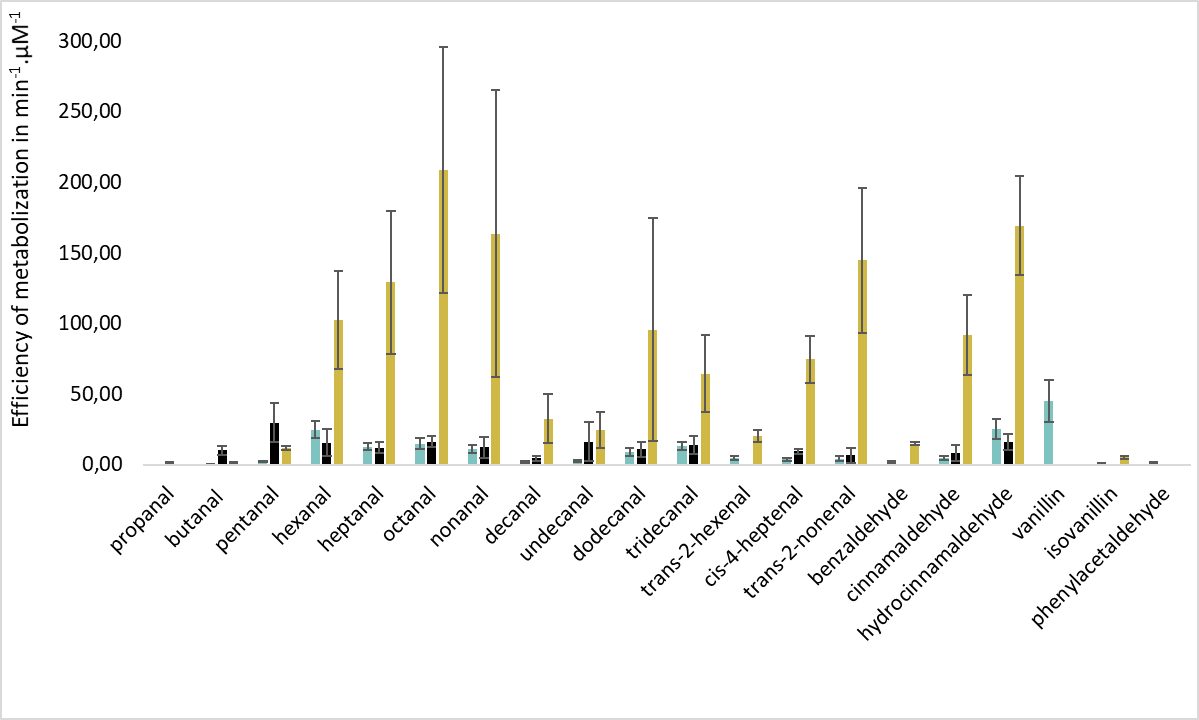
**Supplemental Figure 2.** E**fficiency of metabolism in min^-1^.µM^-1^ of ALDH1A1 (black), ALDH3A1 (yellow), and AKR1B10 (cyan) for 20 odorant aldehydes.** The efficiency was calculated by dividing the catalytic constant k_cat_ by the Michaelis constant K_M_


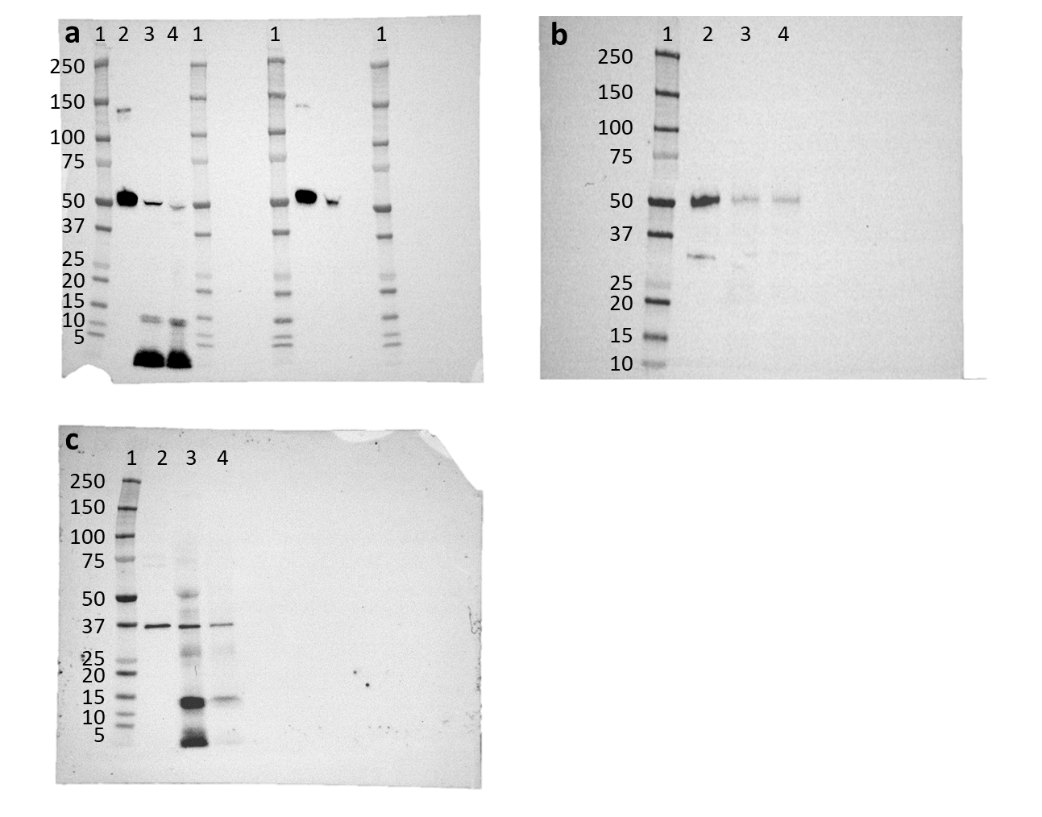


**Supplemental Figure 3.** **Full western blot.** (a) ALDH1A1, (b) ALDH3A1 and (c) AKR1B10. For the three western blots, the first left column of each gel corresponds to the molecular weight ladder, with markers indicated in kDa (1), the second column corresponds to the human recombinant protein (2), the third column corresponds to tissue from human inferior turbinate (3) and the fourth corresponds to epithelium from human olfactory vicinity (4). Other dilutions are tested on other lines on (a).


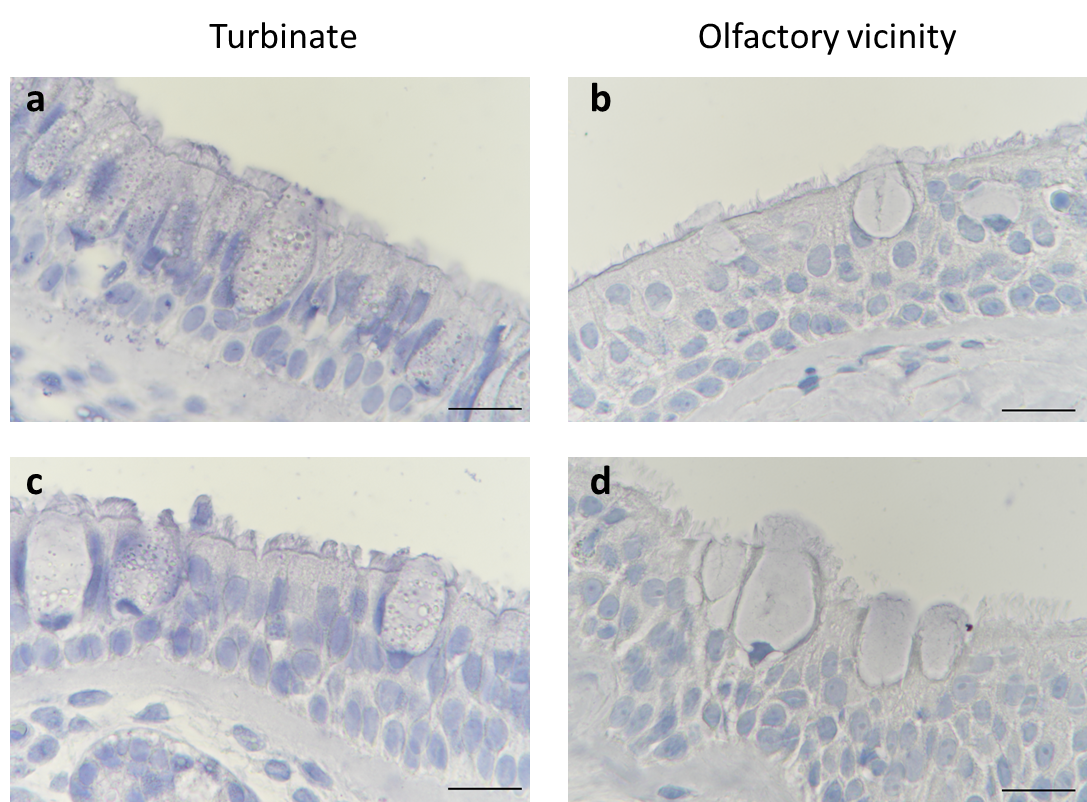
**Supplemental Figure 4.** **Negative control of immunohistochemistry analysis.**

The absence of positive staining is observed in both turbinate and olfactory vicinity epithelium using. (a, b) the goat anti-mouse HRP secondary antibody and (c, d) the goat anti-rabbit HRP secondary antibody

**
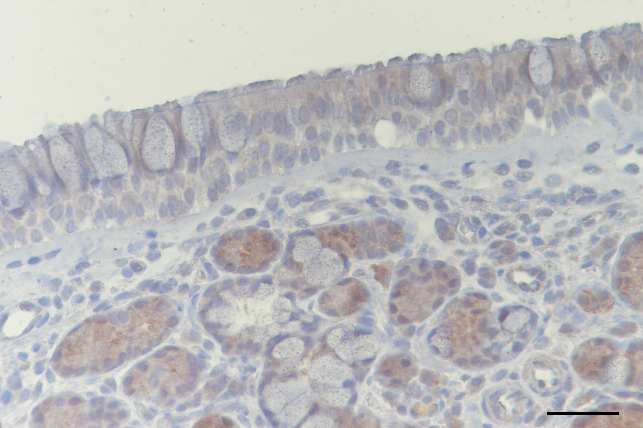
**

**Supplementary Figure 5.** **Immunohistochemistry analysis of OMP expression in the human olfactory cleft.**

Any specific neuronal staining was observed in the different analysis. The scale bar is 20 µm.

| **Odorant**  (scent) | **Structure** | **k_cat_ (min^-1^)** | | | **K_M_ (µM)** | | | **k_cat_/K_M_ (min^-1^.µM^-1^)** | | |
| --- | --- | --- | --- | --- | --- | --- | --- | --- | --- | --- |
|  |  | **ALDH1A1** | **ALDH3A1** | **AKR1B10** | **ALDH1A1** | **ALDH3A1** | **AKR1B10** | **ALDH1A1** | **ALDH3A1** | **AKR1B10** |
| **Propanal**  (winey) | 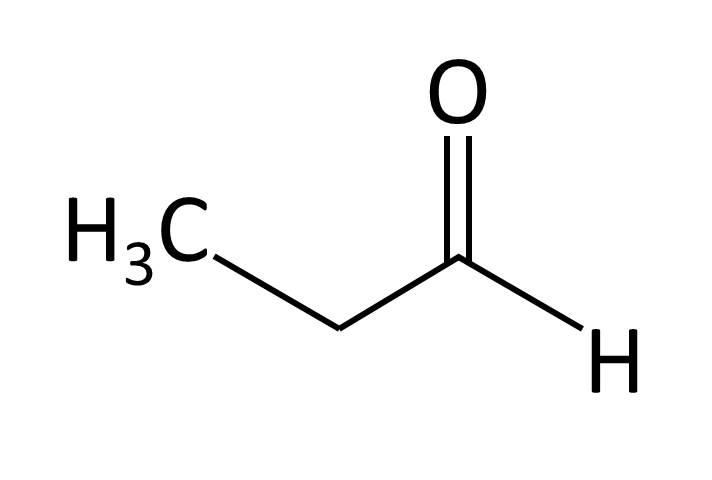 | 102 ± 7 | nm | nm | 62 ± 13 | nm | nm | 1.7 ± 0.4 | nm | nm |
| **Butanal**  (chocolate) | 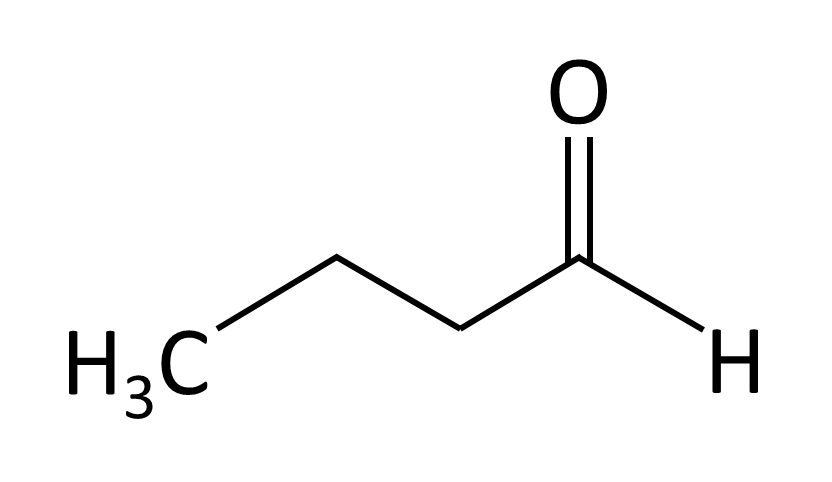 | 34 ± 2 | 5371 ± 429 | 116 ± 33 | 4 ± 2 | 4137 ± 627 | 266 ± 72 | 10 ± 3 | 1.3 ± 0.2 | 0.4 ± 0.3 |
| **Pentanal**  (fermented fruity) | 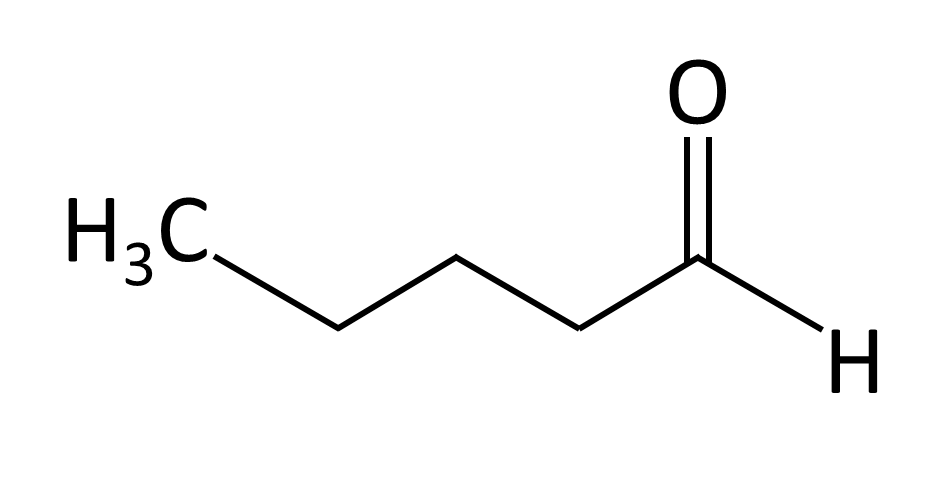 | 111 ± 11 | 4980 ± 214 | 198 ± 19 | 5 ± 2 | 451 ± 59 | 144 ± 46 | 30 ± 14 | 12 ± 2 | 2.0 ± 0.5 |
| **Hexanal**  (green) | 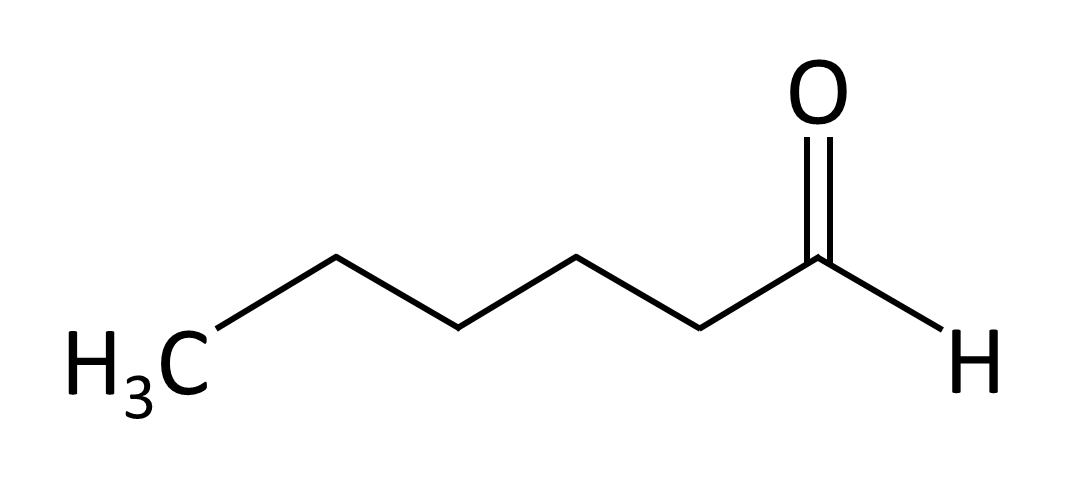 | 41 ±9 | 4847 ± 507 | 683 ± 57 | 3 ± 2 | 49 ± 18 | 28 ± 6 | 15 ± 10 | 102 ± 35 | 25 ± 6 |
| **Heptanal**  (green) | 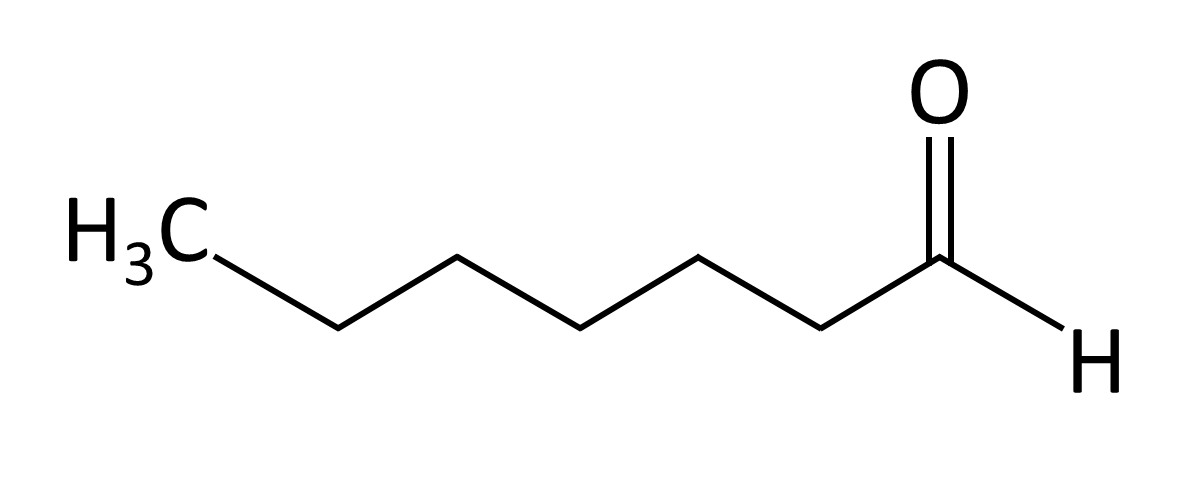 | 51 ± 4 | 1704 ± 203 | 366 ± 30 | 5 ± 1 | 13 ± 5 | 65 ± 20 | 12 ± 4 | 129 ± 51 | 13 ± 2 |
| **Octanal**  (lemon) | 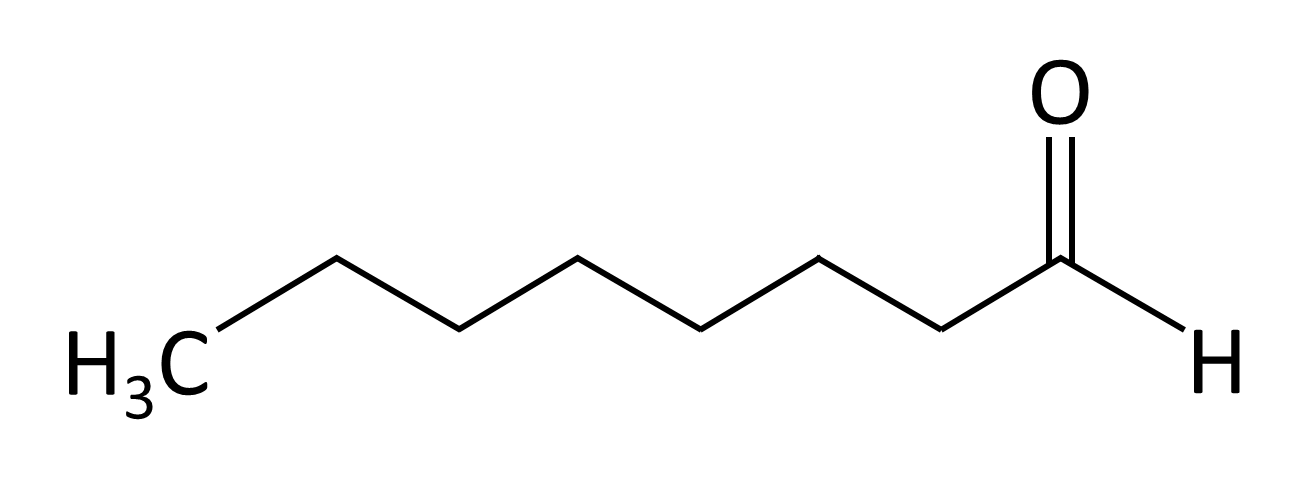 | 51 ± 3 | 1450 ± 140 | 405 ± 37 | 3 ± 1 | 7 ± 3 | 30 ± 6 | 16 ± 4 | 209 ± 87 | 15 ± 4 |
| **Nonanal**  (rose) | 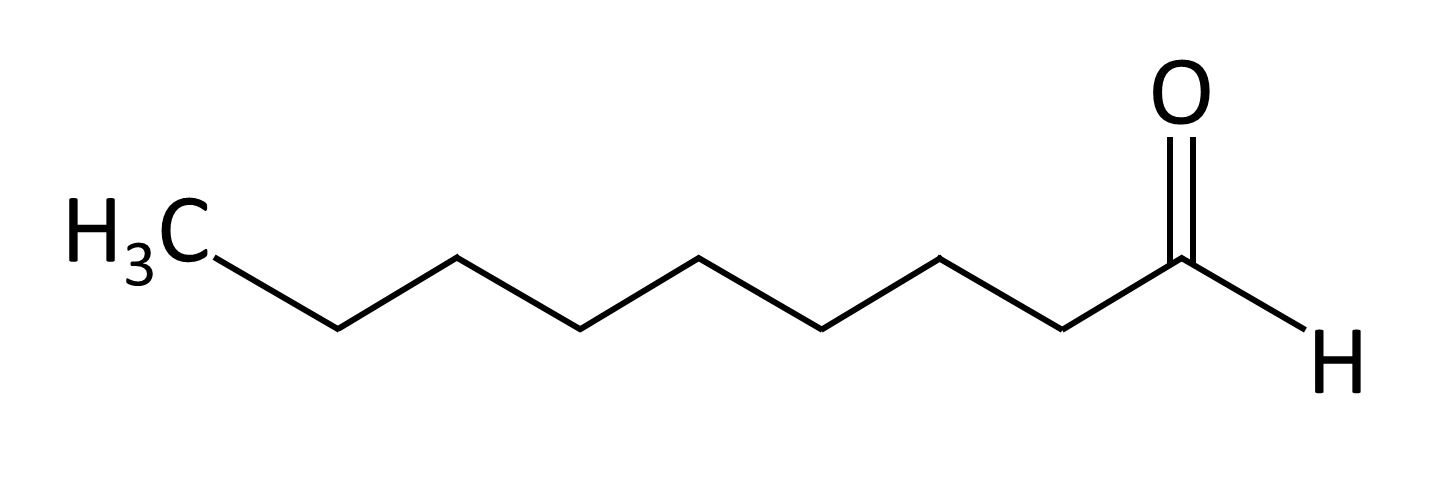 | 67 ± 20 | 1460 ± 263 | 556 ± 58 | 6 ± 5 | 8 ± 5 | 71 ± 24 | 12 ± 7 | 164 ± 101 | 11 ± 3 |
| **Decanal**  (orange) | 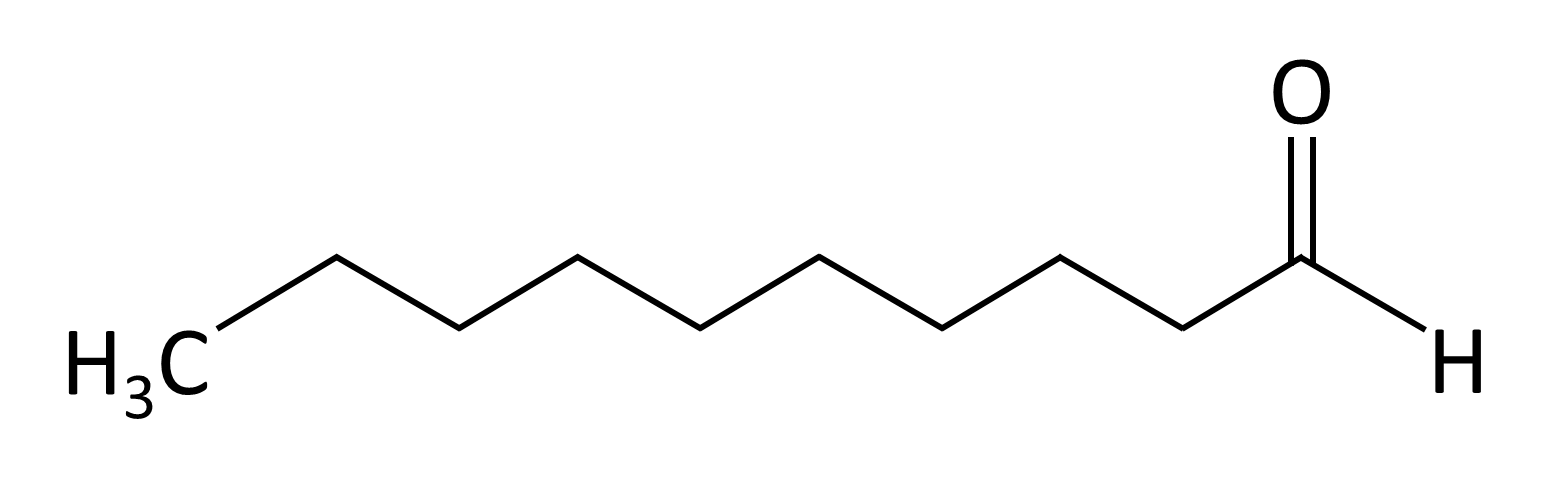 | 88 ± 9 | 1462 ± 249 | 497 ± 75 | 21 ± 8 | 47 ± 21 | 293 ± 84 | 4 ± 2 | 33 ± 17 | 2 ± 1 |
| **Undecanal**  (citrus) | 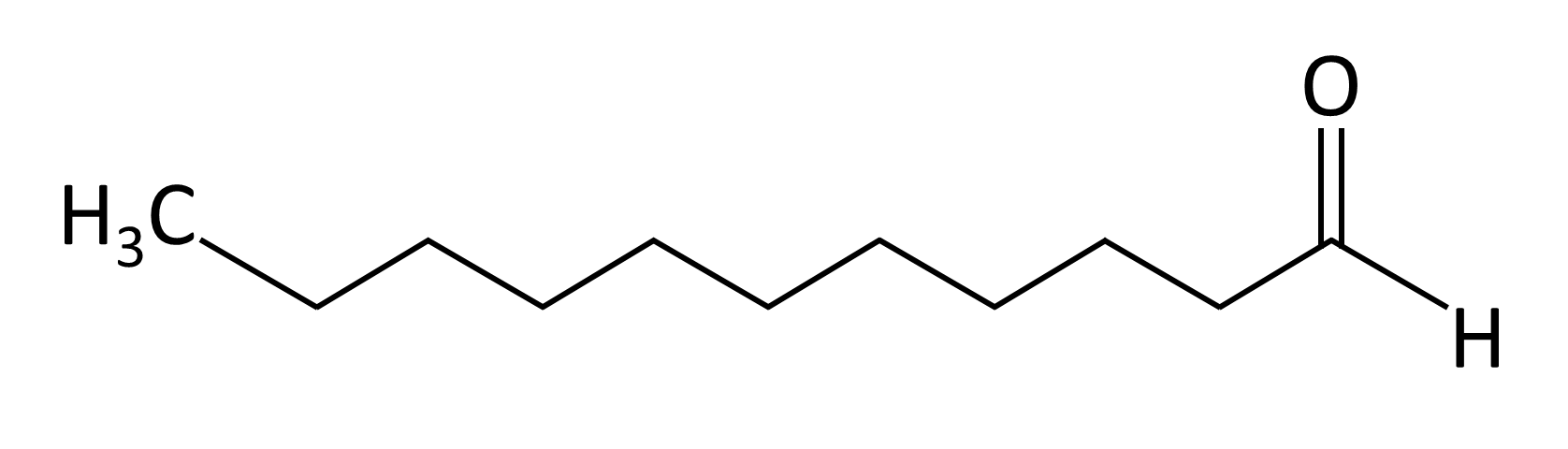 | 39 ± 4 | 1120 ± 278 | 319 ± 21 | 3 ± 2 | 47 ± 25 | 123 ± 22 | 16 ± 14 | 24 ± 13 | 2.6 ± 0.5 |
| **Dodecanal**  (citrus) | 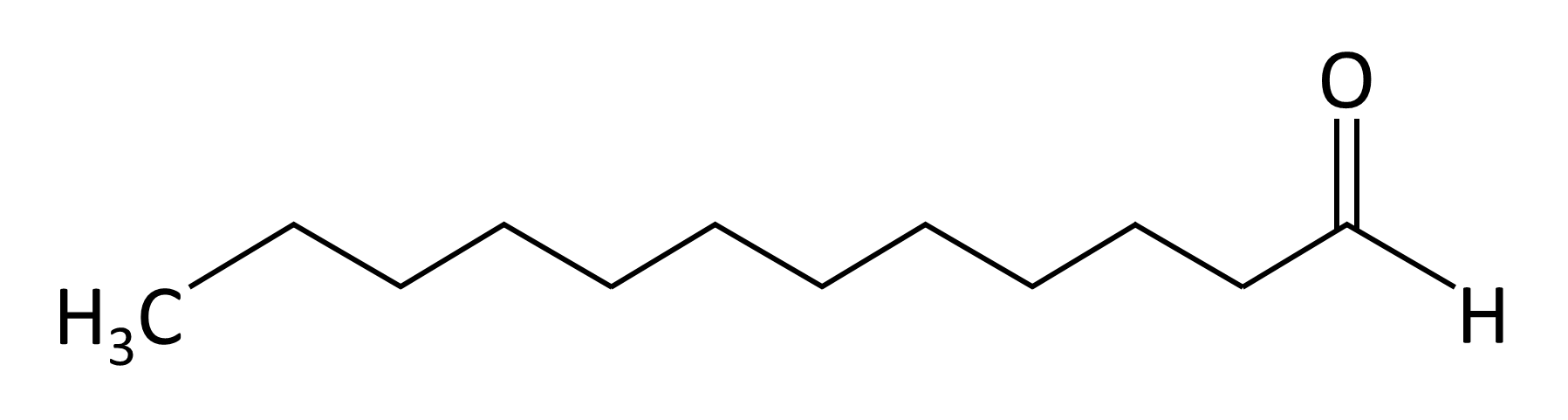 | 39 ± 4 | 896 ± 152 | 341 ± 34 | 4 ± 2 | 9 ± 8 | 40 ± 13 | 11 ± 5 | 96 ± 79 | 9 ± 3 |
| **Tridecanal**  (citrus) | 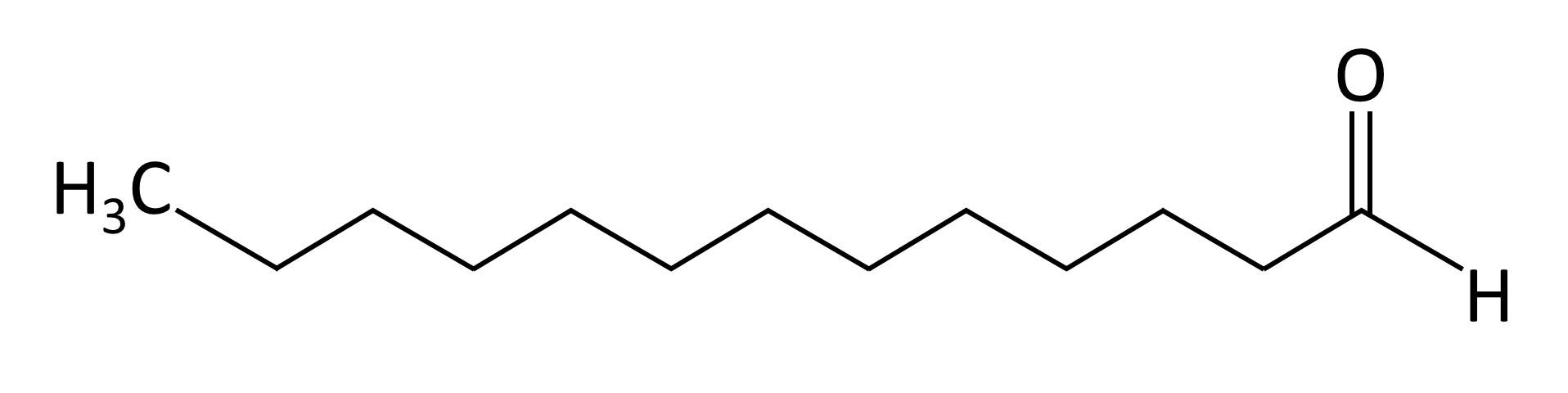 | 56 ± 7 | 725 ± 83 | 238 ± 13 | 4 ± 2 | 11 ± 4 | 18 ± 4 | 14 ± 6 | 64 ±28 | 13 ± 3 |
| **Trans-2-hexenal**  (green banana) | 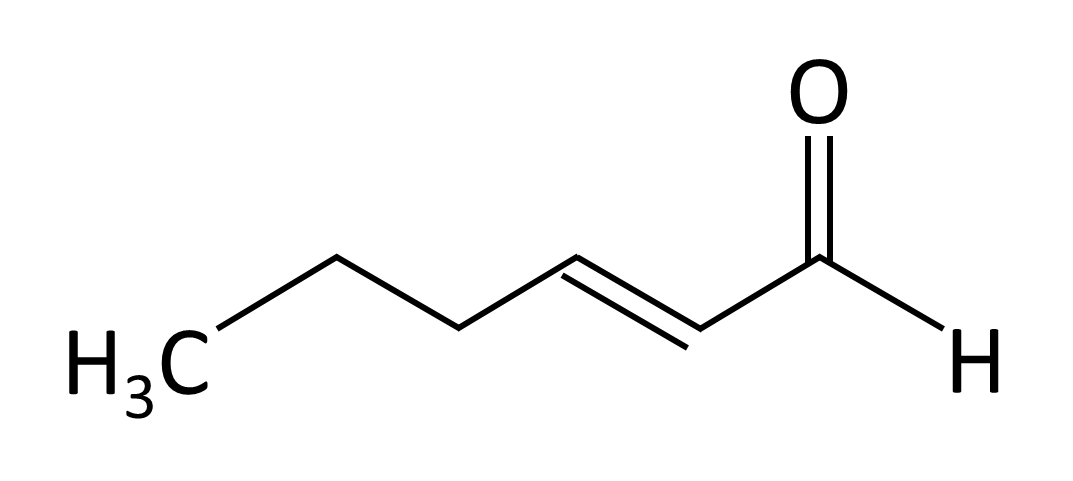 | nm | 1181 ± 117 | 140 ± 14 | nm | 58 ± 12 | 27 ± 9 | nm | 20 ± 4 | 5 ± 2 |
| **Cis-4-heptenal**  (creamy) | 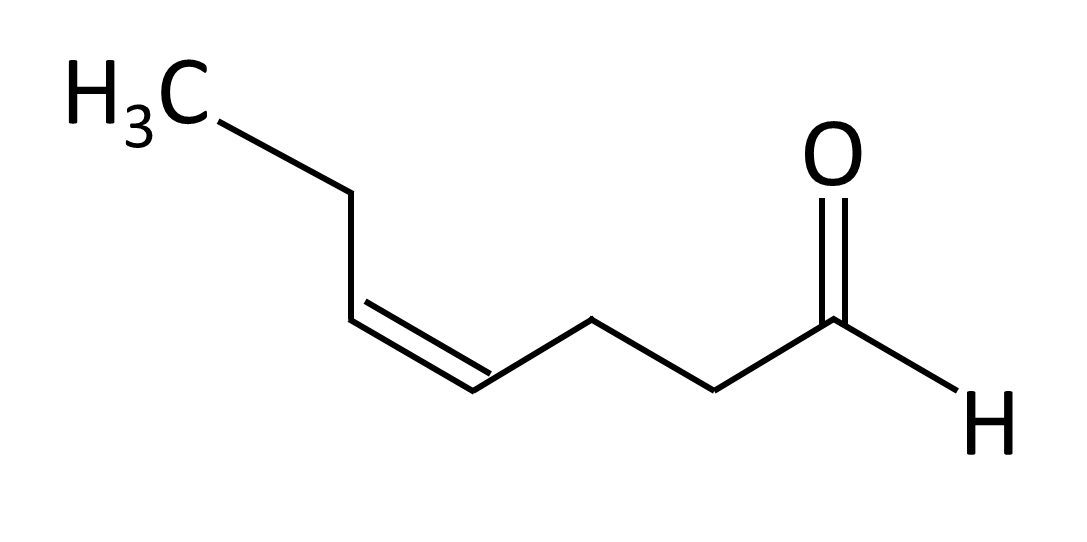 | 76 ± 4 | 3170 ± 212 | 393 ± 39 | 8 ± 2 | 43 ± 9 | 161 ± 42 | 9 ± 2 | 74 ± 16 | 4 ± 1 |
| **Trans-2-nonenal**  (fatty) | 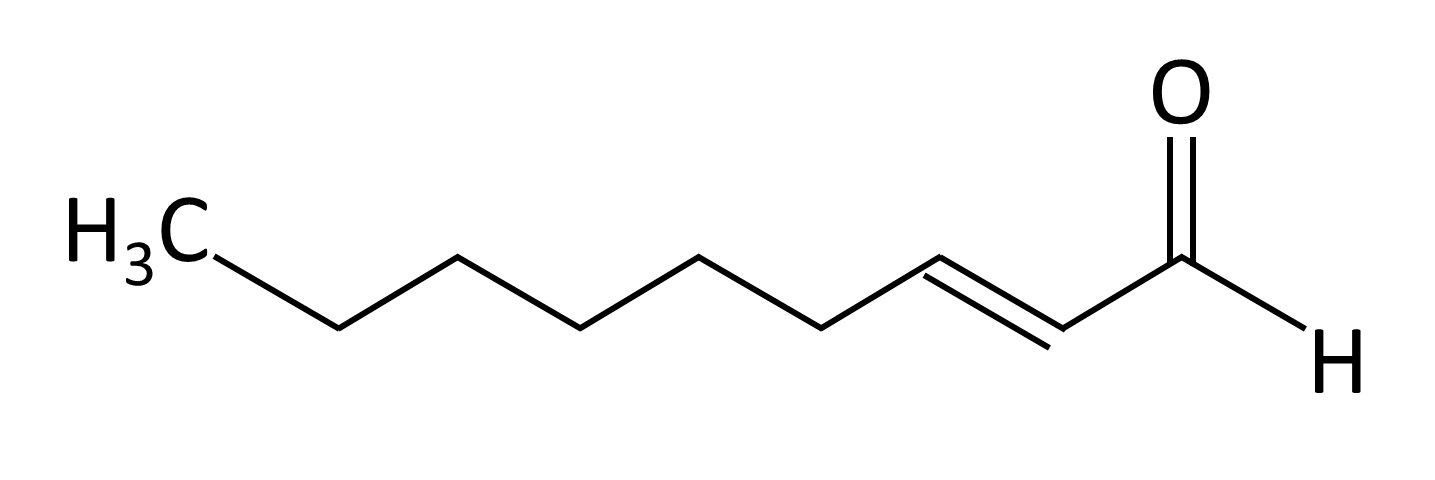 | 18 ± 3 | 4500 ± 729 | 53 ± 7 | 6 ± 3 | 39 ± 18 | 14 ± 9 | 7 ± 5 | 145 ± 51 | 4 ± 2 |
| **Benzaldehyde**  (almond) | 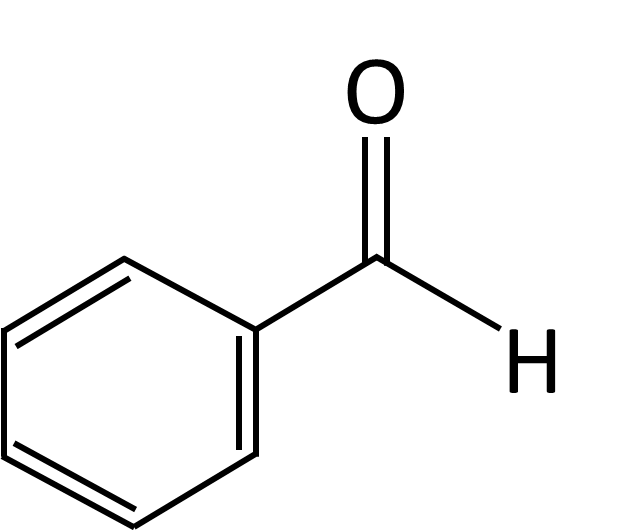 | nm | 2169 ± 65 | 104 ± 13 | nm | 153 ± 12 | 59 ± 23 | nm | 15 ± 1 | 2 ± 1 |
| **Cinnamaldehyde**  (cinnamon) | 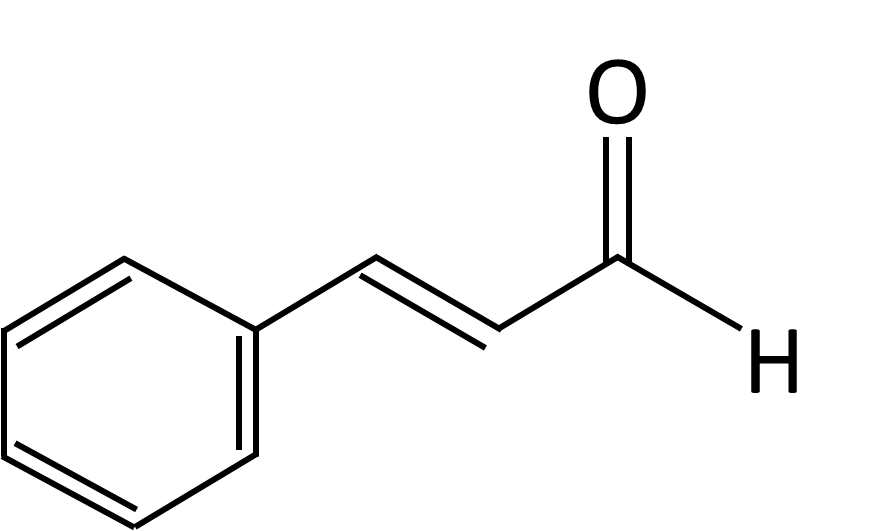 | 24 ± 4 | 5009 ± 507 | 445 ± 40 | 3 ± 2 | 55 ± 16 | 90 ± 20 | 8 ± 6 | 92 ± 28 | 5 ± 1 |
| **Hydrocinnamaldehyde**  (melon) | 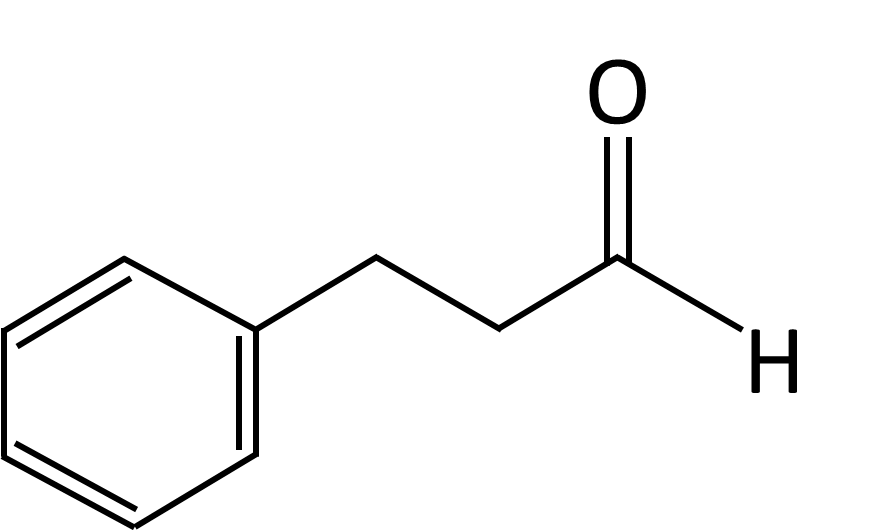 | 64 ± 4 | 3489 ± 197 | 322 ± 31 | 4 ± 1 | 21 ± 4 | 22 ± 7 | 16 ± 6 | 169 ± 35 | 25 ± 7 |
| **Isovanillin**  (phenolic) | 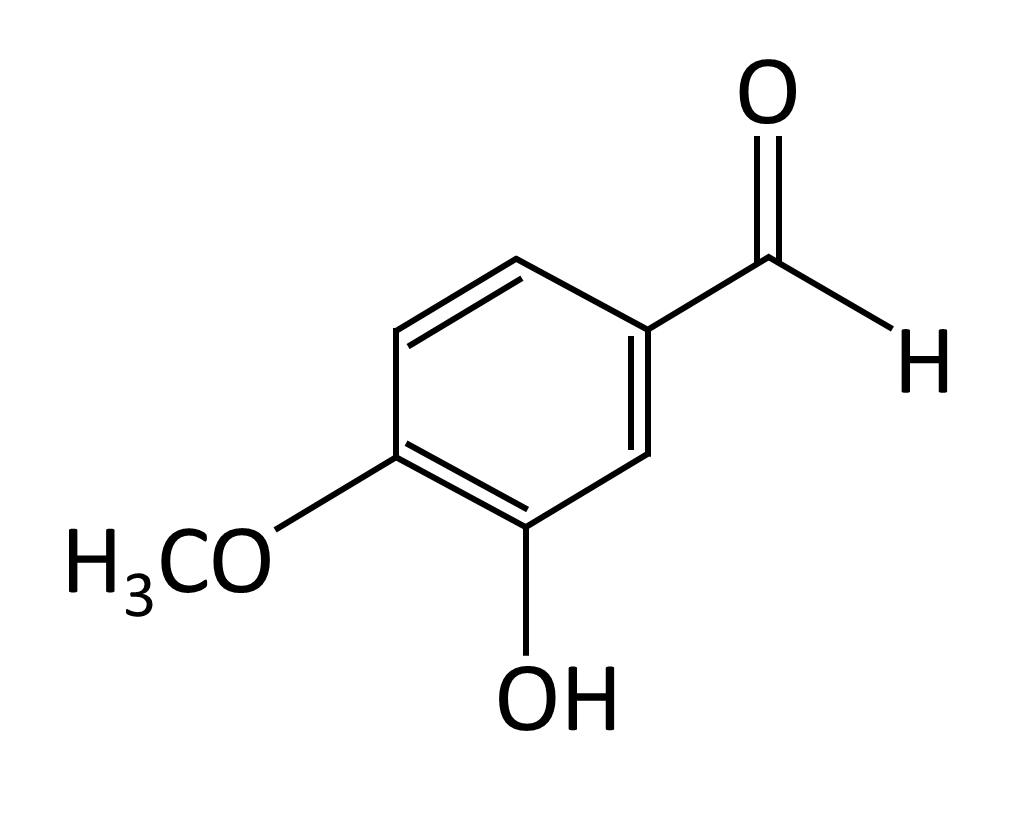 | nm | 1770 ± 153 | 135 ± 10 | nm | 342 ± 57 | 139 ± 25 | nm | 5 ± 1 | 1.1 ± 0.3 |
| **Phenylacetaldehyde**  (honey) | 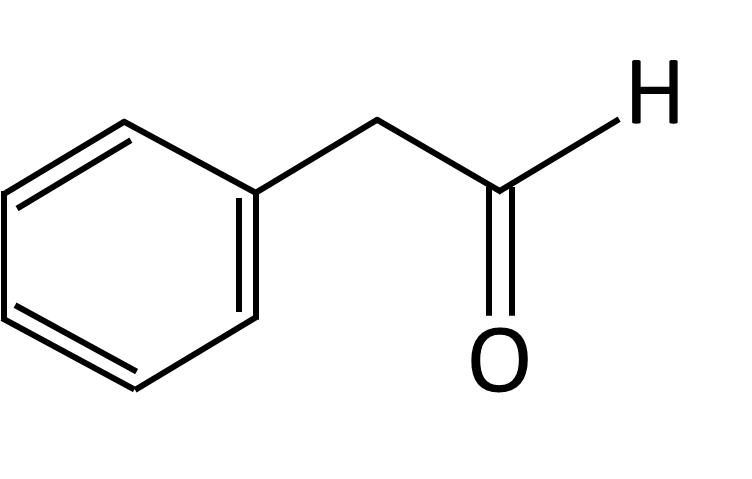 | nm | nm | 345 ± 37 | nm | nm | 245 ± 57 | nm | nm | 1.5 ± 0.4 |
| **Vanillin**  (vanilla) | 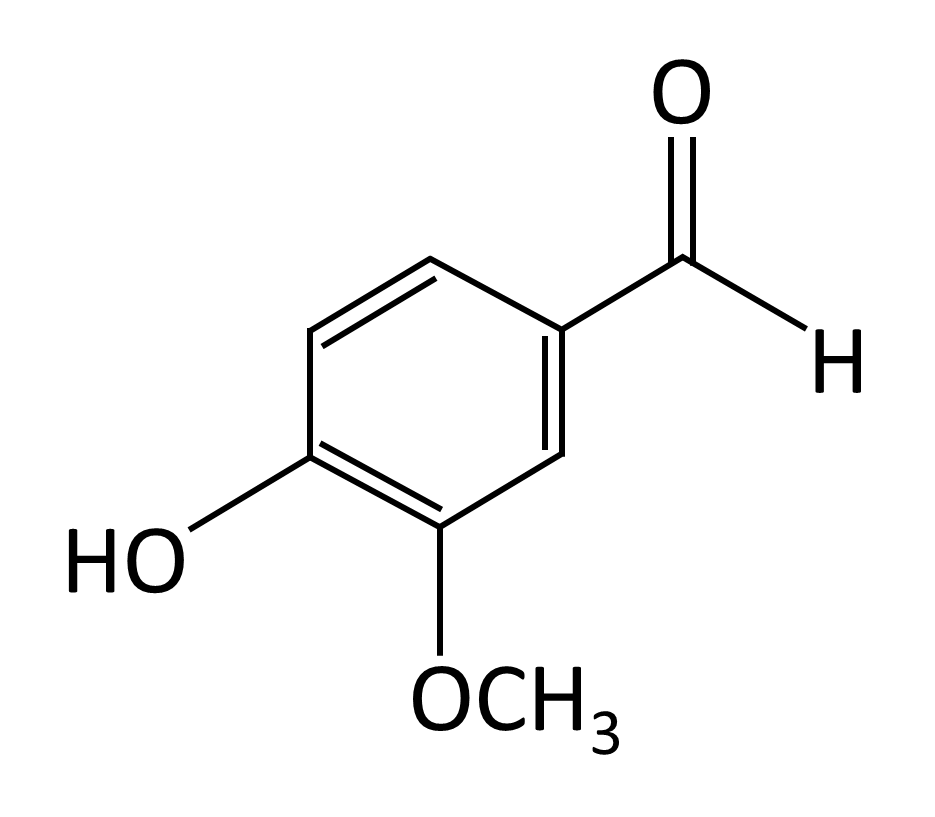 | nm | nm | 223 ± 24 | nm | nm | 6 ± 2 | nm | nm | 45 ± 15 |

**Supplemental Table 1.** **Kinetic parameters of 20 aldehydes metabolized by ALDH1A1, ALDH3A1, and AKR1B10.** Enzymatic activity was measured spectrophotometrically at 25 °C by following the absorbance of NAD(P)H at 340 nm. The initial rates were calculated using an extinction coefficient of 6220 M^-1^.cm^-1^ for NAD(P)H and fitted to the Michaelis‒Menten equation using SigmaPlot software. The catalytic parameters (k_cat_ and K_M_) were obtained for each aldehyde and enzyme with at least two replicates. Data in this table are the average of the replicates with standard deviations. nm stands for not measurable and means that the calculation of K_M_ value was impossible because the enzymatic activity increased linearly with the substrate concentration

| **Odorant common name** | **CAS number** | **Sigma-Aldrich catalog number** |
| --- | --- | --- |
| Propanal | 123-38-6 | 538124 |
| Butanal | 123-72-8 | W221902 |
| Pentanal | 110-62-3 | 110132 |
| Hexanal | 66-25-1 | W255718 |
| Heptanal | 111-71-7 | W254002 |
| Octanal | [124-13-0](https://www.sigmaaldrich.com/FR/fr/search/124-13-0?focus=products&page=1&perpage=30&sort=relevance&term=124-13-0&type=cas_number) | O5608 |
| Nonanal | 124-19-6 | W278203 |
| Decanal | 112-31-2 | D7384 |
| Undecanal | 112-44-7 | 80139 |
| Dodecanal | 112-54-9 | D222003 |
| Tridecanal | 10486-19-8 | W512702 |
| Trans-2-hexenal | 6728-26-3 | W256005 |
| Cis-4-heptenal | 6728-31-0 | W328901 |
| Trans-2-nonenal | 18829-56-6 | W321399 |
| Benzaldehyde | 100-52-7 | B1334 |
| Cinnamaldehyde | 104-55-2 | W228613 |
| Hydrocinnamaldehyde | 104-53-0 | 393193 |
| Isovanillin | 621-59-0 | 59940 |
| Phenylacetaldehyde | 122-78-1 | 107395 |
| Vanillin | 121-33-5 | V1104 |

**Supp Table 2. Odorants used for enzymatic assay.** The CAS number and the catalog number are indicated.

| Protein | [IPTG] (mM) | Growth temperature (°C) | Time growth after induction (hour) |
| --- | --- | --- | --- |
| ALDH1A1 | 1 | 37 | 24 |
| ALDH3A1 | 0.1 | 16 | 24 |
| AKR1B10 | 1 | 28 | 4 |

**Supp Table 3.** **Culture conditions for the expression of the proteins of interest.**

| Protein | Concentration in ammonium sulfate for first precipitation | Concentration in ammonium sulfate for the second precipitation | Column and resin used for the first chromatography | Column and resin used for the second chromatography |
| --- | --- | --- | --- | --- |
| ALDH1A1 | 50% | 60% | DEAE | Q-Sepharose |
| ALDH3A1 | - | - | His-Trap | Mono-Q |
| AKR1B10 | 50% | - | His-Trap | Mono-Q |

**Supp Table 4.** **Protein purification steps.** DEAE corresponds to diethylaminoethyl cellulose resin, His-Trap corresponds to high-performance immobilized metal affinity chromatography (IMAC) columns for His-tagged recombinant protein purification, and Q-Sepharose and Mono-Q are strong anion exchange resins.

| **Protein** |  | **ALDH3A1-octanal** |
| --- | --- | --- |
|  |  |  |
| **Diffraction data** | |  |
|  | Diffraction source | SOLEIL, PX1 |
|  | Detector | EIGER X-16M |
|  | Wavelength (Å) | 0.978565 |
|  | Unit-cell parameters |  |
|  | a, b, c (Å) | 61.3 85.8 169.2 |
|  | α, β, γ (°) | 90 90 90 |
|  | Space group | *P*2_1_2_1_2_1_ |
|  | Resolution range (Å) | 49.66 – 1.80 (1.83 – 1.80) |
|  | Total No. of reflections | 1035401 (24982) |
|  | No. of unique reflections | 3264 (512) |
|  | Average redundancy | 12.6 (7.5) |
|  | Mean I/σ(I) | 8.8 (1.1) |
|  | Completeness (%) | 97.6 (74.4) |
|  | R_merge_ | 0.318 (1.676) |
|  | R_meas_ | 0.331 (1.799) |
|  | R_pim_ | 0.091 (0.632) |
|  | CC_1/2_ | 0.990 (0.541) |
| **Refinement** | |  |
|  | Resolution range (Å) | 49.66 – 1.80 (1.82 – 1.80) |
|  | R_work_/R_free_ | 0.18 / 0.23 (0.29 / 0.31) |
|  | No. of protein atoms | 7001 |
|  | No. of ligand atoms | 60 |
|  | Mean B factor (Å^2^) | 26.32 |
| **Model quality** | |  |
|  | RMSZ bond lengths | 0.36 |
|  | RMSZ bond angles | 0.56 |
|  | Ramachandran favoured (%) | 97.9 |
|  | Ramachandran outliers (%) | 0.2 |
|  | Molprobity rotamer outliers (%) | 0.9 |
|  | Molprobity score | 1.04 |
| **PDB code** |  | **8BB8** |

**Supp Table 5. X-ray diffraction and refinement statistics.** CC_1/2_ is the correlation coefficient of the mean intensities between two random half-sets of data. In total, 5% of the reflections were selected for the R_free_ calculation. Values between parentheses are for the highest resolution shell.

| **Protein name** | **Gene name** | **Accession Uniprot** | **Molecular weight (g.mol^-1^)** | **Unique peptides** | **Number of spectra** | | |
| --- | --- | --- | --- | --- | --- | --- | --- |
|  |  |  |  |  | **Patient 1** | **Patient 2** | **Patient 3** |
| Serum albumin | ALB | P02768 | 69 367 | 149 | 931 | 807 | 148 |
| Retinal dehydrogenase 1 | ALDH1A1 | P00352 | 54 862 | 59 | 299 | 212 | 73 |
| Aldehyde dehydrogenase, dimeric NADP-preferring | ALDH3A1 | P30838 | 50 395 | 52 | 248 | 207 | 30 |
| Serpin B3 | SERPINB3 | P29508 | 44 565 | 55 | 165 | 184 | 63 |
| Methanethiol oxidase | SELENBP1 | Q13228 | 52 391 | 47 | 194 | 166 | 49 |
| Glutathione S-transferase P | GSTP1 | P09211 | 23 356 | 23 | 193 | 124 | 42 |
| Pyruvate kinase PKM | PKM | P14618 | 57 937 | 52 | 149 | 146 | 33 |
| Alpha-enolase | ENO1 | P06733 | 47 169 | 35 | 149 | 110 | 14 |
| Glyceraldehyde-3-phosphate dehydrogenase | GAPDH | P04406 | 36 053 | 42 | 101 | 125 | 33 |
| Triosephosphate isomerase | TPI1 | P60174 | 30 791 | 26 | 126 | 92 | 19 |
| Phosphoglycerate kinase 1 | PGK1 | P00558 | 44 615 | 40 | 131 | 96 | 9 |
| L-lactate dehydrogenase B chain | LDHB | P07195 | 36 638 | 27 | 90 | 94 | 19 |
| Polymeric immunoglobulin receptor | PIGR | P01833 | 83 284 | 39 | 52 | 115 | 28 |
| Alcohol dehydrogenase class 4 mu/sigma chain | ADH7 | P40394 | 41 481 | 35 | 100 | 77 | 14 |
| Haptoglobin | HP | P00738 | 45 205 | 28 | 83 | 84 | 19 |
| Alcohol dehydrogenase 1C | ADH1C | P00326 | 39 868 | 29 | 99 | 74 | 12 |
| Immunoglobulin heavy constant alpha 1 | IGHA1 | P01876 | 37 655 | 29 | 52 | 103 | 29 |
| Heat shock cognate 71 kDa protein | HSPA8 | P11142 | 70 898 | 41 | 73 | 91 | 17 |
| Fructose-bisphosphate aldolase A | ALDOA | P04075 | 39 420 | 31 | 84 | 71 | 18 |
| Alpha-1-antitrypsin | SERPINA1 | P01009 | 46 737 | 31 | 58 | 87 | 14 |
| Peroxiredoxin-6 | PRDX6 | P30041 | 25 035 | 18 | 80 | 62 | 7 |
| Heat shock 70 kDa protein 1A | HSPA1A | P0DMV8 | 70 052 | 35 | 61 | 70 | 18 |
| Isocitrate dehydrogenase [NADP] cytoplasmic | IDH1 | O75874 | 46 659 | 39 | 59 | 63 | 18 |
| Heat shock protein HSP 90-alpha | HSP90AA1 | P07900 | 84 660 | 35 | 48 | 85 | 3 |
| Phosphatidylethanolamine-binding protein 1 | PEBP1 | P30086 | 21 057 | 16 | 64 | 49 | 17 |
| Phosphoglycerate mutase 1 | PGAM1 | P18669 | 28 804 | 27 | 66 | 60 | 3 |
| Peroxiredoxin-5, mitochondrial | PRDX5 | P30044 | 22 086 | 16 | 55 | 53 | 14 |
| Peptidyl-prolyl cis-trans isomerase A | PPIA | P62937 | 18 012 | 21 | 62 | 56 | 3 |
| Transaldolase | TALDO1 | P37837 | 37 540 | 26 | 58 | 49 | 12 |
| 6-phosphogluconate dehydrogenase, decarboxylating | PGD | P52209 | 53 140 | 25 | 52 | 56 | 2 |
| Actin, alpha cardiac muscle 1 | ACTC1 | P68032 | 42 019 | 24 | 48 | 53 | 8 |
| Leukotriene A-4 hydrolase | LTA4H | P09960 | 69 285 | 31 | 58 | 47 | 3 |
| Glucose-6-phosphate isomerase | GPI | P06744 | 63 147 | 25 | 53 | 50 | 5 |
| Malate dehydrogenase, cytoplasmic | MDH1 | P40925 | 36 426 | 21 | 57 | 37 | 10 |
| Calcyphosin | CAPS | Q13938 | 30 240 | 14 | 54 | 45 | 4 |
| Cytosol aminopeptidase | LAP3 | P28838 | 56 166 | 28 | 51 | 39 | 13 |
| Serpin B6 | SERPINB6 | P35237 | 42 622 | 21 | 46 | 45 | 9 |
| Liver carboxylesterase 1 | CES1 | P23141 | 62 521 | 24 | 45 | 52 | 3 |
| UDP-glucose 6-dehydrogenase | UGDH | O60701 | 55 024 | 28 | 48 | 44 | 6 |
| Rab GDP dissociation inhibitor beta | GDI2 | P50395 | 50 663 | 35 | 45 | 48 | 4 |
| Cytosolic non-specific dipeptidase | CNDP2 | Q96KP4 | 52 878 | 26 | 47 | 50 |  |
| Glutathione S-transferase A2 | GSTA2 | P09210 | 25 664 | 25 | 42 | 39 | 15 |
| Aldo-keto reductase family 1 member A1 | AKR1A1 | P14550 | 36 573 | 21 | 44 | 48 | 4 |
| Carbonyl reductase [NADPH] 1 | CBR1 | P16152 | 30 375 | 18 | 50 | 41 | 4 |
| Phosphoglucomutase-1 | PGM1 | P36871 | 61 449 | 33 | 50 | 38 | 6 |
| Protein disulfide-isomerase | P4HB | P07237 | 57 116 | 26 | 41 | 51 | 1 |
| Protein/nucleic acid deglycase DJ-1 | PARK7 | Q99497 | 19 891 | 23 | 49 | 36 | 6 |
| Leukocyte elastase inhibitor | SERPINB1 | P30740 | 42 742 | 26 | 44 | 38 | 9 |
| Transgelin-2 | TAGLN2 | P37802 | 22 391 | 25 | 48 | 38 | 5 |
| Transketolase | TKT | P29401 | 67 878 | 25 | 37 | 47 | 2 |
| Immunoglobulin gamma-1 heavy chain |  | P0DOX5 | 49 329 | 21 | 27 | 38 | 20 |
| Zinc-alpha-2-glycoprotein | AZGP1 | P25311 | 34 259 | 18 | 36 | 41 | 8 |
| 4-trimethylaminobutyraldehyde dehydrogenase | ALDH9A1 | P49189 | 53 802 | 22 | 42 | 36 | 6 |
| Fatty acid-binding protein 5 | FABP5 | Q01469 | 15 164 | 20 | 43 | 34 | 5 |
| Heat shock protein HSP 90-beta | HSP90AB1 | P08238 | 83 264 | 29 | 25 | 56 | 0 |
| Endoplasmic reticulum chaperone BiP | HSPA5 | P11021 | 72 333 | 25 | 39 | 40 | 1 |
| L-lactate dehydrogenase A chain | LDHA | P00338 | 36 689 | 20 | 32 | 36 | 7 |
| Malate dehydrogenase, mitochondrial | MDH2 | P40926 | 35 503 | 18 | 33 | 39 | 2 |
| Ezrin | EZR | P15311 | 69 413 | 29 | 32 | 39 | 1 |
| Aminopeptidase B | RNPEP | Q9H4A4 | 72 596 | 28 | 30 | 42 |  |
| Immunoglobulin heavy constant gamma 2 | IGHG2 | P01859 | 35 901 | 14 | 25 | 36 | 9 |
| Tubulin beta-4A chain | TUBB4A | P04350 | 49 586 | 33 | 24 | 43 | 3 |
| Cystatin-SN | CST1 | P01037 | 16 388 | 9 | 2 | 60 | 7 |
| 6-phosphogluconolactonase | PGLS | O95336 | 27 547 | 12 | 34 | 31 | 4 |
| Hemopexin | HPX | P02790 | 51 676 | 22 | 33 | 27 | 7 |
| Arachidonate 15-lipoxygenase | ALOX15 | P16050 | 74 804 | 26 | 13 | 51 | 3 |
| Immunoglobulin kappa constant | IGKC | P01834 | 11 765 | 11 | 17 | 40 | 9 |
| Serpin B4 | SERPINB4 | P48594 | 44 854 | 39 | 26 | 34 | 5 |
| 14-3-3 protein epsilon | YWHAE | P62258 | 29 174 | 17 | 24 | 37 | 3 |
| Ribonuclease inhibitor | RNH1 | P13489 | 49 973 | 21 | 31 | 32 | 1 |
| IgGFc-binding protein | FCGBP | Q9Y6R7 | 572 017 | 39 | 7 | 56 | 1 |
| Thioredoxin reductase 1, cytoplasmic | TXNRD1 | Q16881 | 70 906 | 21 | 33 | 28 | 2 |
| N(G),N(G)-dimethylarginine dimethylaminohydrolase 1 | DDAH1 | O94760 | 31 122 | 16 | 36 | 26 |  |
| Prostaglandin reductase 1 | PTGR1 | Q14914 | 35 870 | 14 | 35 | 23 | 4 |
| Cathepsin B | CTSB | P07858 | 37 822 | 15 | 32 | 27 | 3 |
| Actin, cytoplasmic 2 | ACTG1 | P63261 | 41 793 | 39 | 22 | 28 | 11 |
| Adenosylhomocysteinase | AHCY | P23526 | 47 716 | 16 | 34 | 25 | 2 |
| Omega-amidase NIT2 | NIT2 | Q9NQR4 | 30 608 | 16 | 31 | 26 | 4 |
| Profilin-1 | PFN1 | P07737 | 15 054 | 17 | 28 | 26 | 7 |
| Ubiquitin-60S ribosomal protein L40 | UBA52 | P62987 | 14 728 | 6 | 28 | 32 | 1 |
| 14-3-3 protein zeta/delta | YWHAZ | P63104 | 27 745 | 17 | 28 | 31 | 2 |
| Annexin A1 | ANXA1 | P04083 | 38 714 | 16 | 41 | 12 | 7 |
| Rab GDP dissociation inhibitor alpha | GDI1 | P31150 | 50 583 | 30 | 27 | 32 | 1 |
| Glyoxalase domain-containing protein 4 | GLOD4 | Q9HC38 | 34 793 | 16 | 33 | 22 | 4 |
| Adenylosuccinate synthetase isozyme 2 | ADSS | P30520 | 50 097 | 17 | 26 | 32 |  |
| Aldose 1-epimerase | GALM | Q96C23 | 37 766 | 13 | 29 | 22 | 6 |
| BPI fold-containing family A member 1 | BPIFA1 | Q9NP55 | 26 713 | 12 | 13 | 29 | 15 |
| Rho GDP-dissociation inhibitor 1 | ARHGDIA | P52565 | 23 207 | 11 | 30 | 25 | 1 |
| Neutrophil gelatinase-associated lipocalin | LCN2 | P80188 | 22 588 | 11 | 22 | 25 | 9 |
| Cystatin-B | CSTB | P04080 | 11 140 | 11 | 26 | 27 | 2 |
| Prolyl endopeptidase | PREP | P48147 | 80 700 | 23 | 31 | 23 |  |
| Immunoglobulin alpha-2 heavy chain |  | P0DOX2 | 48 934 | 16 | 17 | 31 | 6 |
| Creatine kinase B-type | CKB | P12277 | 42 644 | 16 | 33 | 19 | 1 |
| Dipeptidyl peptidase 3 | DPP3 | Q9NY33 | 82 589 | 16 | 26 | 26 | 1 |
| Beta-actin-like protein 2 | ACTBL2 | Q562R1 | 42 003 | 19 | 13 | 26 | 13 |
| Phosphoglucomutase-2 | PGM2 | Q96G03 | 68 283 | 22 | 26 | 25 | 1 |
| Prolactin-inducible protein | PIP | P12273 | 16 572 | 11 | 12 | 26 | 14 |
| Adenylate kinase isoenzyme 1 | AK1 | P00568 | 21 635 | 15 | 31 | 19 |  |
| Vitamin D-binding protein | GC | P02774 | 52 918 | 16 | 19 | 31 |  |
| Alcohol dehydrogenase class-3 | ADH5 | P11766 | 39 724 | 16 | 26 | 20 | 2 |
| Aldo-keto reductase family 1 member B10 | AKR1B10 | O60218 | 36 020 | 13 | 26 | 22 |  |
| S-formylglutathione hydrolase | ESD | P10768 | 31 463 | 10 | 26 | 22 |  |
| Puromycin-sensitive aminopeptidase | NPEPPS | P55786 | 103 276 | 22 | 25 | 23 |  |
| Glutathione reductase, mitochondrial | GSR | P00390 | 56 257 | 18 | 15 | 29 | 4 |
| Plastin-2 | LCP1 | P13796 | 70 288 | 25 | 14 | 33 |  |
| Elongation factor 1-alpha 1 | EEF1A1 | P68104 | 50 141 | 12 | 23 | 23 | 1 |
| Chloride intracellular channel protein 1 | CLIC1 | O00299 | 26 923 | 13 | 21 | 22 | 4 |
| Thymidine phosphorylase | TYMP | P19971 | 49 955 | 18 | 19 | 25 | 3 |
| Alpha-1-antichymotrypsin | SERPINA3 | P01011 | 47 651 | 16 | 18 | 23 | 6 |
| Immunoglobulin lambda constant 2 | IGLC2 | P0DOY2 | 11 294 | 7 | 20 | 23 | 4 |
| Anterior gradient protein 2 homolog | AGR2 | O95994 | 19 979 | 10 | 24 | 20 | 2 |
| Cathepsin D | CTSD | P07339 | 44 552 | 13 | 25 | 18 | 2 |
| Gamma-glutamylcyclotransferase | GGCT | O75223 | 21 008 | 13 | 24 | 18 | 3 |
| Calreticulin | CALR | P27797 | 48 142 | 12 | 22 | 23 |  |
| Transcobalamin-1 | TCN1 | P20061 | 48 207 | 12 | 18 | 19 | 8 |
| Protein disulfide-isomerase A3 | PDIA3 | P30101 | 56 782 | 18 | 16 | 29 |  |
| Cofilin-1 | CFL1 | P23528 | 18 502 | 7 | 18 | 20 | 7 |
| Alpha-aminoadipic semialdehyde dehydrogenase | ALDH7A1 | P49419 | 58 487 | 15 | 24 | 20 |  |
| Glutathione synthetase | GSS | P48637 | 52 385 | 13 | 22 | 18 | 4 |
| Transthyretin | TTR | P02766 | 15 887 | 6 | 23 | 18 | 2 |
| Aldo-keto reductase family 1 member C2 | AKR1C2 | P52895 | 36 735 | 12 | 23 | 19 |  |
| Actin, cytoplasmic 1 | ACTB | P60709 | 41 737 | 39 | 15 | 19 | 7 |
| Galectin-3 | LGALS3 | P17931 | 26 152 | 7 | 21 | 18 | 2 |
| Tubulin polymerization-promoting protein family member 3 | TPPP3 | Q9BW30 | 18 985 | 13 | 19 | 17 | 5 |
| Immunoglobulin kappa light chain |  | P0DOX7 | 23 379 | 13 | 10 | 27 | 3 |
| Glyoxylate reductase/hydroxypyruvate reductase | GRHPR | Q9UBQ7 | 35 668 | 13 | 23 | 17 |  |
| Fructose-1,6-bisphosphatase 1 | FBP1 | P09467 | 36 842 | 9 | 20 | 19 | 1 |
| Glutathione S-transferase omega-1 | GSTO1 | P78417 | 27 566 | 12 | 20 | 17 | 2 |
| UMP-CMP kinase | CMPK1 | P30085 | 22 222 | 11 | 19 | 20 |  |
| Serpin B5 | SERPINB5 | P36952 | 42 100 | 14 | 27 | 10 | 1 |
| Protein ABHD14B | ABHD14B | Q96IU4 | 22 346 | 8 | 18 | 17 | 3 |
| Ketimine reductase mu-crystallin | CRYM | Q14894 | 33 776 | 13 | 17 | 17 | 4 |
| IMP cyclohydrolase | ATIC | P31939 | 64 616 | 17 | 22 | 15 |  |
| Aspartate aminotransferase, cytoplasmic | GOT1 | P17174 | 46 248 | 16 | 19 | 18 |  |
| Adenine phosphoribosyltransferase | APRT | P07741 | 19 608 | 10 | 17 | 14 | 6 |
| Sorbitol dehydrogenase | SORD | Q00796 | 38 325 | 10 | 15 | 21 | 1 |
| Sialic acid synthase | NANS | Q9NR45 | 40 308 | 14 | 13 | 24 |  |
| Alpha-1-acid glycoprotein 1 | ORM1 | P02763 | 23 512 | 6 | 21 | 13 | 3 |
| Tubulin alpha-1C chain | TUBA1C | Q9BQE3 | 49 895 | 18 | 12 | 24 |  |
| Immunoglobulin J chain | JCHAIN | P01591 | 18 099 | 7 | 11 | 23 | 2 |
| Immunoglobulin mu heavy chain |  | P0DOX6 | 63 486 | 15 | 10 | 26 |  |
| BPI fold-containing family B member 1 | BPIFB1 | Q8TDL5 | 52 442 | 15 | 4 | 27 | 5 |
| Transforming protein RhoA | RHOA | P61586 | 21 768 | 9 | 18 | 18 |  |
| Tubulin beta-4B chain | TUBB4B | P68371 | 49 831 | 38 | 12 | 22 | 1 |
| Annexin A3 | ANXA3 | P12429 | 36 375 | 18 | 23 | 4 | 8 |
| Ubiquitin-like modifier-activating enzyme 1 | UBA1 | P22314 | 117 849 | 16 | 18 | 16 | 1 |
| Lactoylglutathione lyase | GLO1 | Q04760 | 20 778 | 8 | 16 | 17 | 2 |
| Rho GTPase-activating protein 18 | ARHGAP18 | Q8N392 | 74 977 | 17 | 13 | 22 |  |
| Immunoglobulin heavy constant gamma 3 | IGHG3 | P01860 | 41 287 | 15 | 15 | 17 | 3 |
| Putative beta-actin-like protein 3 | POTEKP | Q9BYX7 | 42 016 | 7 | 18 | 16 | 1 |
| Acyl-CoA-binding protein | DBI | P07108 | 10 044 | 7 | 19 | 13 | 2 |
| Inositol monophosphatase 1 | IMPA1 | P29218 | 30 189 | 11 | 19 | 15 |  |
| Glycerol-3-phosphate dehydrogenase 1-like protein | GPD1L | Q8N335 | 38 419 | 13 | 16 | 18 |  |
| Zymogen granule protein 16 homolog B | ZG16B | Q96DA0 | 22 739 | 6 | 10 | 16 | 8 |
| 60 kDa heat shock protein, mitochondrial | HSPD1 | P10809 | 61 055 | 9 | 6 | 13 | 15 |
| Fructose-bisphosphate aldolase C | ALDOC | P09972 | 39 456 | 7 | 12 | 19 | 3 |
| D-3-phosphoglycerate dehydrogenase | PHGDH | O43175 | 56 650 | 10 | 16 | 17 |  |
| Pyridoxal kinase | PDXK | O00764 | 35 102 | 12 | 14 | 18 | 1 |
| UTP--glucose-1-phosphate uridylyltransferase | UGP2 | Q16851 | 56 940 | 11 | 11 | 22 |  |
| Aldo-keto reductase family 1 member C3 | AKR1C3 | P42330 | 36 853 | 9 | 17 | 16 |  |
| Moesin | MSN | P26038 | 67 820 | 21 | 13 | 19 | 1 |
| Adenylyl cyclase-associated protein 1 | CAP1 | Q01518 | 51 901 | 17 | 8 | 22 | 2 |
| Immunoglobulin lambda constant 7 | IGLC7 | A0M8Q6 | 11 254 | 4 | 11 | 20 | 0 |
| 3'(2'),5'-bisphosphate nucleotidase 1 | BPNT1 | O95861 | 33 392 | 11 | 16 | 15 |  |
| Na(+)/H(+) exchange regulatory cofactor NHE-RF1 | SLC9A3R1 | O14745 | 38 868 | 11 | 16 | 14 |  |
| Macrophage migration inhibitory factor | MIF | P14174 | 12 476 | 7 | 13 | 11 | 6 |
| WD repeat-containing protein 1 | WDR1 | O75083 | 66 194 | 14 | 13 | 17 |  |
| Gamma-enolase | ENO2 | P09104 | 47 269 | 11 | 15 | 13 | 1 |
| POTE ankyrin domain family member E | POTEE | Q6S8J3 | 121 363 | 14 | 13 | 15 | 1 |
| Succinyl-CoA:3-ketoacid coenzyme A transferase 1, mitochondrial | OXCT1 | P55809 | 56 158 | 12 | 15 | 14 |  |
| GTP-binding nuclear protein Ran | RAN | P62826 | 24 423 | 11 | 14 | 15 |  |
| Biliverdin reductase A | BLVRA | P53004 | 33 428 | 11 | 13 | 15 | 1 |
| Adseverin | SCIN | Q9Y6U3 | 80 489 | 15 | 9 | 20 |  |
| Glutamate--cysteine ligase catalytic subunit | GCLC | P48506 | 72 766 | 15 | 9 | 20 |  |
| Dipeptidyl peptidase 1 | CTSC | P53634 | 51 854 | 11 | 8 | 21 |  |
| Alcohol dehydrogenase 1B | ADH1B | P00325 | 39 855 | 16 | 17 | 10 | 2 |
| Inorganic pyrophosphatase | PPA1 | Q15181 | 32 660 | 11 | 16 | 12 |  |
| Queuosine salvage protein | C9orf64 | Q5T6V5 | 39 029 | 6 | 16 | 12 |  |
| ADP-sugar pyrophosphatase | NUDT5 | Q9UKK9 | 24 328 | 9 | 14 | 13 | 1 |
| Ferritin heavy chain | FTH1 | P02794 | 21 226 | 8 | 14 | 14 |  |
| Ubiquitin-fold modifier-conjugating enzyme 1 | UFC1 | Q9Y3C8 | 19 458 | 7 | 14 | 14 |  |
| 2'-deoxynucleoside 5'-phosphate N-hydrolase 1 | DNPH1 | O43598 | 19 108 | 5 | 13 | 13 | 2 |
| Farnesyl pyrophosphate synthase | FDPS | P14324 | 48 275 | 10 | 13 | 13 | 2 |
| Nebulin | NEB | P20929 | 772 914 | 9 | 10 | 15 | 3 |
| 14-3-3 protein sigma | SFN | P31947 | 27 774 | 13 | 12 | 15 |  |
| Thioredoxin domain-containing protein 17 | TXNDC17 | Q9BRA2 | 13 941 | 5 | 14 | 8 | 5 |
| 3-mercaptopyruvate sulfurtransferase | MPST | P25325 | 33 178 | 9 | 12 | 15 |  |
| F-actin-capping protein subunit beta | CAPZB | P47756 | 31 350 | 11 | 9 | 18 |  |
| Complement C3 | C3 | P01024 | 187 148 | 20 | 7 | 20 |  |
| Plastin-3 | PLS3 | P13797 | 70 811 | 17 | 13 | 13 |  |
| Plastin-1 | PLS1 | Q14651 | 70 253 | 14 | 10 | 16 |  |
| Ras-related C3 botulinum toxin substrate 1 | RAC1 | P63000 | 21 450 | 8 | 13 | 13 |  |
| Aldehyde dehydrogenase, mitochondrial | ALDH2 | P05091 | 56 381 | 16 | 11 | 15 |  |
| Phosphoglycerate kinase 2 | PGK2 | P07205 | 44 796 | 11 | 12 | 14 | 0 |
| Aldose reductase | AKR1B1 | P15121 | 35 853 | 8 | 15 | 10 | 1 |
| Sepiapterin reductase | SPR | P35270 | 28 048 | 11 | 15 | 11 |  |
| Prostaglandin reductase 2 | PTGR2 | Q8N8N7 | 38 499 | 11 | 14 | 12 |  |
| SH3 domain-binding glutamic acid-rich-like protein | SH3BGRL | O75368 | 12 774 | 9 | 14 | 9 | 3 |
| Glucose 1,6-bisphosphate synthase | PGM2L1 | Q6PCE3 | 70 442 | 12 | 12 | 14 |  |
| ATP synthase subunit beta, mitochondrial | ATP5F1B | P06576 | 56 560 | 11 | 10 | 16 |  |
| FAD-AMP lyase (cyclizing) | TKFC | Q3LXA3 | 58 947 | 16 | 9 | 17 |  |
| Protein disulfide-isomerase A4 | PDIA4 | P13667 | 72 932 | 15 | 8 | 18 |  |
| UDP-N-acetylglucosamine pyrophosphorylase | UAP1 | Q16222 | 58 769 | 12 | 9 | 17 |  |
| Immunoglobulin lambda-like polypeptide 5 | IGLL5 | B9A064 | 23 063 | 8 | 9 | 16 | 1 |
| ADP-ribosylation factor 1 | ARF1 | P84077 | 20 697 | 11 | 10 | 15 | 1 |
| Peroxiredoxin-2 | PRDX2 | P32119 | 21 892 | 8 | 11 | 13 | 2 |
| Annexin A2 | ANXA2 | P07355 | 38 604 | 12 | 16 | 7 | 2 |
| Ubiquitin-conjugating enzyme E2 N | UBE2N | P61088 | 17 138 | 8 | 14 | 11 |  |
| Cytosolic 10-formyltetrahydrofolate dehydrogenase | ALDH1L1 | O75891 | 98 829 | 3 | 13 | 11 | 1 |
| Destrin | DSTN | P60981 | 18 506 | 9 | 13 | 10 | 2 |
| Xaa-Pro aminopeptidase 1 | XPNPEP1 | Q9NQW7 | 69 918 | 13 | 11 | 14 |  |
| Beta-enolase | ENO3 | P13929 | 46 987 | 11 | 14 | 9 | 1 |
| Lysosomal alpha-glucosidase | GAA | P10253 | 105 324 | 10 | 14 | 10 |  |
| NADP-dependent malic enzyme | ME1 | P48163 | 64 150 | 10 | 12 | 12 |  |
| Argininosuccinate lyase | ASL | P04424 | 51 658 | 14 | 11 | 13 |  |
| Mannose-6-phosphate isomerase | MPI | P34949 | 46 656 | 9 | 10 | 14 |  |
| Pigment epithelium-derived factor | SERPINF1 | P36955 | 46 312 | 11 | 5 | 19 |  |
| Tryptophan--tRNA ligase, cytoplasmic | WARS | P23381 | 53 165 | 11 | 5 | 19 |  |
| Hepatoma-derived growth factor | HDGF | P51858 | 26 788 | 11 | 12 | 11 |  |
| Latexin | LXN | Q9BS40 | 25 750 | 5 | 12 | 10 | 1 |
| Purine nucleoside phosphorylase | PNP | P00491 | 32 118 | 10 | 12 | 11 |  |
| Pseudouridine-5'-phosphatase | PUDP | Q08623 | 25 249 | 9 | 10 | 13 |  |
| Histidine triad nucleotide-binding protein 1 | HINT1 | P49773 | 13 802 | 7 | 9 | 14 |  |
| Proteasome subunit alpha type-1 | PSMA1 | P25786 | 29 556 | 10 | 8 | 15 |  |
| Protein LEG1 homolog | LEG1 | Q6P5S2 | 37 926 | 10 | 7 | 13 | 3 |
| Alpha-1-acid glycoprotein 2 | ORM2 | P19652 | 23 603 | 6 | 13 | 8 | 1 |
| Putative hydrolase RBBP9 | RBBP9 | O75884 | 21 000 | 9 | 16 | 6 |  |
| Ubiquitin thioesterase OTUB1 | OTUB1 | Q96FW1 | 31 284 | 8 | 11 | 11 |  |
| Nicotinate phosphoribosyltransferase | NAPRT | Q6XQN6 | 57 578 | 10 | 10 | 12 |  |
| Serpin B13 | SERPINB13 | Q9UIV8 | 44 276 | 4 | 9 | 11 | 2 |
| ATP-dependent 6-ph | PFKP | Q01813 | 85 596 | 13 | 3 | 19 |  |
| Retinoid-inducible serine carboxypeptidase | SCPEP1 | Q9HB40 | 50 831 | 9 | 14 | 6 | 1 |
| D-dopachrome decarboxylase-like protein | DDTL | A6NHG4 | 14 195 | 5 | 11 | 10 |  |
| Programmed cell death 6-interacting protein | PDCD6IP | Q8WUM4 | 96 023 | 13 | 9 | 12 |  |
| Glutathione S-transferase Mu 2 | GSTM2 | P28161 | 25 745 | 8 | 14 | 6 | 1 |
| Tubulin beta-2A chain | TUBB2A | Q13885 | 49 907 | 33 | 7 | 13 | 0 |
| Tubulin beta-2B chain | TUBB2B | Q9BVA1 | 49 953 | 33 | 7 | 13 | 0 |
| Lumican | LUM | P51884 | 38 429 | 8 | 11 | 9 |  |
| Translationally-controlled tumor protein | TPT1 | P13693 | 19 595 | 6 | 11 | 9 |  |
| Heme-binding protein 2 | HEBP2 | Q9Y5Z4 | 22 875 | 7 | 10 | 9 | 1 |
| Quinone oxidoreductase | CRYZ | Q08257 | 35 207 | 8 | 10 | 10 |  |
| Proteasome activator complex subunit 1 | PSME1 | Q06323 | 28 723 | 9 | 8 | 12 |  |
| Ubiquitin-conjugating enzyme E2 variant 1 | UBE2V1 | Q13404 | 16 495 | 7 | 8 | 9 | 3 |
| Isocitrate dehydrogenase [NADP], mitochondrial | IDH2 | P48735 | 50 909 | 10 | 5 | 14 | 0 |
| Argininosuccinate synthase | ASS1 | P00966 | 46 530 | 8 | 13 | 6 |  |
| Beta-hexosaminidase subunit beta | HEXB | P07686 | 63 111 | 9 | 12 | 7 |  |
| Chloride intracellular channel protein 6 | CLIC6 | Q96NY7 | 73 012 | 9 | 11 | 8 |  |
| Glutathione peroxidase 1 | GPX1 | P07203 | 22 088 | 6 | 9 | 9 | 1 |
| Lambda-crystallin homolog | CRYL1 | Q9Y2S2 | 35 419 | 9 | 9 | 10 |  |
| Macrophage-capping protein | CAPG | P40121 | 38 499 | 7 | 9 | 10 |  |
| Quinone oxidoreductase PIG3 | TP53I3 | Q53FA7 | 35 536 | 8 | 9 | 10 |  |
| Elongation factor 1-gamma | EEF1G | P26641 | 50 119 | 9 | 5 | 14 |  |
| Lipocalin-15 | LCN15 | Q6UWW0 | 20 454 | 7 | 4 | 12 | 3 |
| Tubulin beta chain | TUBB | P07437 | 49 671 | 32 | 6 | 12 | 0 |
| Glycogen phosphorylase, brain form | PYGB | P11216 | 96 696 | 11 | 7 | 12 |  |
| UPF0160 protein MYG1, mitochondrial | C12orf10 | Q9HB07 | 42 449 | 9 | 11 | 7 |  |
| Cathepsin S | CTSS | P25774 | 37 496 | 6 | 10 | 8 |  |
| Alpha-N-acetylgalactosaminidase | NAGA | P17050 | 46 565 | 6 | 9 | 9 |  |
| Basal cell adhesion molecule | BCAM | P50895 | 67 405 | 10 | 9 | 9 |  |
| Ferritin light chain | FTL | P02792 | 20 020 | 5 | 9 | 9 |  |
| Heat shock protein beta-1 | HSPB1 | P04792 | 22 783 | 7 | 8 | 10 |  |
| Glutathione S-transferase theta-1 | GSTT1 | P30711 | 27 335 | 6 | 7 | 11 | - |
| Protein NDRG2 | NDRG2 | Q9UN36 | 40 798 | 4 | 7 | 6 | 5 |
| Proteasome subunit alpha type-6 | PSMA6 | P60900 | 27 399 | 9 | 6 | 11 | 1 |
| Immunoglobulin heavy constant mu | IGHM | P01871 | 49 440 | 14 | 3 | 15 |  |
| Alcohol dehydrogenase 1A | ADH1A | P07327 | 39 859 | 16 | 9 | 7 | 1 |
| Immunoglobulin heavy constant gamma 4 | IGHG4 | P01861 | 35 941 | 11 | 11 | 3 | 3 |
| Serpin B10 | SERPINB10 | P48595 | 45 403 | 4 | 11 | 5 | 1 |
| NAD(P)H-hydrate epimerase | NAXE | Q8NCW5 | 31 675 | 7 | 10 | 7 |  |
| Phytanoyl-CoA dioxygenase domain-containing protein 1 | PHYHD1 | Q5SRE7 | 32 411 | 8 | 10 | 7 |  |
| Inosine triphosphate pyrophosphatase | ITPA | Q9BY32 | 21 446 | 5 | 9 | 8 |  |
| NEDD8-activating enzyme E1 regulatory subunit | NAE1 | Q13564 | 60 246 | 10 | 9 | 8 |  |
| Ubiquitin-conjugating enzyme E2 L3 | UBE2L3 | P68036 | 17 862 | 4 | 9 | 8 |  |
| Xaa-Pro dipeptidase | PEPD | P12955 | 54 548 | 8 | 9 | 8 |  |
| Adenosine kinase | ADK | P55263 | 40 545 | 8 | 8 | 9 |  |
| Lipid-phosphate phosphatase | EPHX2 | P34913 | 62 616 | 9 | 8 | 9 |  |
| Synaptic vesicle membrane protein VAT-1 homolog | VAT1 | Q99536 | 41 920 | 8 | 8 | 7 | 2 |
| ATP synthase subunit alpha, mitochondrial | ATP5F1A | P25705 | 59 751 | 3 | 7 | 8 | 2 |
| Proteasome subunit alpha type-3 | PSMA3 | P25788 | 28 433 | 6 | 7 | 9 | 1 |
| Proteasome subunit alpha type-7 | PSMA7 | O14818 | 27 887 | 8 | 7 | 10 |  |
| Succinate--CoA ligase [ADP-forming] subunit beta, mitochondrial | SUCLA2 | Q9P2R7 | 50 317 | 6 | 7 | 10 |  |
| Afamin | AFM | P43652 | 69 069 | 9 | 6 | 11 |  |
| Proteasome activator complex subunit 2 | PSME2 | Q9UL46 | 27 402 | 7 | 6 | 11 |  |
| Stress-induced-phosphoprotein 1 | STIP1 | P31948 | 62 639 | 12 | 6 | 11 |  |
| Protein-arginine deiminase type-1 | PADI1 | Q9ULC6 | 74 666 | 1 | 5 | 3 | 9 |
| Dihydropyrimidinase | DPYS | Q14117 | 56 630 | 9 | 4 | 13 |  |
| Pro-cathepsin H | CTSH | P09668 | 37 394 | 8 | 4 | 13 |  |
| Tubulin alpha-4A chain | TUBA4A | P68366 | 49 924 | 16 | 5 | 12 |  |
| Cell division control protein 42 homolog | CDC42 | P60953 | 21 259 | 8 | 8 | 8 |  |
| Tubulin alpha-3E chain | TUBA3E | Q6PEY2 | 49 859 | 12 | 5 | 10 | 1 |
| Glutathione S-transferase Mu 3 | GSTM3 | P21266 | 26 560 | 9 | 6 | 10 |  |
| Ras-related protein Rab-6A | RAB6A | P20340 | 23 593 | 6 | 8 | 9 |  |
| UV excision repair protein RAD23 homolog B | RAD23B | P54727 | 43 171 | 7 | 8 | 8 |  |
| Calpain-1 catalytic subunit | CAPN1 | P07384 | 81 890 | 8 | 7 | 9 |  |
| Aspartate aminotransferase, mitochondrial | GOT2 | P00505 | 47 518 | 11 | 6 | 9 | 1 |
| Sodium-dependent noradrenaline transporter | SLC6A2 | P23975 | 69 332 | 1 | 5 | 6 | 5 |
| Serine/threonine-protein phosphatase 5 | PPP5C | P53041 | 56 879 | 10 | 5 | 11 |  |
| Adenylate kinase 2, mitochondrial | AK2 | P54819 | 26 478 | 8 | 4 | 12 |  |
| Putative heat shock protein HSP 90-beta 4 | HSP90AB4P | Q58FF6 | 58 264 | 7 | 8 | 7 | 1 |
| Glutathione S-transferase A3 | GSTA3 | Q16772 | 25 302 | 7 | 8 | 5 | 2 |
| Alpha-2-HS-glycoprotein | AHSG | P02765 | 39 341 | 6 | 9 | 6 |  |
| N(G),N(G)-dimethylarginine dimethylaminohydrolase 2 | DDAH2 | O95865 | 29 644 | 7 | 9 | 6 |  |
| Dihydropteridine reductase | QDPR | P09417 | 25 790 | 7 | 8 | 7 |  |
| 5'-nucleotidase domain-containing protein 1 | NT5DC1 | Q5TFE4 | 51 845 | 8 | 7 | 8 |  |
| Nuclear migration protein nudC | NUDC | Q9Y266 | 38 243 | 7 | 7 | 8 |  |
| Ubiquitin-conjugating enzyme E2 K | UBE2K | P61086 | 22 407 | 6 | 7 | 8 |  |
| Glycerol kinase | GK | P32189 | 61 245 | 7 | 6 | 9 |  |
| Peptidyl-prolyl cis-trans isomerase B | PPIB | P23284 | 23 743 | 7 | 6 | 9 |  |
| Superoxide dismutase [Mn], mitochondrial | SOD2 | P04179 | 24 750 | 5 | 6 | 9 |  |
| Translin | TSN | Q15631 | 26 183 | 5 | 6 | 9 |  |
| Nucleoside diphosphate kinase A | NME1 | P15531 | 17 149 | 6 | 5 | 10 |  |
| GDP-L-fucose synthase | TSTA3 | Q13630 | 35 893 | 6 | 5 | 10 |  |
| Hypoxanthine-guanine phosphoribosyltransferase | HPRT1 | P00492 | 24 579 | 9 | 5 | 8 | 2 |
| Mucin-5AC | MUC5AC | P98088 | 585 570 | 10 | 4 | 11 |  |
| Electron transfer flavoprotein subunit beta | ETFB | P38117 | 27 844 | 8 | 3 | 12 |  |
| NADPH:adrenodoxin oxidoreductase, mitochondrial | FDXR | P22570 | 53 837 | 11 | 3 | 12 |  |
| 14-3-3 protein gamma | YWHAG | P61981 | 28 303 | 9 | 6 | 8 |  |
| Aminoacylase-1 | ACY1 | Q03154 | 45 885 | 8 | 11 | 3 |  |
| Isoaspartyl peptidase/L-asparaginase | ASRGL1 | Q7L266 | 32 055 | 7 | 9 | 4 | 1 |
| NPC intracellular cholesterol transporter 2 | NPC2 | P61916 | 16 570 | 6 | 9 | 5 |  |
| Carboxypeptidase Q | CPQ | Q9Y646 | 51 888 | 8 | 8 | 6 |  |
| Low molecular weight phosphotyrosine protein phosphatase | ACP1 | P24666 | 18 042 | 5 | 8 | 6 |  |
| START domain-containing protein 10 | STARD10 | Q9Y365 | 33 049 | 5 | 7 | 7 |  |
| Thioredoxin-like protein 1 | TXNL1 | O43396 | 32 251 | 6 | 6 | 8 |  |
| Antileukoproteinase | SLPI | P03973 | 14 326 | 8 | 5 | 8 | 1 |
| Cystatin-C | CST3 | P01034 | 15 799 | 6 | 4 | 10 |  |
| GDP-mannose 4,6 dehydratase | GMDS | O60547 | 41 950 | 8 | 4 | 10 |  |
| Mannose-1-phosphate guanyltransferase beta | GMPPB | Q9Y5P6 | 39 834 | 6 | 4 | 10 |  |
| Proteasome subunit beta type-8 | PSMB8 | P28062 | 30 354 | 6 | 4 | 10 |  |
| Thiosulfate sulfurtransferase | TST | Q16762 | 33 429 | 6 | 4 | 10 |  |
| UDP-glucose 4-epimerase | GALE | Q14376 | 38 282 | 8 | 3 | 11 |  |
| Aldehyde dehydrogenase family 1 member A3 | ALDH1A3 | P47895 | 56 108 | 10 | 5 | 9 |  |
| Serine/threonine-protein phosphatase 2A 65 kDa regulatory subunit A alpha isoform | PPP2R1A | P30153 | 65 309 | 9 | 5 | 9 |  |
| Acid ceramidase | ASAH1 | Q13510 | 44 660 | 8 | 11 | 2 |  |
| 3-hydroxyisobutyryl-CoA hydrolase, mitochondrial | HIBCH | Q6NVY1 | 43 482 | 5 | 8 | 5 |  |
| Glycogenin-1 | GYG1 | P46976 | 39 384 | 5 | 8 | 5 |  |
| HD domain-containing protein 2 | HDDC2 | Q7Z4H3 | 23 390 | 6 | 8 | 5 |  |
| Platelet-activating factor acetylhydrolase IB subunit gamma | PAFAH1B3 | Q15102 | 25 734 | 6 | 8 | 5 |  |
| Protein-L-isoaspartate(D-aspartate) O-methyltransferase | PCMT1 | P22061 | 24 636 | 8 | 8 | 5 |  |
| Serine/threonine-protein phosphatase CPPED1 | CPPED1 | Q9BRF8 | 35 548 | 6 | 8 | 5 |  |
| Catechol O-methyltransferase | COMT | P21964 | 30 037 | 6 | 7 | 6 |  |
| Dipeptidyl peptidase 2 | DPP7 | Q9UHL4 | 54 341 | 7 | 7 | 6 |  |
| Endoplasmic reticulum resident protein 29 | ERP29 | P30040 | 28 993 | 4 | 7 | 6 |  |
| Phosphotriesterase-related protein | PTER | Q96BW5 | 39 018 | 5 | 7 | 6 |  |
| Histone H2B type 1-K | HIST1H2BK | O60814 | 13 890 | 4 | 6 | 5 | 2 |
| Kynurenine--oxoglutarate transaminase 3 | KYAT3 | Q6YP21 | 51 400 | 6 | 6 | 7 |  |
| Profilin-2 | PFN2 | P35080 | 15 046 | 6 | 6 | 7 |  |
| Coactosin-like protein | COTL1 | Q14019 | 15 945 | 7 | 5 | 7 | 1 |
| ERO1-like protein alpha | ERO1A | Q96HE7 | 54 393 | 9 | 5 | 8 |  |
| Galectin-3-binding protein | LGALS3BP | Q08380 | 65 331 | 4 | 5 | 8 |  |
| Secernin-1 | SCRN1 | Q12765 | 46 382 | 5 | 5 | 8 |  |
| Tetratricopeptide repeat protein 38 | TTC38 | Q5R3I4 | 52 787 | 7 | 5 | 8 | - |
| Thimet oligopeptidase | THOP1 | P52888 | 78 840 | 8 | 5 | 8 |  |
| Mitogen-activated protein kinase 1 | MAPK1 | P28482 | 41 390 | 9 | 4 | 9 |  |
| Immunoglobulin heavy variable 3-30 | IGHV3-30 | P01768 | 12 947 | 6 | 3 | 9 | 2 |
| Apolipoprotein A-I | APOA1 | P02647 | 30 778 | 8 | 2 | 10 | 1 |
| Deoxynucleoside triphosphate triphosphohydrolase SAMHD1 | SAMHD1 | Q9Y3Z3 | 72 201 | 9 | 2 | 11 |  |
| Kininogen-1 | KNG1 | P01042 | 71 957 | 9 | 2 | 11 |  |
| T-complex protein 1 subunit theta | CCT8 | P50990 | 59 621 | 12 |  | 13 |  |
| Tubulin beta-6 chain | TUBB6 | Q9BUF5 | 49 857 | 13 | 4 | 8 | 0 |
| Immunoglobulin lambda-1 light chain |  | P0DOX8 | 22 830 | 7 | 3 | 9 | 1 |
| SEC14-like protein 3 | SEC14L3 | Q9UDX4 | 46 048 | 8 | 11 | 2 |  |
| Heat shock 70 kDa protein 6 | HSPA6 | P17066 | 71 028 | 10 | 6 | 5 | 2 |
| Desmoplakin | DSP | P15924 | 331 774 | 6 | 8 | 4 |  |
| Plasma protease C1 inhibitor | SERPING1 | P05155 | 55 154 | 7 | 7 | 5 |  |
| SH3 domain-binding glutamic acid-rich-like protein 3 | SH3BGRL3 | Q9H299 | 10 438 | 4 | 7 | 5 |  |
| Activator of 90 kDa heat shock protein ATPase homolog 1 | AHSA1 | O95433 | 38 274 | 7 | 6 | 6 |  |
| Histamine N-methyltransferase | HNMT | P50135 | 33 295 | 6 | 6 | 6 |  |
| Proteasome subunit alpha type-2 | PSMA2 | P25787 | 25 899 | 4 | 6 | 6 |  |
| Rho GDP-dissociation inhibitor 2 | ARHGDIB | P52566 | 22 988 | 5 | 6 | 6 |  |
| Ubiquitin carboxyl-terminal hydrolase isozyme L3 | UCHL3 | P15374 | 26 183 | 5 | 6 | 5 | 1 |
| Uridine diphosphate glucose pyrophosphatase | NUDT14 | O95848 | 24 118 | 6 | 6 | 6 |  |
| 10 kDa heat shock protein, mitochondrial | HSPE1 | P61604 | 10 932 | 5 | 5 | 7 |  |
| GTP:AMP phosphotransferase AK3, mitochondrial | AK3 | Q9UIJ7 | 25 565 | 6 | 5 | 7 |  |
| Phospholysine phosphohistidine inorganic pyrophosphate phosphatase | LHPP | Q9H008 | 29 165 | 7 | 5 | 7 |  |
| Phosphomannomutase 2 | PMM2 | O15305 | 28 082 | 7 | 5 | 7 |  |
| Phosphopantothenate--cysteine ligase | PPCS | Q9HAB8 | 34 005 | 7 | 5 | 7 |  |
| Ras-related protein Rab-11A | RAB11A | P62491 | 24 394 | 6 | 5 | 7 |  |
| Secernin-2 | SCRN2 | Q96FV2 | 46 597 | 5 | 5 | 7 |  |
| Splicing factor 3B subunit 1 | SF3B1 | O75533 | 145 830 | 6 | 5 | 7 | - |
| Alpha-1B-glycoprotein | A1BG | P04217 | 54 254 | 8 | 4 | 8 |  |
| Sideroflexin-5 | SFXN5 | Q8TD22 | 37 124 | 1 | 4 | 4 | 4 |
| Poly(rC)-binding protein 1 | PCBP1 | Q15365 | 37 498 | 6 | 5 | 6 |  |
| 14-3-3 protein beta/alpha | YWHAB | P31946 | 28 082 | 10 | 5 | 6 | 0 |
| Putative inactive carboxylesterase 4 | CES1P1 | Q9UKY3 | 30 679 | 4 | 6 | 5 | 0 |
| Serine/threonine-protein kinase PAK 2 | PAK2 | Q13177 | 58 043 | 9 | 4 | 8 |  |
| Glucosamine-6-phosphate isomerase 1 | GNPDA1 | P46926 | 32 669 | 6 | 7 | 5 |  |
| Glucosamine-6-phosphate isomerase 2 | GNPDA2 | Q8TDQ7 | 31 085 | 6 | 6 | 6 |  |
| Immunoglobulin heavy variable 3-9 | IGHV3-9 | P01782 | 12 945 | 3 | 6 | 5 | 1 |
| Di-N-acetylchitobiase | CTBS | Q01459 | 43 760 | 6 | 7 | 4 |  |
| ADP-ribosylation factor-like protein 3 | ARL3 | P36405 | 20 456 | 4 | 6 | 5 |  |
| P2Y purinoceptor 4 | P2RY4 | P51582 | 40 963 | 1 | 5 | 2 | 4 |
| Calpain-2 catalytic subunit | CAPN2 | P17655 | 79 995 | 7 | 5 | 6 | - |
| Leucine-rich alpha-2-glycoprotein | LRG1 | P02750 | 38 178 | 4 | 5 | 6 |  |
| Myeloid-derived growth factor | MYDGF | Q969H8 | 18 795 | 4 | 5 | 6 |  |
| Asparagine--tRNA ligase, cytoplasmic | NARS | O43776 | 62 943 | 5 | 4 | 7 |  |
| F-actin-capping protein subunit alpha-1 | CAPZA1 | P52907 | 32 923 | 5 | 4 | 7 |  |
| Serine/threonine-protein phosphatase PP1-beta catalytic subunit | PPP1CB | P62140 | 37 187 | 6 | 4 | 7 |  |
| Glucosidase 2 subunit beta | PRKCSH | P14314 | 59 425 | 4 | 3 | 8 |  |
| Hydroxyacylglutathione hydrolase, mitochondrial | HAGH | Q16775 | 33 806 | 6 | 3 | 8 |  |
| Pirin | PIR | O00625 | 32 113 | 8 | 2 | 9 |  |
| Tryptase beta-2 | TPSB2 | P20231 | 30 515 | 5 | 1 | 10 |  |
| Heat shock-related 70 kDa protein 2 | HSPA2 | P54652 | 70 021 | 16 | 6 | 4 | 1 |
| Heat shock 70 kDa protein 1-like | HSPA1L | P34931 | 70 375 | 14 | 5 | 4 | 0 |
| Carbonyl reductase [NADPH] 3 | CBR3 | O75828 | 30 850 | 8 | 5 | 5 | 0 |
| 14-3-3 protein eta | YWHAH | Q04917 | 28 219 | 8 | 1 | 9 |  |
| Cocaine esterase | CES2 | O00748 | 61 807 | 6 | 7 | 3 |  |
| Interleukin-1 receptor antagonist protein | IL1RN | P18510 | 20 055 | 4 | 7 | 3 |  |
| 14 kDa phosphohistidine phosphatase | PHPT1 | Q9NRX4 | 13 833 | 5 | 6 | 4 |  |
| CD166 antigen | ALCAM | Q13740 | 65 102 | 5 | 6 | 4 |  |
| Haloacid dehalogenase-like hydrolase domain-containing protein 2 | HDHD2 | Q9H0R4 | 28 536 | 3 | 6 | 4 |  |
| Phenazine biosynthesis-like domain-containing protein | PBLD | P30039 | 31 785 | 5 | 6 | 4 |  |
| Probable aminopeptidase NPEPL1 | NPEPL1 | Q8NDH3 | 55 861 | 4 | 6 | 4 |  |
| Serine/threonine-protein phosphatase 2A activator | PTPA | Q15257 | 40 668 | 4 | 6 | 4 |  |
| Acetyl-CoA acetyltransferase, cytosolic | ACAT2 | Q9BWD1 | 41 351 | 6 | 5 | 4 | 1 |
| Cystatin-A | CSTA | P01040 | 11 006 | 3 | 5 | 5 |  |
| Enolase-phosphatase E1 | ENOPH1 | Q9UHY7 | 28 933 | 5 | 5 | 5 |  |
| Peptidyl-prolyl cis-trans isomerase-like 1 | PPIL1 | Q9Y3C6 | 18 237 | 5 | 5 | 5 |  |
| Alstrom syndrome protein 1 | ALMS1 | Q8TCU4 | 461 062 | 5 | 4 | 6 |  |
| Beta-hexosaminidase subunit alpha | HEXA | P06865 | 60 703 | 5 | 4 | 6 |  |
| Intraflagellar transport protein 25 homolog | HSPB11 | Q9Y547 | 16 297 | 4 | 4 | 4 | 2 |
| Protein kinase C and casein kinase substrate in neurons protein 1 | PACSIN1 | Q9BY11 | 50 966 | 2 | 4 | 5 | 1 |
| 25-hydroxycholesterol 7-alpha-hydroxylase | CYP7B1 | O75881 | 58 256 | 4 | 3 | 7 |  |
| Aflatoxin B1 aldehyde reductase member 2 | AKR7A2 | O43488 | 39 589 | 6 | 3 | 7 |  |
| BTB/POZ domain-containing protein KCTD12 | KCTD12 | Q96CX2 | 35 701 | 5 | 3 | 7 |  |
| Glutamine amidotransferase-like class 1 domain-containing protein 3B, mitochondrial | GATD3B | A0A0B4J2D5 | 28 142 | 7 | 3 | 7 |  |
| Lupus La protein | SSB | P05455 | 46 837 | 7 | 3 | 7 |  |
| Actin-related protein 2 | ACTR2 | P61160 | 44 761 | 8 | 2 | 8 |  |
| Immunoglobulin kappa variable 1-6 | IGKV1-6 | A0A0C4DH72 | 12 697 | 2 | 2 | 7 | 1 |
| Proteasome subunit beta type-5 | PSMB5 | P28074 | 28 480 | 6 | 2 | 8 |  |
| Plasminogen activator inhibitor 2 | SERPINB2 | P05120 | 46 596 | 7 |  | 10 |  |
| Ras-related protein Rab-14 | RAB14 | P61106 | 23 897 | 7 | 3 | 7 |  |
| Stromelysin-2 | MMP10 | P09238 | 54 151 | 7 | 9 | 1 |  |
| Ras-related protein Rab-1A | RAB1A | P62820 | 22 678 | 5 | 5 | 5 |  |
| Immunoglobulin kappa variable 3D-20 | IGKV3D-20 | A0A0C4DH25 | 12 515 | 4 | 4 | 5 | 1 |
| Tubulin alpha-8 chain | TUBA8 | Q9NY65 | 50 094 | 13 | 3 | 7 |  |
| Ubiquitin-conjugating enzyme E2 variant 2 | UBE2V2 | Q15819 | 16 363 | 5 | 4 | 5 | 0 |
| Annexin A7 | ANXA7 | P20073 | 52 739 | 7 | 9 | - |  |
| Ribokinase | RBKS | Q9H477 | 34 143 | 5 | 8 | 1 |  |
| N(4)-(beta-N-acetylglucosaminyl)-L-asparaginase | AGA | P20933 | 37 208 | 4 | 6 | 3 |  |
| THAP domain-containing protein 4 | THAP4 | Q8WY91 | 62 890 | 5 | 6 | 3 |  |
| Transcription factor EC | TFEC | O14948 | 38 788 | 1 | 6 | 3 |  |
| Histone H4 | HIST1H4A | P62805 | 11 367 | 4 | 5 | 4 |  |
|  | METTL26 |  | 22 578 | 3 | 5 | 4 |  |
| Myotrophin | MTPN | P58546 | 12 895 | 4 | 5 | 4 |  |
| NIF3-like protein 1 | NIF3L1 | Q9GZT8 | 41 968 | 5 | 5 | 4 |  |
| Nucleobindin-2 | NUCB2 | P80303 | 50 223 | 7 | 5 | 4 |  |
| Peptidyl-prolyl cis-trans isomerase FKBP1A | FKBP1A | P62942 | 11 951 | 2 | 5 | 4 |  |
| Platelet-activating factor acetylhydrolase IB subunit beta | PAFAH1B2 | P68402 | 25 569 | 5 | 5 | 3 | 1 |
| Protein S100-A11 | S100A11 | P31949 | 11 740 | 5 | 5 | 4 |  |
| Retinol-binding protein 1 | RBP1 | P09455 | 15 850 | 4 | 5 | 4 |  |
| 2-iminobutanoate/2-iminopropanoate deaminase | RIDA | P52758 | 14 494 | 4 | 4 | 5 |  |
| Cadherin-1 | CDH1 | P12830 | 97 456 | 3 | 4 | 5 |  |
| Echinoderm microtubule-associated protein-like 2 | EML2 | O95834 | 70 679 | 6 | 4 | 5 |  |
| Ester hydrolase C11orf54 | C11orf54 | Q9H0W9 | 35 117 | 5 | 4 | 4 | 1 |
| Flavin reductase (NADPH) | BLVRB | P30043 | 22 119 | 3 | 4 | 5 |  |
| Haloacid dehalogenase-like hydrolase domain-containing protein 3 | HDHD3 | Q9BSH5 | 28 000 | 5 | 4 | 5 |  |
| Inactive C-alpha-formylglycine-generating enzyme 2 | SUMF2 | Q8NBJ7 | 33 843 | 4 | 4 | 5 |  |
| Mesothelin | MSLN | Q13421 | 68 986 | 5 | 4 | 5 |  |
| Proteasome subunit beta type-6 | PSMB6 | P28072 | 25 358 | 4 | 4 | 5 |  |
| Zinc finger protein 646 | ZNF646 | O15015 | 200 762 | 2 | 4 | 5 |  |
| Heterogeneous nuclear ribonucleoprotein D0 | HNRNPD | Q14103 | 38 434 | 6 | 3 | 6 |  |
| m7GpppX diphosphatase | DCPS | Q96C86 | 38 609 | 5 | 3 | 6 |  |
| Prostaglandin E synthase 3 | PTGES3 | Q15185 | 18 697 | 4 | 3 | 6 |  |
| Proteasome subunit alpha type-4 | PSMA4 | P25789 | 29 484 | 5 | 3 | 6 |  |
| Proteasome subunit alpha type-5 | PSMA5 | P28066 | 26 411 | 5 | 3 | 6 |  |
| Proteasome subunit beta type-1 | PSMB1 | P20618 | 26 489 | 5 | 3 | 6 |  |
| Serpin B12 | SERPINB12 | Q96P63 | 46 276 | 1 | 3 | 3 | 3 |
| Beta-2-glycoprotein 1 | APOH | P02749 | 38 298 | 6 | 2 | 7 |  |
| Electron transfer flavoprotein subunit alpha, mitochondrial | ETFA | P13804 | 35 080 | 6 | 2 | 7 |  |
| Immunoglobulin heavy variable 4-61 | IGHV4-61 | A0A0C4DH41 | 13 066 | 5 | 2 | 7 |  |
| Serine--tRNA ligase, cytoplasmic | SARS | P49591 | 58 777 | 8 | 1 | 8 |  |
| Succinate--CoA ligase [GDP-forming] subunit beta, mitochondrial | SUCLG2 | Q96I99 | 46 511 | 7 | 1 | 8 |  |
| AP-2 complex subunit beta | AP2B1 | P63010 | 104 553 | 6 | 1 | 7 | 1 |
| 14-3-3 protein theta | YWHAQ | P27348 | 27 764 | 9 | 3 | 6 |  |
| Endoplasmin | HSP90B1 | P14625 | 92 469 | 8 | 2 | 7 |  |
| Serine/threonine-protein kinase PAK 1 | PAK1 | Q13153 | 60 647 | 6 | 5 | 3 |  |
| Immunoglobulin kappa variable 3-20 | IGKV3-20 | P01619 | 12 557 | 4 | 3 | 4 | 1 |
| AP-1 complex subunit beta-1 | AP1B1 | Q10567 | 104 637 | 7 | 5 | 3 |  |
| Alcohol dehydrogenase 6 | ADH6 | P28332 | 39 073 | 4 | 4 | 4 | 0 |
| NSFL1 cofactor p47 | NSFL1C | Q9UNZ2 | 40 573 | 6 | 6 | 2 |  |
| PDZ and LIM domain protein 1 | PDLIM1 | O00151 | 36 072 | 5 | 6 | 2 |  |
| Adapter molecule crk | CRK | P46108 | 33 831 | 4 | 5 | 3 |  |
| Eukaryotic translation initiation factor 6 | EIF6 | P56537 | 26 599 | 3 | 5 | 3 |  |
| Galectin-1 | LGALS1 | P09382 | 14 716 | 4 | 5 | 3 |  |
| N-acetylglucosamine-6-sulfatase | GNS | P15586 | 62 082 | 6 | 5 | 3 |  |
| NADH-cytochrome b5 reductase 2 | CYB5R2 | Q6BCY4 | 31 458 | 5 | 5 | 3 |  |
| Actin-related protein 2/3 complex subunit 2 | ARPC2 | O15144 | 34 333 | 4 | 4 | 4 |  |
| Ceruloplasmin | CP | P00450 | 122 205 | 6 | 4 | 4 |  |
| Enoyl-CoA delta isomerase 1, mitochondrial | ECI1 | P42126 | 32 816 | 4 | 4 | 4 |  |
| Eukaryotic initiation factor 4A-I | EIF4A1 | P60842 | 46 154 | 3 | 4 | 4 |  |
| Heterogeneous nuclear ribonucleoprotein K | HNRNPK | P61978 | 50 976 | 4 | 4 | 4 |  |
| Immunoglobulin heavy variable 3-64D | IGHV3-64D | A0A0J9YX35 | 12 823 | 3 | 4 | 4 |  |
| Inorganic pyrophosphatase 2, mitochondrial | PPA2 | Q9H2U2 | 37 920 | 3 | 4 | 4 |  |
| NEDD8-activating enzyme E1 catalytic subunit | UBA3 | Q8TBC4 | 51 852 | 3 | 4 | 4 |  |
| Phosphoserine phosphatase | PSPH | P78330 | 25 008 | 3 | 4 | 4 |  |
| ADP-ribose glycohydrolase ARH3 | ADPRHL2 | Q9NX46 | 38 947 | 5 | 4 | 4 |  |
| Protein S100-A8 | S100A8 | P05109 | 10 835 | 3 | 4 | 4 |  |
| Rho-related GTP-binding protein RhoF | RHOF | Q9HBH0 | 23 625 | 2 | 4 | 4 |  |
| S-methyl-5'-thioadenosine phosphorylase | MTAP | Q13126 | 31 236 | 6 | 4 | 4 |  |
| Spermine synthase | SMS | P52788 | 41 268 | 4 | 4 | 4 |  |
| 3-hydroxyisobutyrate dehydrogenase, mitochondrial | HIBADH | P31937 | 35 329 | 5 | 3 | 5 |  |
| Fumarate hydratase, mitochondrial | FH | P07954 | 54 637 | 3 | 3 | 5 |  |
| Ras-related protein Rab-2A | RAB2A | P61019 | 23 546 | 5 | 3 | 5 |  |
| Ras-related protein Rab-7a | RAB7A | P51149 | 23 490 | 4 | 3 | 5 |  |
| S-phase kinase-associated protein 1 | SKP1 | P63208 | 18 658 | 5 | 3 | 5 |  |
| Phosphoribosylglycinamide formyltransferase | GART | P22102 | 107 767 | 6 | 3 | 5 |  |
| Trinucleotide repeat-containing gene 6B protein | TNRC6B | Q9UPQ9 | 194 002 | 1 | 3 | 4 | 1 |
| Ubiquitin carboxyl-terminal hydrolase 15 | USP15 | Q9Y4E8 | 112 419 | 3 | 3 | 5 |  |
| Immunoglobulin kappa variable 4-1 | IGKV4-1 | P06312 | 13 380 | 3 | 2 | 5 | 1 |
| Nicotinamide phosphoribosyltransferase | NAMPT | P43490 | 55 521 | 5 | 2 | 6 |  |
| Perilipin-3 | PLIN3 | O60664 | 47 075 | 5 | 2 | 6 |  |
| Phosphoacetylglucosamine mutase | PGM3 | O95394 | 59 852 | 6 | 2 | 6 |  |
| S-adenosylmethionine synthase isoform type-2 | MAT2A | P31153 | 43 661 | 5 | 2 | 6 |  |
| Non-secretory ribonuclease | RNASE2 | P10153 | 18 354 | 4 |  | 8 |  |
| Ras-related protein Rab-35 | RAB35 | Q15286 | 23 025 | 2 | 5 | 2 |  |
| Glutamine--fructose-6-phosphate aminotransferase [isomerizing] 1 | GFPT1 | Q06210 | 78 806 | 7 | 1 | 7 |  |
| Poly(rC)-binding protein 3 | PCBP3 | P57721 | 39 465 | 7 | 3 | 4 |  |
| Glutamine--fructose-6-phosphate aminotransferase [isomerizing] 2 | GFPT2 | O94808 | 76 931 | 5 | 2 | 5 |  |
| Hsc70-interacting protein | ST13 | P50502 | 41 332 | 3 | 3 | 4 |  |
| Rho-related GTP-binding protein RhoC | RHOC | P08134 | 22 006 | 7 | 3 | 4 |  |
| Serpin B8 | SERPINB8 | P50452 | 42 767 | 5 | 3 | 4 | 0 |
| Putative heat shock protein HSP 90-alpha A4 | HSP90AA4P | Q58FG1 | 47 712 | 4 | 2 | 3 | 2 |
| Isochorismatase domain-containing protein 1 | ISOC1 | Q96CN7 | 32 237 | 3 | 5 | 2 |  |
| Kinesin-like protein KIF21A | KIF21A | Q7Z4S6 | 187 179 | 4 | 5 | 2 |  |
| SH3 domain-binding glutamic acid-rich protein | SH3BGR | P55822 | 26 086 | 2 | 5 | 2 |  |
| Putative nucleoside diphosphate kinase | NME2P1 | O60361 | 15 529 | 7 | 5 | 2 |  |
| Acyl-protein thioesterase 1 | LYPLA1 | O75608 | 24 670 | 2 | 4 | 3 |  |
| Calcyclin-binding protein | CACYBP | Q9HB71 | 26 210 | 4 | 4 | 3 |  |
| DNA-(apurinic or apyrimidinic site) lyase | APEX1 | P27695 | 35 555 | 6 | 4 | 3 |  |
| Maleylacetoacetate isomerase | GSTZ1 | O43708 | 24 212 | 3 | 4 | 3 |  |
| Proteasome assembly chaperone 3 | PSMG3 | Q9BT73 | 13 104 | 2 | 4 | 3 |  |
| Ras-related protein Rab-21 | RAB21 | Q9UL25 | 24 348 | 3 | 4 | 3 |  |
| Ras-related protein Rab-33B | RAB33B | Q9H082 | 25 718 | 3 | 4 | 3 |  |
| Xylulose kinase | XYLB | O75191 | 58 382 | 4 | 4 | 3 |  |
| 2,4-dienoyl-CoA reductase, mitochondrial | DECR1 | Q16698 | 36 068 | 5 | 3 | 4 |  |
| Cathepsin Z | CTSZ | Q9UBR2 | 33 868 | 3 | 3 | 4 |  |
| Delta-aminolevulinic acid dehydratase | ALAD | P13716 | 36 295 | 4 | 3 | 4 |  |
| Eukaryotic translation initiation factor 5A-1 | EIF5A | P63241 | 16 832 | 2 | 3 | 2 | 2 |
| Glutathione S-transferase kappa 1 | GSTK1 | Q9Y2Q3 | 25 497 | 4 | 3 | 4 |  |
| Interleukin-18 | IL18 | Q14116 | 22 326 | 3 | 3 | 4 |  |
| Methionine adenosyltransferase 2 subunit beta | MAT2B | Q9NZL9 | 37 552 | 5 | 3 | 4 |  |
| Mth938 domain-containing protein | AAMDC | Q9H7C9 | 13 332 | 3 | 3 | 4 |  |
| NudC domain-containing protein 2 | NUDCD2 | Q8WVJ2 | 17 676 | 3 | 3 | 4 |  |
| Persulfide dioxygenase ETHE1, mitochondrial | ETHE1 | O95571 | 27 873 | 4 | 3 | 4 |  |
| Protein FAM49B | FAM49B | Q9NUQ9 | 36 748 | 4 | 3 | 4 |  |
| 60 kDa SS-A/Ro ribonucleoprotein | TROVE2 | P10155 | 60 671 | 5 | 2 | 5 |  |
| Adenylosuccinate lyase | ADSL | P30566 | 54 889 | 5 | 2 | 5 |  |
| Diphosphoinositol polyphosphate phosphohydrolase 1 | NUDT3 | O95989 | 19 471 | 4 | 2 | 5 |  |
| Peptidyl-prolyl cis-trans isomerase FKBP4 | FKBP4 | Q02790 | 51 805 | 5 | 2 | 5 |  |
| Phosphomevalonate kinase | PMVK | Q15126 | 21 995 | 5 | 2 | 5 |  |
| Proteasome subunit beta type-4 | PSMB4 | P28070 | 29 204 | 4 | 2 | 5 |  |
| von Willebrand factor A domain-containing protein 5A | VWA5A | O00534 | 86 489 | 5 | 2 | 5 |  |
| Alpha-2-macroglobulin | A2M | P01023 | 163 291 | 5 | 1 | 6 |  |
| Fibrinogen gamma chain | FGG | P02679 | 51 512 | 5 | 1 | 6 |  |
| Glycine--tRNA ligase | GARS | P41250 | 83 166 | 5 | 1 | 6 |  |
| Succinate--CoA ligase [ADP/GDP-forming] subunit alpha, mitochondrial | SUCLG1 | P53597 | 36 250 | 4 | 1 | 6 |  |
| Histone H2A type 1-B/E | HIST1H2AB | P04908 | 14 135 | 3 | 3 | 4 | 1 |
| Haptoglobin-related protein | HPR | P00739 | 39 030 | 12 | 2 | 4 | 0 |
| Serine/threonine-protein phosphatase 2A 65 kDa regulatory subunit A beta isoform | PPP2R1B | P30154 | 66 214 | 4 | 3 | 3 |  |
| Cysteine-rich secretory protein 3 | CRISP3 | P54108 | 27 630 | 4 | 3 | 4 |  |
| EF-hand domain-containing protein D2 | EFHD2 | Q96C19 | 26 697 | 3 | 3 | 4 |  |
| Putative heat shock protein HSP 90-alpha A5 | HSP90AA5P | Q58FG0 | 38 738 | 4 | 2 | 4 |  |
| SEC14-like protein 2 | SEC14L2 | O76054 | 46 145 | 4 | 2 | 4 |  |
| Anterior gradient protein 3 | AGR3 | Q8TD06 | 19 171 | 5 | 4 | 2 |  |
| Neurofilament heavy polypeptide | NEFH | P12036 | 112 479 | 5 | 4 | 2 |  |
| Methylthioribose-1-phosphate isomerase | MRI1 | Q9BV20 | 39 150 | 6 | 6 | - |  |
| Guanine deaminase | GDA | Q9Y2T3 | 51 003 | 4 | 5 | 1 |  |
| Actin-related protein 2/3 complex subunit 1B | ARPC1B | O15143 | 40 950 | 3 | 4 | 2 |  |
| Cell | CADM4 | Q8NFZ8 | 42 785 | 4 | 4 | 2 |  |
| Dermcidin | DCD | P81605 | 11 284 | 2 | 4 | 2 |  |
| Elafin | PI3 | P19957 | 12 270 | 4 | 4 | 2 |  |
| Intraflagellar transport protein 27 homolog | IFT27 | Q9BW83 | 20 480 | 4 | 4 | 2 |  |
| LIM and SH3 domain protein 1 | LASP1 | Q14847 | 29 717 | 3 | 4 | 2 |  |
| Mammalian ependymin-related protein 1 | EPDR1 | Q9UM22 | 25 437 | 2 | 4 | 2 |  |
| Protein phosphatase 1 regulatory subunit 7 | PPP1R7 | Q15435 | 41 564 | 3 | 4 | 2 |  |
| Tyrosine-protein phosphatase non-receptor type 3 | PTPN3 | P26045 | 103 990 | 1 | 4 | 2 |  |
| Ras-related protein Rab-15 | RAB15 | P59190 | 24 391 | 2 | 3 | 3 |  |
| Actin-related protein 2/3 complex subunit 4 | ARPC4 | P59998 | 19 667 | 3 | 3 | 3 |  |
| Calpain small subunit 1 | CAPNS1 | P04632 | 28 316 | 4 | 3 | 3 |  |
| Fructose-2,6-bisphosphatase TIGAR | TIGAR | Q9NQ88 | 30 063 | 4 | 3 | 3 |  |
| Glycerol-3-phosphate phosphatase | PGP | A6NDG6 | 34 006 | 5 | 3 | 3 |  |
| GTP-binding protein SAR1a | SAR1A | Q9NR31 | 22 367 | 2 | 3 | 3 |  |
| Guanylate kinase | GUK1 | Q16774 | 21 726 | 4 | 3 | 3 |  |
| Heme-binding protein 1 | HEBP1 | Q9NRV9 | 21 097 | 3 | 3 | 3 |  |
| Immunoglobulin kappa variable 3-15 | IGKV3-15 | P01624 | 12 496 | 2 | 3 | 3 |  |
| Importin subunit beta-1 | KPNB1 | Q14974 | 97 170 | 4 | 3 | 3 |  |
| Kunitz-type protease inhibitor 1 | SPINT1 | O43278 | 58 398 | 3 | 3 | 3 |  |
| Nascent polypeptide-associated complex subunit alpha, muscle-specific form | NACA | E9PAV3 | 205 422 | 3 | 3 | 3 |  |
| Neutral alpha-glucosidase AB | GANAB | Q14697 | 106 874 | 5 | 3 | 3 |  |
| NHL repeat-containing protein 2 | NHLRC2 | Q8NBF2 | 79 444 | 4 | 3 | 3 |  |
| Paraneoplastic antigen Ma3 | PNMA3 | Q9UL41 | 52 376 | 2 | 3 | 3 |  |
| Protein SGT1 homolog | SUGT1 | Q9Y2Z0 | 41 024 | 3 | 3 | 3 |  |
| Ras-related protein Rab-28 | RAB28 | P51157 | 24 841 | 1 | 2 | 4 |  |
| 3-hydroxybutyrate dehydrogenase type 2 | BDH2 | Q9BUT1 | 26 724 | 4 | 2 | 4 |  |
| Baculoviral IAP repeat-containing protein 1 | NAIP | Q13075 | 159 582 | 1 | 2 | 2 | 2 |
| Brain acid soluble protein 1 | BASP1 | P80723 | 22 693 | 4 | 2 | 4 |  |
| Calumenin | CALU | O43852 | 37 107 | 2 | 2 | 4 |  |
| Clusterin | CLU | P10909 | 52 495 | 3 | 2 | 4 |  |
| F-box only protein 2 | FBXO2 | Q9UK22 | 33 328 | 3 | 2 | 4 |  |
| Fumarylacetoacetase | FAH | P16930 | 46 374 | 5 | 2 | 4 |  |
| Golgi-associated PDZ and coiled-coil motif-containing protein | GOPC | Q9HD26 | 50 520 | 2 | 2 | 4 | - |
| Heat shock protein 75 kDa, mitochondrial | TRAP1 | Q12931 | 80 110 | 1 | 2 | 4 |  |
| Histidine triad nucleotide-binding protein 2, mitochondrial | HINT2 | Q9BX68 | 17 162 | 3 | 2 | 4 |  |
| Immunoglobulin kappa variable 2-28 | IGKV2-28 | A0A075B6P5 | 12 957 | 2 | 2 | 4 |  |
| L-lactate dehydrogenase A-like 6B | LDHAL6B | Q9BYZ2 | 41 943 | 1 | 2 | 2 | 2 |
| Myeloperoxidase | MPO | P05164 | 83 869 | 5 | 2 | 4 |  |
| NADH-cytochrome b5 reductase 3 | CYB5R3 | P00387 | 34 235 | 4 | 2 | 4 |  |
| Obg-like ATPase 1 | OLA1 | Q9NTK5 | 44 744 | 3 | 2 | 4 |  |
| Proteasome subunit beta type-9 | PSMB9 | P28065 | 23 264 | 4 | 2 | 4 |  |
| Protein-glutamate O-methyltransferase | ARMT1 | Q9H993 | 51 172 | 3 | 2 | 4 |  |
| T-complex protein 1 subunit delta | CCT4 | P50991 | 57 924 | 3 | 2 | 4 |  |
| Translin-associated protein X | TSNAX | Q99598 | 33 112 | 3 | 2 | 4 |  |
| Tubulin-specific chaperone A | TBCA | O75347 | 12 855 | 3 | 2 | 4 |  |
| WAP four-disulfide core domain protein 2 | WFDC2 | Q14508 | 12 993 | 3 | 2 | 4 |  |
| BPI fold-containing family B member 2 | BPIFB2 | Q8N4F0 | 49 172 | 3 | 1 | 4 | 1 |
| Complement factor B | CFB | P00751 | 85 533 | 5 | 1 | 5 |  |
| Hydroxymethylglutaryl-CoA synthase, mitochondrial | HMGCS2 | P54868 | 56 635 | 5 | 1 | 5 |  |
| Lysine--tRNA ligase | KARS | Q15046 | 68 048 | 4 | 1 | 5 |  |
| Metalloproteinase inhibitor 1 | TIMP1 | P01033 | 23 171 | 4 | 1 | 5 |  |
| Reticulocalbin-1 | RCN1 | Q15293 | 38 890 | 4 | 1 | 5 |  |
| Glucose-6-phosphate 1-dehydrogenase | G6PD | P11413 | 59 257 | 5 | - | 6 |  |
| Coronin-1A | CORO1A | P31146 | 51 026 | 6 |  | 6 |  |
| Galectin-10 | CLC | Q05315 | 16 453 | 2 |  | 4 | 2 |
| Proliferation-associated protein 2G4 | PA2G4 | Q9UQ80 | 43 787 | 6 |  | 6 |  |
| Small ubiquitin-related modifier 2 | SUMO2 | P61956 | 10 871 | 2 | 2 | 3 |  |
| Glutathione S-transferase Mu 4 | GSTM4 | Q03013 | 25 561 | 6 | 4 | 1 | 0 |
| Serine hydroxymethyltransferase, cytosolic | SHMT1 | P34896 | 53 083 | 5 | 5 |  |  |
| Twinfilin-1 | TWF1 | Q12792 | 40 283 | 3 | 5 |  |  |
| Ribonuclease 4 | RNASE4 | P34096 | 16 840 | 4 | 4 | 1 |  |
| Astrocytic phosphoprotein PEA-15 | PEA15 | Q15121 | 15 040 | 2 | 3 | 2 |  |
| Bis(5'-adenosyl)-triphosphatase ENPP4 | ENPP4 | Q9Y6X5 | 51 641 | 2 | 3 | 2 |  |
| Bisphosphoglycerate mutase | BPGM | P07738 | 30 005 | 2 | 3 | 2 |  |
| Cellular retinoic acid-binding protein 2 | CRABP2 | P29373 | 15 693 | 3 | 3 | 2 |  |
| Glucosamine 6-phosphate N-acetyltransferase | GNPNAT1 | Q96EK6 | 20 749 | 2 | 3 | 2 |  |
| Glycolipid transfer protein | GLTP | Q9NZD2 | 23 850 | 4 | 3 | 2 |  |
| Phospholipid hydroperoxide glutathione peroxidase | GPX4 | P36969 | 22 175 | 4 | 3 | 2 |  |
| Platelet-activating factor acetylhydrolase IB subunit alpha | PAFAH1B1 | P43034 | 46 638 | 3 | 3 | 2 |  |
| Protein S100-A9 | S100A9 | P06702 | 13 242 | 2 | 3 | 2 |  |
| Sorcin | SRI | P30626 | 21 676 | 4 | 3 | 2 |  |
| Acetyl-CoA acetyltransferase, mitochondrial | ACAT1 | P24752 | 45 200 | 3 | 2 | 3 |  |
| Cytosolic 5'-nucleotidase 3A | NT5C3A | Q9H0P0 | 37 948 | 3 | 2 | 3 |  |
| Eukaryotic translation initiation factor 4E | EIF4E | P06730 | 25 097 | 2 | 2 | 3 |  |
| Gastrotropin | FABP6 | P51161 | 14 371 | 3 | 2 | 3 |  |
| Heat shock 70 kDa protein 4 | HSPA4 | P34932 | 94 331 | 3 | 2 | 3 |  |
| Immunoglobulin kappa variable 1D-33 | IGKV1D-33 | P01593 | 12 848 | 1 | 2 | 3 |  |
| Kallikrein-11 | KLK11 | Q9UBX7 | 31 059 | 3 | 2 | 3 |  |
| Lipoyltransferase 1, mitochondrial | LIPT1 | Q9Y234 | 42 479 | 1 | 2 | 3 |  |
| Non-specific lipid-transfer protein | SCP2 | P22307 | 58 994 | 3 | 2 | 3 |  |
| Ras-related protein Rab-5C | RAB5C | P51148 | 23 483 | 2 | 2 | 3 |  |
| RuvB-like 2 | RUVBL2 | Q9Y230 | 51 157 | 3 | 2 | 3 |  |
| Serine/threonine-protein phosphatase 2A 55 kDa regulatory subunit B alpha isoform | PPP2R2A | P63151 | 51 692 | 2 | 2 | 3 |  |
| Tubulin-folding cofactor B | TBCB | Q99426 | 27 326 | 4 | 2 | 3 |  |
| Ubiquitin carboxyl-terminal hydrolase 14 | USP14 | P54578 | 56 069 | 2 | 2 | 3 |  |
| 26S proteasome non-ATPase regulatory subunit 4 | PSMD4 | P55036 | 40 737 | 4 | 1 | 4 |  |
| Acyl-coenzyme A thioesterase 1 | ACOT1 | Q86TX2 | 46 277 | 4 | 1 | 4 |  |
| Angiotensinogen | AGT | P01019 | 53 154 | 3 | 1 | 3 | 1 |
| CD44 antigen | CD44 | P16070 | 81 538 | 2 | 1 | 4 |  |
| Coronin-1B | CORO1B | Q9BR76 | 54 235 | 4 | 1 | 4 |  |
| Dihydrolipoyl dehydrogenase, mitochondrial | DLD | P09622 | 54 177 | 4 | 1 | 4 |  |
| Elongation factor 2 | EEF2 | P13639 | 95 338 | 5 | 1 | 4 |  |
| Enoyl-CoA hydratase, mitochondrial | ECHS1 | P30084 | 31 387 | 4 | 1 | 4 |  |
| Galactokinase | GALK1 | P51570 | 42 272 | 4 | 1 | 4 |  |
| Mannose-1-phosphate guanyltransferase alpha | GMPPA | Q96IJ6 | 46 291 | 4 | 1 | 4 |  |
| Medium-chain specific acyl-CoA dehydrogenase, mitochondrial | ACADM | P11310 | 46 588 | 4 | 1 | 4 |  |
| Mitogen-activated protein kinase 15 | MAPK15 | Q8TD08 | 59 832 | 3 | 1 | 4 |  |
| Nectin-4 | NECTIN4 | Q96NY8 | 55 454 | 3 | 1 | 3 | 1 |
| Protein AMBP | AMBP | P02760 | 38 999 | 3 | 1 | 4 |  |
| Protein NOXP20 | FAM114A1 | Q8IWE2 | 60 742 | 3 | 1 | 4 |  |
| Ras-related protein R-Ras2 | RRAS2 | P62070 | 23 400 | 3 | 1 | 4 |  |
| Sialate O-acetylesterase | SIAE | Q9HAT2 | 58 315 | 4 | 1 | 4 |  |
| T-complex protein 1 subunit gamma | CCT3 | P49368 | 60 534 | 3 | 1 | 4 |  |
| Tyrosine--tRNA ligase, cytoplasmic | YARS | P54577 | 59 143 | 4 | 1 | 4 |  |
| Vitelline membrane outer layer protein 1 homolog | VMO1 | Q7Z5L0 | 21 534 | 4 | 1 | 4 |  |
| Protein-glutamine gamma-glutamyltransferase 2 | TGM2 | P21980 | 77 329 | 4 | - | 5 |  |
| Poly(rC)-binding protein 2 | PCBP2 | Q15366 | 38 580 | 6 | 2 | 2 |  |
| Ras-related protein Rab-39A | RAB39A | Q14964 | 25 007 | 4 | 1 | 3 |  |
| Cystatin-SA | CST2 | P09228 | 16 445 | 5 |  | 5 |  |
| Ubiquitin-conjugating enzyme E2 D3 | UBE2D3 | P61077 | 16 687 | 3 | 3 | 2 |  |
| Fructose-1,6-bisphosphatase isozyme 2 | FBP2 | O00757 | 36 743 | 3 | 2 | 2 |  |
| ATP-dependent RNA helicase DDX39A | DDX39A | O00148 | 49 130 | 4 | 2 | 3 |  |
| cAMP-dependent protein kinase type II-alpha regulatory subunit | PRKAR2A | P13861 | 45 518 | 4 | 2 | 3 |  |
| Sulfotransferase 1A1 | SULT1A1 | P50225 | 34 165 | 3 | 2 | 3 |  |
| Spliceosome RNA helicase DDX39B | DDX39B | Q13838 | 48 991 | 4 | 1 | 4 |  |
| ATP-dependent 6-phosphofructokinase, liver type | PFKL | P17858 | 85 018 | 3 | 2 | 2 |  |
| Histone H2A type 2-C | HIST2H2AC | Q16777 | 13 988 | 2 | 2 | 1 | 0 |
| Endoribonuclease LACTB2 | LACTB2 | Q53H82 | 32 806 | 3 | 3 | 1 |  |
| Fascin | FSCN1 | Q16658 | 54 530 | 3 | 3 | 1 |  |
| 1,2-dihydroxy-3-keto-5-methylthiopentene dioxygenase | ADI1 | Q9BV57 | 21 498 | 2 | 2 | 2 |  |
| Apolipoprotein D | APOD | P05090 | 21 276 | 2 | 2 | 2 |  |
| Apoptosis-inducing factor 2 | AIFM2 | Q9BRQ8 | 40 527 | 1 | 2 | 2 |  |
| Coatomer subunit delta | ARCN1 | P48444 | 57 210 | 2 | 2 | 2 |  |
| Extracellular superoxide dismutase [Cu-Zn] | SOD3 | P08294 | 25 851 | 2 | 2 | 2 |  |
| Lysosomal protective protein | CTSA | P10619 | 54 466 | 2 | 2 | 2 |  |
| Protein S100-A6 | S100A6 | P06703 | 10 180 | 1 | 2 | 2 |  |
| Putative hydroxypyruvate isomerase | HYI | Q5T013 | 30 406 | 3 | 2 | 2 |  |
| Thiopurine S-methyltransferase | TPMT | P51580 | 28 180 | 1 | 2 | 2 |  |
| 5-phosphohydroxy-L-lysine phospho-lyase | PHYKPL | Q8IUZ5 | 49 711 | 3 | 1 | 3 |  |
| Acidic leucine-rich nuclear phosphoprotein 32 family member A | ANP32A | P39687 | 28 585 | 2 | 1 | 3 |  |
| Adenylyl cyclase-associated protein 2 | CAP2 | P40123 | 52 824 | 2 | 1 | 2 | 1 |
| Corticosteroid-binding globulin | SERPINA6 | P08185 | 45 141 | 3 | 1 | 3 |  |
| Dorsal root ganglia homeobox protein | DRGX | A6NNA5 | 28 672 | 2 | 1 | 3 |  |
| GDP-fucose protein O-fucosyltransferase 2 | POFUT2 | Q9Y2G5 | 49 976 | 2 | 1 | 3 |  |
| Immunoglobulin kappa variable 1-27 | IGKV1-27 | A0A075B6S5 | 12 712 | 1 | 1 | 3 |  |
| Immunoglobulin lambda variable 1-47 | IGLV1-47 | P01700 | 12 284 | 1 | 1 | 3 |  |
| Isochorismatase domain-containing protein 2 | ISOC2 | Q96AB3 | 22 337 | 2 | 1 | 3 |  |
| Phenylalanine--tRNA ligase beta subunit | FARSB | Q9NSD9 | 66 116 | 3 | 1 | 3 |  |
| Protein PAXX | PAXX | Q9BUH6 | 21 640 | 2 | 1 | 3 |  |
| Protein phosphatase 1A | PPM1A | P35813 | 42 448 | 3 | 1 | 3 |  |
| Ras-related protein Rab-5B | RAB5B | P61020 | 23 707 | 2 | 1 | 3 |  |
| Twinfilin-2 | TWF2 | Q6IBS0 | 39 548 | 2 | 1 | 3 |  |
| Ubiquitin-conjugating enzyme E2 Z | UBE2Z | Q9H832 | 38 210 | 3 | 1 | 3 |  |
| 26S proteasome non-ATPase regulatory subunit 3 | PSMD3 | O43242 | 60 978 | 4 | - | 4 |  |
| 26S proteasome regulatory subunit 7 | PSMC2 | P35998 | 48 634 | 4 | - | 4 |  |
| Complement C4-A | C4A | P0C0L4 | 192 785 | 3 | - | 4 | - |
| Leucine carboxyl methyltransferase 1 | LCMT1 | Q9UIC8 | 38 379 | 3 | - | 3 | 1 |
| Replication protein A 70 kDa DNA-binding subunit | RPA1 | P27694 | 68 138 | 3 | - | 4 |  |
| Citrate synthase, mitochondrial | CS | O75390 | 51 712 | 4 |  | 4 |  |
| Eosinophil cationic protein | RNASE3 | P12724 | 18 385 | 3 |  | 4 |  |
| Fibrinogen beta chain | FGB | P02675 | 55 928 | 4 |  | 4 |  |
| Immunoglobulin heavy variable 4-28 | IGHV4-28 | A0A0C4DH34 | 13 124 | 4 |  | 4 |  |
| Ketosamine-3-kinase | FN3KRP | Q9HA64 | 34 412 | 3 |  | 4 |  |
| Leucine zipper transcription factor-like protein 1 | LZTFL1 | Q9NQ48 | 34 592 | 4 |  | 4 |  |
| Lipocalin-1 | LCN1 | P31025 | 19 250 | 2 |  | 4 |  |
| Phosphoribosylaminoimidazole carboxylase | PAICS | P22234 | 47 079 | 4 |  | 4 |  |
| T-complex protein 1 subunit zeta | CCT6A | P40227 | 58 024 | 4 |  | 4 |  |
| Cystatin-S | CST4 | P01036 | 16 214 | 7 | 1 | 3 |  |
| Putative tubulin-like protein alpha-4B | TUBA4B | Q9H853 | 27 551 | 2 | 1 | 2 |  |
| Ras-related C3 botulinum toxin substrate 2 | RAC2 | P15153 | 21 429 | 4 | 2 | 1 |  |
| Immunoglobulin heavy variable 3-15 | IGHV3-15 | A0A0B4J1V0 | 12 926 | 4 |  | 4 |  |
| Glutathione S-transferase A5 | GSTA5 | Q7RTV2 | 25 722 | 8 | 2 | 2 | 0 |
| GMP reductase 1 | GMPR | P36959 | 37 419 | 2 | 2 | 2 |  |
| GMP reductase 2 | GMPR2 | Q9P2T1 | 37 874 | 3 | 1 | 3 |  |
| Aldehyde dehydrogenase family 3 member B1 | ALDH3B1 | P43353 | 51 840 | 3 | 0 | 3 | 0 |
| Glycogen phosphorylase, liver form | PYGL | P06737 | 97 149 | 4 | 1 | 2 |  |
| L-lactate dehydrogenase A-like 6A | LDHAL6A | Q6ZMR3 | 36 507 | 3 | 1 | 2 |  |
| Immunoglobulin heavy variable 3-7 | IGHV3-7 | P01780 | 12 943 | 4 | 1 | 2 | 0 |
| Alanine aminotransferase 1 | GPT | P24298 | 54 637 | 3 | 3 |  |  |
| Annexin A8 | ANXA8 | P13928 | 36 881 | 2 | 3 |  |  |
| Palmitoyl-protein thioesterase 1 | PPT1 | P50897 | 34 193 | 3 | 3 |  |  |
| Vimentin | VIM | P08670 | 53 652 | 3 | 3 |  |  |
| 3-oxo-5-beta-steroid 4-dehydrogenase | AKR1D1 | P51857 | 37 377 | 1 | 2 | 1 |  |
| Bardet-Biedl syndrome 4 protein | BBS4 | Q96RK4 | 58 282 | 1 | 2 | 1 |  |
| Calmodulin-like protein 5 | CALML5 | Q9NZT1 | 15 893 | 2 | 2 | 1 |  |
| Calponin-3 | CNN3 | Q15417 | 36 414 | 2 | 2 | 1 |  |
| Carbonic anhydrase 12 | CA12 | O43570 | 39 451 | 1 | 2 | 1 |  |
| Caspase recruitment domain-containing protein 6 | CARD6 | Q9BX69 | 116 468 | 1 | 2 | 1 |  |
| EKC/KEOPS complex subunit LAGE3 | LAGE3 | Q14657 | 14 804 | 1 | 2 | 1 |  |
| Glutaredoxin-1 | GLRX | P35754 | 11 776 | 2 | 2 | 1 |  |
| Ly6/PLAUR domain-containing protein 2 | LYPD2 | Q6UXB3 | 13 115 | 1 | 2 | 1 |  |
| Polyadenylate-binding protein 1 | PABPC1 | P11940 | 70 671 | 2 | 2 | 1 |  |
| Protein cordon-bleu | COBL | O75128 | 135 617 | 1 | 2 | 1 | - |
| Ribose-5-phosphate isomerase | RPIA | P49247 | 33 269 | 2 | 2 | 1 |  |
| 26S proteasome non-ATPase regulatory subunit 10 | PSMD10 | O75832 | 24 428 | 2 | 1 | 2 |  |
| Bargin | BARGIN | Q6ZT62 | 73 599 | 1 | 1 | 2 |  |
| Branched-chain-amino-acid aminotransferase, mitochondrial | BCAT2 | O15382 | 44 288 | 2 | 1 | 2 |  |
| cAMP-dependent protein kinase type I-alpha regulatory subunit | PRKAR1A | P10644 | 42 982 | 2 | 1 | 2 |  |
| Caspase-1 | CASP1 | P29466 | 45 159 | 2 | 1 | 2 |  |
| Dehydrogenase/reductase SDR family member 11 | DHRS11 | Q6UWP2 | 28 308 | 2 | 1 | 2 |  |
| Delta(3,5)-Delta(2,4)-dienoyl-CoA isomerase, mitochondrial | ECH1 | Q13011 | 35 816 | 3 | 1 | 2 |  |
| Deoxyribonuclease-2-alpha | DNASE2 | O00115 | 39 581 | 1 | 1 | 2 |  |
| DNA repair protein RAD51 homolog 3 | RAD51C | O43502 | 42 190 | 1 | 1 | 1 | 1 |
| Dynein light chain 1, cytoplasmic | DYNLL1 | P63167 | 10 366 | 1 | 1 | 2 |  |
| Glutamate--cysteine ligase regulatory subunit | GCLM | P48507 | 30 727 | 2 | 1 | 2 |  |
| Golgi reassembly-stacking protein 2 | GORASP2 | Q9H8Y8 | 47 145 | 2 | 1 | 2 |  |
| High mobility group nucleosome-binding domain-containing protein 5 | HMGN5 | P82970 | 31 525 | 2 | 1 | 2 |  |
| Immunoglobulin heavy variable 1-3 | IGHV1-3 | A0A0C4DH29 | 13 008 | 2 | 1 | 2 |  |
| Immunoglobulin heavy variable 5-51 | IGHV5-51 | A0A0C4DH38 | 12 675 | 1 | 1 | 2 |  |
| Immunoglobulin kappa variable 3D-11 | IGKV3D-11 | A0A0A0MRZ8 | 12 625 | 1 | 1 | 2 |  |
| Immunoglobulin lambda variable 3-9 | IGLV3-9 | A0A075B6K5 | 12 332 | 1 | 1 | 2 |  |
| Inositol monophosphatase 2 | IMPA2 | O14732 | 31 321 | 2 | 1 | 2 |  |
| Inositol-3-phosphate synthase 1 | ISYNA1 | Q9NPH2 | 61 068 | 2 | 1 | 2 |  |
| Lysosomal alpha-mannosidase | MAN2B1 | O00754 | 113 744 | 2 | 1 | 2 |  |
| Melanocortin-2 receptor accessory protein 2 | MRAP2 | Q96G30 | 23 548 | 1 | 1 | 1 | 1 |
| MOB kinase activator 1B | MOB1B | Q7L9L4 | 25 091 | 2 | 1 | 2 |  |
| N-acetylmuramoyl-L-alanine amidase | PGLYRP2 | Q96PD5 | 62 217 | 2 | 1 | 2 |  |
| Ornithine aminotransferase, mitochondrial | OAT | P04181 | 48 535 | 2 | 1 | 2 |  |
| Phosphatidylinositol transfer protein alpha isoform | PITPNA | Q00169 | 31 806 | 1 | 1 | 2 |  |
| Probable tRNA N6-adenosine threonylcarbamoyltransferase | OSGEP | Q9NPF4 | 36 427 | 2 | 1 | 2 |  |
| Prostate stem cell antigen | PSCA | O43653 | 11 959 | 1 | 1 | 2 |  |
| Proteasome subunit beta type-2 | PSMB2 | P49721 | 22 836 | 2 | 1 | 2 |  |
| Protein farnesyltransferase/geranylgeranyltransferase type-1 subunit alpha | FNTA | P49354 | 44 409 | 2 | 1 | 2 |  |
| Pyridoxal phosphate phosphatase | PDXP | Q96GD0 | 31 698 | 2 | 1 | 2 |  |
| Small glutamine-rich tetratricopeptide repeat-containing protein alpha | SGTA | O43765 | 34 063 | 2 | 1 | 2 |  |
| SUMO-activating enzyme subunit 1 | SAE1 | Q9UBE0 | 38 450 | 2 | 1 | 2 |  |
| Thyroxine-binding globulin | SERPINA7 | P05543 | 46 325 | 3 | 1 | 2 |  |
| Tropomodulin-3 | TMOD3 | Q9NYL9 | 39 595 | 3 | 1 | 2 |  |
| Zinc finger protein 850 | ZNF850 | A8MQ14 | 125 431 | 1 | 1 | 1 | 1 |
| Stress-70 protein, mitochondrial | HSPA9 | P38646 | 73 680 | 2 | - | 3 |  |
| T-complex protein 1 subunit beta | CCT2 | P78371 | 57 488 | 3 | - | 3 |  |
| 26S proteasome regulatory subunit 6A | PSMC3 | P17980 | 49 204 | 3 |  | 3 |  |
| 4-aminobutyrate aminotransferase, mitochondrial | ABAT | P80404 | 56 439 | 3 |  | 3 |  |
| Acylamino-acid-releasing enzyme | APEH | P13798 | 81 225 | 3 |  | 3 |  |
| Beta-galactoside alpha-2,6-sialyltransferase 1 | ST6GAL1 | P15907 | 46 605 | 3 |  | 3 |  |
| cAMP-dependent protein kinase catalytic subunit alpha | PRKACA | P17612 | 40 590 | 2 |  | 3 |  |
| Deaminated glutathione amidase | NIT1 | Q86X76 | 35 896 | 3 |  | 3 |  |
| Endogenous retrovirus group K member 104 Rec protein | HERV-K104 | P61576 | 11 735 | 1 |  | 3 |  |
| Hydroxyacyl-coenzyme A dehydrogenase, mitochondrial | HADH | Q16836 | 34 294 | 3 |  | 3 |  |
| Hydroxymethylglutaryl-CoA lyase, mitochondrial | HMGCL | P35914 | 34 360 | 3 |  | 3 |  |
| Immunoglobulin heavy variable 1-18 | IGHV1-18 | A0A0C4DH31 | 12 820 | 3 |  | 3 |  |
| Nucleobindin-1 | NUCB1 | Q02818 | 53 879 | 3 |  | 3 |  |
| Proteasome subunit beta type-7 | PSMB7 | Q99436 | 29 965 | 2 |  | 3 |  |
| Ribonuclease T2 | RNASET2 | O00584 | 29 481 | 2 |  | 3 |  |
| SPRY domain-containing protein 4 | SPRYD4 | Q8WW59 | 23 129 | 3 |  | 3 |  |
| Radixin | RDX | P35241 | 68 564 | 11 | 1 | 2 | 0 |
| ADP-ribosylation factor 4 | ARF4 | P18085 | 20 511 | 3 | 1 | 1 | 0 |
| Small ubiquitin-related modifier 3 | SUMO3 | P55854 | 11 637 | 2 | 1 | 2 |  |
| Glial fibrillary acidic protein | GFAP | P14136 | 49 880 | 2 | 1 | 1 |  |
| Ras-related protein Rab-3A | RAB3A | P20336 | 24 984 | 3 | 1 | 1 |  |
| Ras-related protein Rab-3C | RAB3C | Q96E17 | 25 952 | 3 | 1 | 1 |  |
| Ras-related protein Rab-3D | RAB3D | O95716 | 24 267 | 3 | 1 | 1 |  |
| Transgelin-3 | TAGLN3 | Q9UI15 | 22 473 | 6 | 1 | 1 |  |
| Immunoglobulin heavy variable 3-74 | IGHV3-74 | A0A0B4J1X5 | 12 840 | 4 | 0 | 1 | 1 |
| Ubiquitin-conjugating enzyme E2 D2 | UBE2D2 | P62837 | 16 735 | 3 | 2 | 0 |  |
| Putative heat shock protein HSP 90-beta 2 | HSP90AB2P | Q58FF8 | 44 349 | 6 | 0 | 2 |  |
| Pyruvate kinase PKLR | PKLR | P30613 | 61 830 | 3 | 1 | 1 | 0 |
| Aldehyde dehydrogenase X, mitochondrial | ALDH1B1 | P30837 | 57 206 | 3 | 0 | 2 |  |
| Acylphosphatase-1 | ACYP1 | P07311 | 11 261 | 1 | 2 |  |  |
| Gamma-glutamylaminecyclotransferase | GGACT | Q9BVM4 | 17 329 | 2 | 2 |  |  |
| Lysosomal acid phosphatase | ACP2 | P11117 | 48 344 | 2 | 2 | - |  |
| NK-tumor recognition protein | NKTR | P30414 | 165 677 | 1 | 2 |  |  |
| Nuclear transport factor 2 | NUTF2 | P61970 | 14 478 | 2 | 2 |  |  |
| ADP-ribosylation factor-like protein 1 | ARL1 | P40616 | 20 418 | 1 | 1 | 1 |  |
| 2-aminomuconic semialdehyde dehydrogenase | ALDH8A1 | Q9H2A2 | 53 401 | 1 | 1 | 1 |  |
| Alpha-galactosidase A | GLA | P06280 | 48 767 | 1 | 1 | 1 |  |
| Apoptosis regulator BAX | BAX | Q07812 | 21 184 | 1 | 1 | 1 |  |
| Choline/ethanolamine kinase | CHKB | Q9Y259 | 45 271 | 2 | 1 | 1 |  |
| Complement factor I | CFI | P05156 | 65 750 | 1 | 1 | 1 |  |
| Cytochrome c oxidase copper chaperone | COX17 | Q14061 | 6 915 | 1 | 1 | 1 |  |
| Dedicator of cytokinesis protein 11 | DOCK11 | Q5JSL3 | 237 671 | 1 | 1 | 1 |  |
| DNA-directed RNA polymerase II subunit RPB1 | POLR2A | P24928 | 217 176 | 1 | 1 | 1 |  |
| Elongation factor Tu, mitochondrial | TUFM | P49411 | 49 542 | 1 | 1 | 1 |  |
| F-box/WD repeat-containing protein 9 | FBXW9 | Q5XUX1 | 54 115 | 1 | 1 | 1 |  |
| FGGY carbohydrate kinase domain-containing protein | FGGY | Q96C11 | 59 993 | 1 | 1 | 1 |  |
| Gamma-glutamyl hydrolase | GGH | Q92820 | 35 964 | 1 | 1 | 1 |  |
| Ganglioside GM2 activator | GM2A | P17900 | 20 838 | 1 | 1 | 1 |  |
| GTP cyclohydrolase 1 feedback regulatory protein | GCHFR | P30047 | 9 698 | 1 | 1 | 1 |  |
| Heat shock 70 kDa protein 4L | HSPA4L | O95757 | 94 512 | 1 | 1 | 1 |  |
| HIG1 domain family member 1C | HIGD1C | A8MV81 | 11 079 | 1 | 1 | 1 |  |
| Immunoglobulin kappa variable 2-24 | IGKV2-24 | A0A0C4DH68 | 13 079 | 1 | 1 | 1 |  |
| Immunoglobulin kappa variable 6D-21 | IGKV6D-21 | A0A0A0MT36 | 12 340 | 1 | 1 | 1 |  |
| Inositol polyphosphate 1-phosphatase | INPP1 | P49441 | 43 998 | 1 | 1 | 1 |  |
| Isopentenyl-diphosphate Delta-isomerase 1 | IDI1 | Q13907 | 26 319 | 1 | 1 | 1 |  |
| Kynureninase | KYNU | Q16719 | 52 352 | 1 | 1 | 1 |  |
| L-xylulose reductase | DCXR | Q7Z4W1 | 25 913 | 1 | 1 | 1 |  |
| Lysophospholipase-like protein 1 | LYPLAL1 | Q5VWZ2 | 26 316 | 1 | 1 | 1 |  |
| NADP-dependent malic enzyme, mitochondrial | ME3 | Q16798 | 67 068 | 1 | 1 | 1 |  |
| N-sulphoglucosamine sulphohydrolase | SGSH | P51688 | 56 695 | 1 | 1 | 1 |  |
| Nucleosome assembly protein 1-like 4 | NAP1L4 | Q99733 | 42 823 | 1 | 1 | 1 |  |
| Phospholipase D3 | PLD3 | Q8IV08 | 54 705 | 1 | 1 | 1 |  |
| Protein CutA | CUTA | O60888 | 19 116 | 1 | 1 | 1 |  |
| Protein SETSIP | SETSIP | P0DME0 | 34 882 | 2 | 1 | 1 |  |
| SH3 domain-binding glutamic acid-rich-like protein 2 | SH3BGRL2 | Q9UJC5 | 12 326 | 1 | 1 | 1 |  |
| Short-chain specific acyl-CoA dehydrogenase, mitochondrial | ACADS | P16219 | 44 297 | 1 | 1 | 1 |  |
| Sorting nexin-3 | SNX3 | O60493 | 18 762 | 1 | 1 | 1 |  |
| Thioredoxin domain-containing protein 12 | TXNDC12 | O95881 | 19 206 | 1 | 1 | 1 |  |
| Tight junction protein ZO-3 | TJP3 | O95049 | 101 397 | 1 | 1 | 1 |  |
| Tripeptidyl-peptidase 1 | TPP1 | O14773 | 61 248 | 1 | 1 | 1 |  |
| Tumor necrosis factor ligand superfamily member 13B | TNFSF13B | Q9Y275 | 31 223 | 1 | 1 | 1 |  |
| Ubiquilin-2 | UBQLN2 | Q9UHD9 | 65 696 | 1 | 1 | 1 |  |
| Ubiquitin-like modifier-activating enzyme 5 | UBA5 | Q9GZZ9 | 44 863 | 1 | 1 | 1 |  |
| UBX domain-containing protein 1 | UBXN1 | Q04323 | 33 325 | 1 | 1 | 1 |  |
| Unconventional myosin-VIIb | MYO7B | Q6PIF6 | 241 599 | 1 | 1 | 1 |  |
| CXXC motif containing zinc binding protein | C1orf123 | Q9NWV4 | 18 048 | 1 | 1 | 1 |  |
| Uroporphyrinogen decarboxylase | UROD | P06132 | 40 787 | 1 | 1 | 1 |  |
| WD repeat-containing protein 64 | WDR64 |  | 123 631 | 1 | 1 | 1 |  |
| 26S proteasome non-ATPase regulatory subunit 11 | PSMD11 | O00231 | 47 464 | 2 | - | 2 |  |
| Advillin | AVIL | O75366 | 92 027 | 2 | - | 2 |  |
| Apoptosis-inducing factor 1, mitochondrial | AIFM1 | O95831 | 66 901 | 2 | - | 2 |  |
| F-box/LRR-repeat protein 8 | FBXL8 | Q96CD0 | 40 516 | 2 | - | 2 |  |
| Neurotrypsin | PRSS12 | P56730 | 97 067 | 2 | - | 2 |  |
| Peptidyl-alpha-hydroxyglycine alpha-amidating lyase | PAM | P19021 | 108 332 | 2 | - | 2 |  |
| Polypyrimidine tract-binding protein 1 | PTBP1 | P26599 | 57 221 | 2 | - | 2 |  |
| Serine/threonine-protein phosphatase 2B catalytic subunit beta isoform | PPP3CB | P16298 | 59 024 | 2 | - | 2 |  |
| V-type proton ATPase catalytic subunit A | ATP6V1A | P38606 | 68 304 | 2 | - | 2 |  |
| 26S proteasome regulatory subunit 10B | PSMC6 | P62333 | 44 173 | 1 |  | 2 |  |
| 3-ketoacyl-CoA thiolase, peroxisomal | ACAA1 | P09110 | 44 292 | 2 |  | 2 |  |
| Adenylyl-sulfate kinase | PAPSS1 | O43252 | 70 833 | 2 |  | 2 |  |
| Bleomycin hydrolase | BLMH | Q13867 | 52 562 | 2 |  | 2 |  |
| Charged multivesicular body protein 4b | CHMP4B | Q9H444 | 24 950 | 2 |  | 2 |  |
| Cilia- and flagella-associated protein 52 | CFAP52 | Q8N1V2 | 68 298 | 1 |  | 2 |  |
| ELAV-like protein 1 | ELAVL1 | Q15717 | 36 092 | 2 |  | 2 |  |
| Elongin-C | ELOC | Q15369 | 12 473 | 2 |  | 2 |  |
| Glutamine synthetase | GLUL | P15104 | 42 064 | 2 |  | 2 |  |
| Heterogeneous nuclear ribonucleoprotein Q | SYNCRIP | O60506 | 69 603 | 2 |  | 2 |  |
| Hydroxymethylglutaryl-CoA synthase, cytoplasmic | HMGCS1 | Q01581 | 57 294 | 2 |  | 2 |  |
| Immunity-related GTPase family Q protein | IRGQ | Q8WZA9 | 62 717 | 2 |  | 2 |  |
| Immunoglobulin lambda variable 3-25 | IGLV3-25 | P01717 | 12 011 | 2 |  | 2 |  |
| Intelectin-1 | ITLN1 | Q8WWA0 | 34 962 | 2 |  | 2 |  |
| Inter-alpha-trypsin inhibitor heavy chain H1 | ITIH1 | P19827 | 101 389 | 2 |  | 2 |  |
| Isocitrate dehydrogenase [NAD] subunit alpha, mitochondrial | IDH3A | P50213 | 39 592 | 1 |  | 2 |  |
| Phosphatidylinositol transfer protein beta isoform | PITPNB | P48739 | 31 540 | 2 |  | 2 |  |
| Prostasin | PRSS8 | Q16651 | 36 431 | 1 |  | 2 |  |
| Proteasome subunit beta type-10 | PSMB10 | P40306 | 28 936 | 1 |  | 2 |  |
| Protein disulfide-isomerase A6 | PDIA6 | Q15084 | 48 121 | 2 |  | 2 |  |
| Protein FAM3C | FAM3C | Q92520 | 24 680 | 2 |  | 2 |  |
| Protein unc-119 homolog B | UNC119B | A6NIH7 | 28 137 | 2 |  | 2 |  |
| Threonine--tRNA ligase, cytoplasmic | TARS | P26639 | 83 435 | 2 |  | 2 |  |
| Putative protein FAM10A5 | ST13P5 | Q8NFI4 | 41 378 | 3 | 0 | 2 |  |

**Supplemental Table 6. Human nasal mucus proteome.** The gene name, UniProt code access, molecular weight, number of peptides, and number of spectra identified per donor for each protein. The proteins are ranked in ascending order according to the total number of spectra identified.
